# Discovery of Anti-Inflammatory Alkaloids from Sponge *Stylissa massa* Suggests New Biosynthetic Pathways for Pyrrole–Imidazole Alkaloids

**DOI:** 10.3390/md22100477

**Published:** 2024-10-18

**Authors:** Xiaojing Liu, Qi Wang, Yun Zhang, Hanting Zhang

**Affiliations:** 1 Department of Natural Medicinal Chemistry and Pharmacognosy, School of Pharmacy, Qingdao University, Qingdao 266021, China; liuxiaojing2@qdu.edu.cn (X.L.); wangqi@hmfl.ac.cn (Q.W.); 2 Biology Institute, Qilu University of Technology (Shandong Academy of Sciences), Jinan 250103, China

**Keywords:** pyrrole–imidazole alkaloids, seco-spongiacidin, latonduine, *Stylissa massa*, biosynthetic pathway, anti-inflammatory

## Abstract

Pyrrole–imidazole alkaloids (PIAs) are a class of marine sponge derived natural products which have complex carbon frameworks and broad bioactivities. In this study, four new alkaloids, stylimassalins A–B (**1**–**2**), **3**, and **5**, together with two known compounds (**4** and **6**), were isolated from *Stylissa massa*. Compounds **2**, **4**, and **6** are the C-2 brominated analogues of **1**, **3**, and **5**, respectively. Their structures display three different scaffolds, of which scaffold 1 (compounds **1**,**2**) is new. A new biosynthetic pathway from oroidin, through spongiacidin, to latonduine and scaffold 1 was proposed by our group, in which the C12-N13-cleavaged compounds of spongiacidin (scaffold 2), dubbed seco-spongiacidins (**3** and **4**), are recognized as a key bridged scaffold, to afford PIA analogues (**1**,**2** and **5**,**6**). An anti-inflammatory evaluation in a zebrafish inflammation model induced by copper sulphate (CuSO_4_) demonstrated that stylimassalins A and B (**1** and **2**) could serve as a promising lead scaffold for treating inflammation.

## 1. Introduction

Pyrrole–imidazole alkaloids (PIAs) are a huge family of marine sponges derived natural products that are marked by structural complexity and biological activity [1,2]. Since the identification of the first PIA, (-)-dibromophakellin, in 1971 [3], hundreds of additional PIAs have been discovered and extensive research on their structural elucidation, biological/chemical syntheses, and bioactivities have been conducted [4,5]. The chemical origin of PIA structures can be traced back to the specific amino acids, proline and lysine, and then transitions to oroidin, a key biogenetic precursor of PIAs. A large majority of documented PIA events could benefit from the chemical transformations of oroidin [6]. Oroidin functions as the basis of structural diversity for the carbon skeleton by undergoing various transformations, such as cyclization and dimerization, to produce complicated PIAs such as palau’amine [7] and stylissadine [8]. Among the multifarious PIA community, monomer ones, including spongiacidin [9], agelastatin [10], phakellin [3], and latonduine [11], have been discovered sequentially in recent decades.

**Figure 1:**
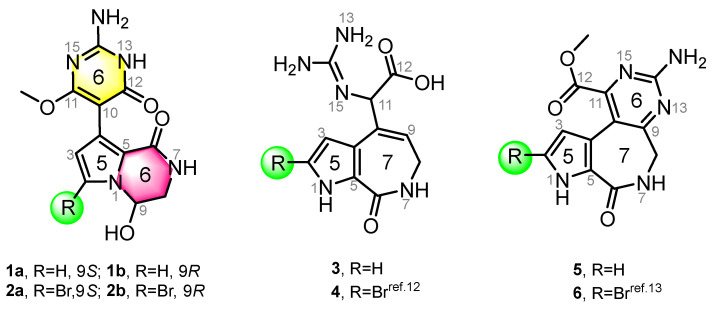
Chemical structures of compounds **1**–**6**. Stylimassalins A and B (**1** and **2**) (with a 5/6/6 scaffold) and compounds **3** and **4** are 12,13-secospongiacidin PIAs (with a 5/7 scaffold), and compounds **5** and **6** are latonduine PIAs (with a 5/7/6 scaffold).

In this study, four new and two known alkaloids (Figure 1) were recovered from the sponge *S. massa* (Figure S1). Compounds **1** and **2** share the same 5/6/6 tricyclic system, featuring a unique connection between a 3,4-dihydropyrrolo[1,2-*a*]pyrazin-1(2*H*)-one system and a pyrimidine moiety, with the only difference being the presence or absence of bromination at C-2. This is consistent with previous studies noting that PIAs are often accompanied by bromination on their pyrrole ring system. Compounds **1** and **2** (scaffold 1) are not strictly PIAs because they have a rearranged pyrimidine system rather than an imidazole ring. Compounds **3** and **4** [12] are two spongiacidin PIA analogues with a C12-N13 bond cleavage, dubbed secospongiacidin PIAs (scaffold 2). Compounds **5** and **6** [13] belong to the latonduine family of PIA (scaffold 3). A new biosynthetic pathway from oroidin, through spongiacidin, to latonduine and scaffold 1 was proposed in this study, in which seco-spongiacidins (**3** and **4**, scaffold 2) are recognized as a key bridged scaffold in the formation of latonduine PIA analogues (**5** and **6**).

## 2. Results

### 2.1. Structural Elucidation

Stylimassalin A (**1**) was obtained as a yellow oil. In the HRESIMS spectrum of **1**, a [M + H]^+^ ion peak at *m*/*z* 292.1036 was observed, which determined its molecular formula to be C_12_H_13_N_5_O_4_ (calculated for [M + H]^+^ as 292.1040) with nine degrees of unsaturation. In the ^1^H NMR spectrum of stylimassalin A (**1**), acquired in DMSO-*d*_6_, there was only one-NH proton signal at *δ*_H_ 7.38, with the regular 1-NH of the pyrrole system at approximately *δ*_H_ 12 not observed. Further analysis of the ^1^H, ^13^C NMR (Tables S1 and S2), and HSQC spectra revealed three methines (*δ*_H-2_ 6.93, d, *J* = 2.6 Hz, *δ*_C-2_ 120.2; *δ*_H-3_ 6.02, d, *J* = 2.6 Hz, *δ*_C-3_ 112.3; *δ*_H-9_ 5.54, t, *J* = 3.5 Hz, *δ*_C-9_ 74.4), one methylene group (*δ*_H-8a_ 3.29, ddd, *J* = 13.2, 7.4, 3.5 Hz, *δ*_H-8b_ 3.54, dd, *J* = 13.0, 2.1 Hz, *δ*_C-8_ 46.3), one methyl (*δ*_11-OMe_ 3.65, s, *δ*_11-OMe_ 53.3), and additional non-protonated carbons (*δ*_C-5_ 119.8; *δ*_C-6_ 119.3; *δ*_C-10_ 89.5; *δ*_C-12_ 159.4; *δ*_C-14_ 153.7; *δ*_C-11_ 167.0). The ^1^H–^1^H COSY correlations of H-2 and H-3, together with the HMBC correlations of H-2 with C-3 and C-5 and of H-3 with C-2 and C-5 indicated the presence of a N-substituted pyrrole ring system. The other COSY correlations of 7-NH (*δ*_H_ 7.38), H_2_-8 (*δ*_H_ 3.29, 3.54), and H-9 (*δ*_H_ 5.54) indicated a NH-CH_2_-CH molecular fragment. There are no more available 2D NMR correlations in the HMBC spectrum of **1** except for a key correlation between the methyl (*δ*_H_ 3.65) and a quaternary aromatic carbon (*δ*_C_ 167.0). We were confused while determining the structure of stylimassalin A (**1**), until the discovery of stylimassalin B (**2**).

**Figure 2:**
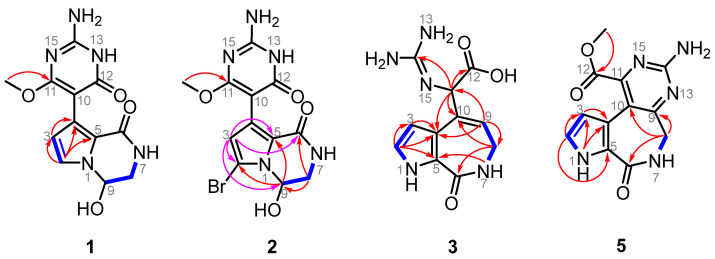
Structural elucidation of compounds **1**, **2**, **3**, and **5**. HMBC correlations are shown using red (acquired via DMSO-*d*_6_) and pink (acquired via methanol-*d*_4_) arrow lines and COSY correlations are shown using blue bold lines.

Stylimassalin B (**2**) was obtained as a colourless crystal. Its molecular formula was deduced as C_12_H_12_BrN_5_O_4_ based on the HRESIMS ions at *m*/*z* 370.0135 and 372.0115 (1:1) [M + H]^+^ (calcd. 370.0145, 372.0125), which indicated the presence of a bromine atom. Detailed analysis of the 1D and 2D NMR spectra (Tables S1 and S2, Figure 2) of both **1** and **2** in DMSO-*d*_6_ indicated that their structures were very similar except for the difference in C-2. Obvious high-field movement of the chemical shift of C-2 in compound **2** (**2**: *δ*_C-2_ 101.9; **1**: *δ*_C-2_ 120.2) revealed that the planar structure of **2** is a C-2 brominated analogue of stylimassalin A (**1**).

**Figure 3:**
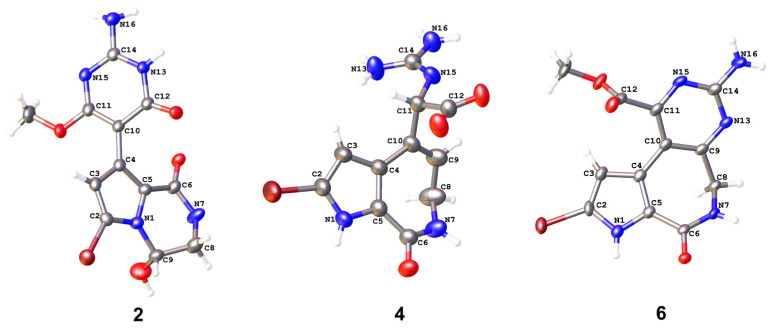
X-ray structures of compounds **2**, **4**, and **6**, which have three different scaffolds. The data on compound **4** have been published [12].

To determine the structures of stylimassalins A-B (**1** and **2**), single-crystal culturing using a slow solvent evaporation method was carried out over a long time period. Eventually, a qualified colourless crystal of **2** was obtained for crystallographic analyses (90% ethanol aqueous solution, Table S3). The results of its X-ray diffraction experiment gave the structure of **2** (CCDC2249955 for **2**, Figure 3). Detailed analysis of the 1D and 2D NMR data of **2** (Figure 2, in methanol-*d*_4_ solvent) and the HMBC correlations of H-3 (*δ*_H_ 6.24) with C-2 (*δ*_C_ 105.3), C-4 (*δ*_C_ 124.1), C-5 (*δ*_C_ 123.0), C-6 (*δ*_C_ 161.8), C-9 (*δ*_C_ 74.4), and C-10 (*δ*_C_ 90.4) indicated the presence of a 2twobrominated N-substituted pyrrole ring system. Additionally, the HMBC correlations of H_2_-8 (*δ*_H_ 3.56, 3.79) with C-6 and C-9 and H-9 (*δ*_H_ 5.75) with C-2 and C-5 disclosed the substructure of **2**: a 2-bromo-9-hydroxy-3,4-dihydropyrrolo[1,2-*a*]pyrazin-1(2*H*)-one system. Then, a key HMBC correlation of 11-OMe (*δ*_H_ 3.79) with C-11 (*δ*_C_ 170.3) determined the -OMe moiety substituted at C-11 of **2**. Thus, all 1D and 2D NMR data on both **1** and **2** were matched and explainable, which is consistent with the X-ray diffraction results.

**Figure 4:**
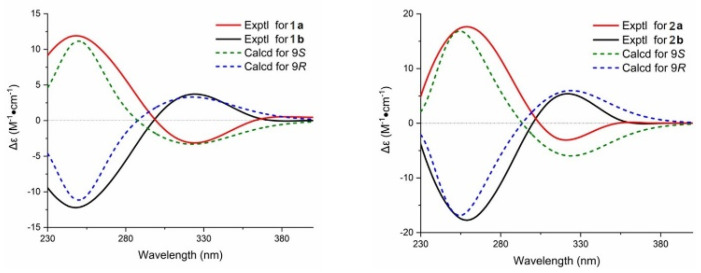
The absolute configurations of **1** and **2** were determined by time-dependent density functional theory ECD (TDDFT-ECD) calculations (9*S* for **1a** and **2a**; 9*R* for **1b** and **2b**).

In addition, the crystal of **2** possessed a P1 21/n1 space group, indicating the possible racemic nature of **1** and **2** [14]. To determine the absolute configuration of them, the chiral HPLC isolation of **1** and **2** was carried out, which resulted in an equimolar pair of optically pure enantiomers (Figures S6 and S7). Their absolute configurations were determined by time-dependent density functional theory ECD (TDDFT-ECD) calculations (9*S* for **1a** and **2a**, 9*R* for **1b** and **2b**; Figure 4 and Figure S8).

Compound **3** was obtained as a white powder. Detailed analysis of the 1D and 2D NMR spectra of **3** indicated that it is a structure very similar to compound **4**, a compound previously isolated by our group (CCDC2172264 for **4**; Figure 3 and Table S4) [12]. The molecular formula of **3** was deduced to be C_11_H_13_N_5_O_3_ based on the HRESIMS ions at *m*/*z* 264.1087 [M + H]^+^ (calcd. 264.1091), which possess one less bromine atom than compound **4** (the HRESIMS ions at *m*/*z* 342.0184, 344.0162, 1:1). The only difference between them is that there is either bromination or not at C-2 in **4** (*δ*_C-2_ 104.3) or **3** (*δ*_C-2_ 122.1). Although they are racemates for compound **3**, with no ECD curve, the same as with compound **4** [12], the enantiomers of **3** were not separated and then characterized for an absolute configuration assignment due to their limited amount.

In addition, two latonduine-type PIAs (compounds **5** and **6** [13]) were co-isolated from the sponge *S. massa*. Detailed analysis of their 1D and 2D NMR data revealed compound **6** to be the C-2 brominated analogue of compound **5**. The chemical shifts of C-2 in their ^13^C NMR spectra were identified as *δ*_C_ 122.6 (compound **5**) and *δ*_C_ 104.7 (compound **6**). The X-ray structure of compound **6** was obtained for the first time in our study (CCDC2331670 for **6**, Figure 3, and Table S5).

### 2.2. Proposed Biosynthetic Pathway

**Scheme 1:**
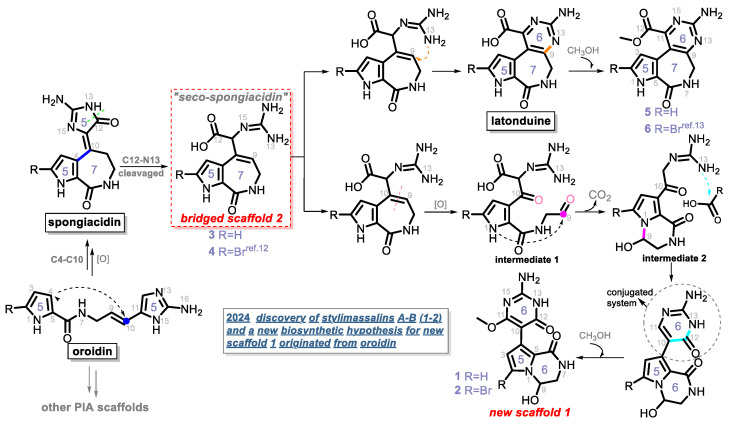
A new biosynthetic hypothesis of compounds **1**–**6**.

Based on the above description, compounds **1**–**6**, including two different structural skeletons, were isolated from the sponge together. There seems an obvious relationship among them, as they share a common biosynthetic lineage from the same C_11_N_5_ biosynthetic precursor, oroidin. Spongiacidin PIAs possess a 5/7/5 tricyclic scaffold, which might be generated by the C4-C10 Michael addition of oroidin. Latonduine is a small PIA family, and it has only contained approximately ten members since the latonduines A and B were first isolated from the sponge *Stylissa carteri* [11,13]. A previous report [11] suggested that latonduine PIAs cannot be derived from the C_11_N_5_ building block of oroidin; however, we have a different opinion (Scheme 1).

Compound **4** was previously isolated by our group in 2022 [12]. We ignored its critical role in the biosynthesis of PIAs until the co-isolation of compounds **1**–**6** in this study. A new biosynthetic pathway from oroidin, through spongiacidin, to latonduine was proposed in Scheme 1, in which the C12-N13-cleavaged compounds of spongiacidin, dubbed seco-spongiacidins (**3** and **4**), are recognized as a key bridged scaffold that creates latonduine PIA analogues (**5** and **6**). The biosynthetic pathway from the bridged scaffold 2 to the new scaffold 1 was relatively more complex. Firstly, the cleavage of C9-C10 generated intermediate 1. Then, a mechanism involving intramolecular cyclization and decarboxylation steps was proposed to obtain intermediate 2. Through a new round of nucleophilic addition from 13-NH to a carbonyl moiety, a stable conjugated system of 2-aminopyrimidin-4(3H)-one (indicated by the circular dashed line in Scheme 1) was formed, which obtained the new scaffold 1 (5/6/6 tricyclic system). Then, the compounds **1** and **2** were finally biosynthesised by further methoxylation at C-11.

### 2.3. Biological Activity

**Figure 5:**
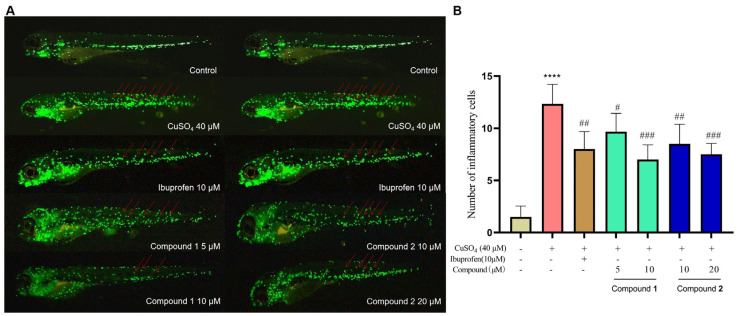
Compounds **1** and **2** inhibit CuSO_4_-induced inflammatory responses in zebrafish. (**A**) Representative fluorometric images of macrophage migration in the CuSO_4_-induced inflammation model. The red arrows indicate macrophages that migrated to the lateral line neural crest. (**B**) Statistical analysis of the number of macrophages in the lateral line neural crest. The data are presented as the means ± SEMs (n = 6). *^#^ p* < 0.05, ^##^ *p* < 0.01, and ^###^ *p* < 0.001 compared with the control group; **** *p* < 0.0001 compared with the model group.

PIAs have been reported to possess a wide range of biological activities. In our earlier study, spongiacidin PIAs were determined to have inhibitory activities against aldose reductase (AKR1B1), a target of complications of diabetes [12,15]. So, compounds **1**–**6** were subjected to an AKR1B1-targeted inhibitory activity assay in vitro at a concentration of 20 µM (Figure S9). None of them (**1**–**6**) shown a significant activity (Figure S10). Then, the anti-inflammatory activities of compounds **1**–**6** were evaluated in a zebrafish inflammation model induced by copper sulphate (CuSO_4_). In the CuSO_4_-induced inflammation model, a significant migration of macrophages towards the lateral line neural crest was observed (Figure 5A). Compared with the CuSO_4_-induced inflammation model group, treatment with ibuprofen (10 µM) significantly inhibited the migration of macrophages in the positive control group, indicating the establishment of an inflammation model in zebrafish. The results show that stylimassalins A and B (**1** and **2**) significantly inhibited CuSO_4_-induced macrophage migration at 5 µM and 10 µM, respectively (Figure 5A,B), while compounds **3**–**6** had no effect.

## 3. Materials and Methods

### 3.1. General Experimental Procedures

Optical rotations were obtained on a Jasco P-1020 polarimeter (Tokyo, Japan). UV spectra were recorded using an Agilent 1260 DAD detector (Santa Clara, CA, USA). CD spectra were recorded using an Applied Photophysics Chirascan circular dichroism spectrometer (Bath, UK). NMR spectra were measured on Agilent Pro pulse 500 MHz and Bruker 600 MHz spectrometers (Santa Clara, CA, USA). Chemical shifts are reported with the residuals DMSO-*d*_6_ (*δ*_H_ 2.50 ppm) and methanol-*d*_4_ (*δ*_H_ 3.31 ppm) as the internal standards for ^1^H NMR spectroscopy and DMSO-*d*_6_ (*δ*_C_ 39.5 ppm) and methanol-*d*_4_ (*δ*_C_ 49.0 ppm) for ^13^C NMR spectroscopy. Electrospray ionization mass spectrometry (ESIMS) and HRESIMS were carried out on a ThermoFisher 480 MS instrument (Waltham, MA, USA). Commercial silica gel (Qingdao Haiyang Chemical Co., Ltd., Qingdao, China, 200–300 mesh, 300–400 mesh) and reversed-phase C18 (Rp-C18) silica gel (12 nm, S-50 μm, YMC Co., Ltd., Tokyo, Japan) were used for column chromatography, and precoated Silica gel GF254 plates (Sinopharm Chemical Reagent Co., Shanghai, China) were used for analytical TLC. Reversed-phase (RP) HPLC was performed on an Agilent 1260 series liquid chromatograph equipped with a DAD G1315D detector at 210, 230, 254, 280, 320, and 360 nm wavelengths (Agilent, Santa Clara, CA, USA). An Agilent ZORBAX Eclipse XDB-C18 column (9.4 × 250 mm, 5 μm) was employed for purification. A chiral HPLC analysis was carried out on a Jasco LC-Net II/ADC HPLC-CD combined instrument with a CHIRALPAK AD-H (5 μm, 4.6 mm I.D. × 250 mm) column. All solvents used for column chromatography and HPLC were of analytical grade (Tianjin Kemio Chemical Reagent Co., Ltd., Tianjin, China) and chromatographic grade (Tianjin Concord Technology Co., Ltd., Tianjin, China), respectively.

### 3.2. Collection of Sponge Materials

The marine sponge *Stylissa massa* was collected from the Xisha Islands of the South China Sea in May 2013 and was frozen immediately after collection. The specimen was identified by Dr. Nicole J. de Voogd, National Museum of Natural History, Leiden, The Netherlands. A voucher specimen (No. XS-2013-07) was deposited at lab A1007, School of Pharmacy, Qingdao University, China.

### 3.3. Extraction and Isolation of Compounds from S. massa

The *Stylissa massa* sponge (8.0 kg, wet weight) was crushed and then extracted with MeOH four times at room temperature. The combined solutions were concentrated in vacuo and the organic extract was evaporated to produce a brown residue (191.0 g). Then, to remove lipids and other impurities, alkaloid enrichment was carried out by silica gel vacuum liquid chromatography (VLC), eluting with a gradient of petroleum ether/EtOAc (from 10:1 to 0:1, *v*:*v*) and, subsequently, CH_2_Cl_2_/MeOH (from 10:1 to 0:1, *v*:*v*). The alkaloid-containing fractions were combined and evaporated, yielding a crude alkaloid extract (78.9 g).

Guided by the MS and UV-Vis spectra, several runs of purification on the RP-LC [MeOH (0.1%TFA)/H_2_O (0.1%TFA), from 5:95 to 100:0, *v*:*v*, 120 min] afforded the subfraction A (500 mg), which had an undescribed molecular weight and undescribed UV-Vis patterns. Subfraction A (400 mg), which contained compound **2**, was purified by semi-preparative HPLC [MeOH (0.1%TFA)/H_2_O (0.1%TFA), from 5:95 to 100:0, *v*:*v*, 1.5 mL/min], yielding compound **2** (2.0 mg, *t*_R_ = 32.4 min). Subfraction B (200 mg) contained compound **1**, which has an identical UV-Vis spectra, and was purified by semi-preparative HPLC [MeOH (0.1%TFA)/H_2_O (0.1%TFA), from 5:95 to 100:0, *v*:*v*, 1.5 mL/min], yielding compound **1** (3.0 mg, *t*_R_ = 21.8 min). Subfraction C (600 mg) contained compounds **3**–**4** and was purified by semi-preparative HPLC [MeOH (0.1%TFA)/H_2_O (0.1%TFA), from 5:95 to 100:0, *v*:*v*, 1.5 mL/min], yielding compound **3** (3.0 mg, *t*_R_ = 16.6 min) and compound **4** (3.0 mg, *t*_R_ = 21.5 min). Subfraction D (900 mg) contained compounds **5**–**6** and was purified by semi-preparative HPLC [MeOH (0.1%TFA)/H_2_O (0.1%TFA), from 5:95 to 100:0, *v*:*v*, 1.5 mL/min], yielding compound **5** (5.0 mg, *t*_R_ = 23.2 min) and compound **6** (11.0 mg, *t*_R_ = 39.0 min).

#### 3.3.1. Compound **1**

Yellow oil; (+)-**1**, [*α*]^20^_D_ + 31 (MeOH), (-)-**1**, [*α*]^20^_D_ − 29 (MeOH); UV (MeOH) *λ*_max_ 200, 230, 280 nm, Figure S5; ECD (MeOH) *λ*_max_ 249, 320 nm, Figure S8; ^13^C NMR data, Table S2, ^1^H NMR data, Table S1; HRESIMS *m*/*z* 292.1036 [M + H]^+^ (calcd for C_12_H_14_N_5_O_4_, 292.1040).

#### 3.3.2. Compound **2**

Colourless crystal; (+)-**2**, [*α*]^20^_D_ + 21 (MeOH), (-)-**2**, [*α*]^20^_D_ − 16 (MeOH); UV (MeOH) *λ*_max_ 200, 230, 280 nm, Figure S5; ECD (MeOH) *λ*_max_ 256, 315 nm, Figure S8; ^13^C NMR data, Table S2, ^1^H NMR data, Table S1; HRESIMS *m*/*z* 370.1035, 372.0115, 1:1 [M + H]^+^ (calcd for C_12_H_13_BrN_5_O_4_, 292.1040).

#### 3.3.3. Compound **3**

White powder; racemic mixture; UV (MeOH) *λ*_max_ 200, 228, 290 nm, Figure S5; ^13^C NMR data, Table S2, ^1^H NMR data, Table S1; HRESIMS *m*/*z* 264.1087 [M + H]^+^ (calcd for C_11_H_14_N_5_O_3_, 264.1091).

#### 3.3.4. Compound **5**

Yellow oil; UV (MeOH) *λ*_max_ 200, 250, 278 nm, Figure S5; ^13^C NMR data, Table S2, ^1^H NMR data, Table S1; HRESIMS *m*/*z* 274.0930 [M + H]^+^ (calcd for C_12_H_12_N_5_O_3_, 274.0935).

### 3.4. X-Ray Crystallographic Analysis for Compounds ***2***, ***4*** and ***6***

The crystals of **2**, **4**, and **6** were recrystallized. X-ray analyses were carried out on a Rigaku Xtalab Synergy diffractometer with Cu K*α* radiation (λ = 1.54184) at 150 K/293 K. The acquisition parameters for **2**, **4**, and **6** are provided in the Supporting Information, and their crystallographic data (deposition no. **2**: 2249955; **4**: 2172264; **6**: 2331670) have been deposited at the Cambridge Crystallographic Data Center. Copies of the data can be obtained free of charge via www.ccdc.cam.ac.uk/conts/retrieving.html (accessed on 11 February 2024).

### 3.5. AKR1B1-Targeted Inhibitory Activity Assay

In the first step of the polyol pathway, glucose reduction is accompanied by the conversion of NADPH to NADP, where NADPH has a spectral absorption around 340 nm and NADP does not. Thus, the decrease in OD_340nm_ can represent the consumption of NADPH. Therefore, we can detect the change in OD_340 nm_ before and after the reaction to screen the effective ARIs or evaluate AR activity; epalrestat was used as the positive control. Briefly, the incubation system contains 10 µL of AKR1B1 enzyme (20 µg/mL), 80 of µL DL-glyceraldehyde (0.16 mmol/L) as the substrate, 1.0 mmol/L of NADPH·4Na as a coenzyme, and 0.1 mol/L of PBS (pH = 6.2). The incubation mixture was minimized to a total volume of 100 µL and transferred into a 96-well ultraviolet plate. Then, the test wells were treated with the tested compounds (at a concentration of 20 µM) for 10 min at 25 °C. Then, the absorbance was measured using FlexStation 3 Reader (Molecular Devices, San Francisco, CA, USA) at 340 nm. The absorbance of wells treated with PBS and NADPH was considered to be 100% (OD_1_), and the absorbance of wells treated with PBS and DL-glyceraldehyde was considered to be 0% (OD_2_); the inhibitory rate was calculated by the formula OD compounds − OD_2_/OD_1_ − OD_2_. IC_50_ represents the concentration that inhibits the AKR1B1 enzyme by 50%.

### 3.6. The Anti-Inflammatory Effect of the Compounds Was Assessed Utilizing a CuSO_4_-Induced Inflammation Model

Healthy zebrafish larvae (3 dpf) were transferred to 24-well culture plates (10 per well). The zebrafish were grouped into a control group maintained in zebrafish culture water, a model group, a positive control group treated with a 10 μM ibuprofen solution, and experimental groups treated with compounds at different doses (5, 10, and 20 μM). The plates were maintained at 28 °C in an incubator, and the solution was replaced every 24 h. At 24 h post exposure (hpe), the zebrafish larvae in all groups, except for those in the control group, were treated with a 40 μM CuSO_4_ solution for 1 h. Finally, the zebrafish larvae in each group were anesthetized with tricaine (0.08%), immobilized with 3% methylcellulose, and positioned laterally on a glass slide. The macrophages were observed under a fluorescence microscope (AXIO Zoom V16, 118 ZEISS, Oberkochen, Germany), and the number of macrophages that migrated to the lateral line of the neural crest (from the cloaca to the tail) was recorded. The above experiments were performed in triplicate.

## 4. Conclusions

In this paper, we describe the discovery of six marine PIAs, including three different scaffolds. Scaffold 1 (5/6/6), which is new, displays the extraordinary rearrangement diversity of scaffolds. Moreover, discovery of the bridged scaffold, seco-spongicidin (5/7), lead us to propose a new biosynthetic pathway for latonduine PIA metabolites. Compounds **1** and **2** showed considerable anti-inflammatory effects primarily by reducing the macrophage migration induced by CuSO_4_ damage in zebrafish.

## Data Availability

Data are contained within the article or Supplementary Materials; further inquiries can be directed to the corresponding author.

## Supplementary Materials

The following supporting information can be downloaded at https://www.mdpi.com/article/10.3390/md22100477/s1. Figure S1: The voucher specimen of *Stylissa massa* in the lab; Tables S1–S5, and Figures S2–S8: Structural elucidation of compounds **1**–**6**; Figures S9 and S10: Bioactivity experimental procedures for compounds **1**–**6**; Figures S11–S54: NMR and MS spectra of compounds **1**–**6**; Tables S6 and S7: ECD calculations for compounds **1** and **2**.
