# Supplementary material for: Discovery of Anti-Inflammatory Alkaloids from Sponge Stylissa massa Suggests New Biosynthetic Pathways for Pyrrole–Imidazole Alkaloids
Source: Mar Drugs. 2024 Oct 18;22(10):477. doi: 10.3390/md22100477 (PMC11509139; doi:10.3390/md22100477)
Supplement: Supplementary file 1 [file marinedrugs-22-00477-s001.zip › marinedrugs-3213050-supplementary.pdf]

Supporting Information

Discovery of Anti-Inflammatory Alkaloids from Sponge *Stylissa massa* Suggests New Biosynthetic Pathways for Pyrrole Imidazole Alkaloids

Xiaojing Liu [1,†], Qi Wang [1,†], Yun Zhang [2,*] and Hanting Zhang [1,*]

[1] Department of Natural Medicinal Chemistry and Pharmacognosy, School of Pharmacy, Qingdao University, Qingdao 266021, People's Republic of China; liuxiaojing2@qdu.edu.cn (X.J.L.); wangqi@hmfl.ac.cn (Q.W.)
[2] Biology Institute, Qilu University of Technology (Shandong Academy of Sciences), Jinan 250103, People's Republic of China
* Correspondence: zhangyun@sdas.org (Y.Z.); htzhang@qdu.edu.cn (H.T.Z.)
† The authors contributed equally to this work.

*Corresponding author
Email: htzhang@qdu.edu.cn; zhangyun@sdas.org

Table of Contents

**1. Chemistry Experimental Procedures and Data**		
Figure S1	The voucher specimen of *Stylissa massa* in the lab	4
**2. Structural Elucidation**		
Table S1	^{1}H NMR Data for stylimassalins A-B (**1-2**), compound **3,** and **5** (*a*. acquired in DMSO-d_6. *b*. acquired in Methanol-d_4)	5
Table S2	^{13}CNMR Data for stylimassalins A-B (**1-2**), compound **3,** and **5** (*a*. acquired in DMSO-d_6. *b*. acquired in Methanol-d_4)	6
Table S3	X-ray diffraction parameter of stylimassalin B (**2**)	7
Table S4	X-ray diffraction parameter of compound **4**	8
Table S5	X-ray diffraction parameter of compound **6**	9
Figure S2	Perspective ORTEP drawing of X-ray structure of **2** (ellipsoids shown at the 30 % probability level)	10
Figure S3	Perspective ORTEP drawing of X-ray structure of **4** (ellipsoids shown at the 30 % probability level)	11
Figure S4	Perspective ORTEP drawing of X-ray structure of **6** (ellipsoids shown at the 30 % probability level)	12
Figure S5	The UV spectra of compounds **1**−**6**.	13
Figure S6	Chiral HPLC separation chromatogram of **1** on chiral Daicel Chiralpack AD-H column (250 × 4.6 mm, 5 μm). The compounds were eluted by hexane: ethanol (75:25, v:v, 0.8 mL/min).	14
Figure S7	Chiral HPLC separation chromatogram of **2** on chiral Daicel Chiralpack AD-H column (250 × 4.6 mm, 5 μm). The compounds were eluted by hexane: ethanol (80:20, v:v, 0.8 mL/min).	15
Figure S8	The absolute configurations of **1** and **2** were determined by time-dependent density functional theory ECD (TDDFT-ECD) calculations (9*S* for **1a** and **2a**; 9*R*, for **1b** and **2b**).	16
**3. Bioactivity Experimental Procedures**		
Figure S9	Analysis purity of AKR1B1 based on the SDS-PAGE result and AKTA pure system	17
Figure S10	The original data of AKR1B1 inhibitory in vitro activities measured by FlexStation 3 Reader at OD_{340} nm for compounds **1-6**.	18
**4. NMR and MS spectra of compounds 1-6**		
Figure S11	^{1}H NMR spectrum of **1** in DMSO-d_6 (500 MHz).	19
Figure S12	Close up view of ^{1}H NMR spectrum (500 MHz) of **1** in DMSO-d_6	20
Figure S13	^{13}C NMR spectrum of **1** in DMSO-d_6 (125 MHz)	21
Figure S14	^{13}C NMR and DEPT spectrum of **1** in DMSO-d_6 (125 MHz)	22
Figure S15	^{1}H-^{1}H COSY spectrum of **1** in DMSO-d_6 (500 MHz)	23
Figure S16	HSQC spectrum of **1** in DMSO-d_6 (500 MHz)	24
Figure S17	HMBC spectrum of **1** in DMSO-d_6 (500 MHz)	25
Figure S18	HRESIMS data of compound **1**	26
Figure S19	^{1}H NMR spectrum of **2** in DMSO-d_6 (500 MHz)	27
Figure S20	Close up view of ^{1}H NMR spectrum (500 MHz) of **2** in DMSO-d_6	28
Figure S21	^{13}C NMR spectrum of **2** in DMSO-d_6 (125 MHz)	29
Figure S22	^{13}C NMR and DEPT spectrum of **2** in DMSO-d_6 (125 MHz)	30

Figure S23	^{1}H-^{1}H COSY spectrum of **2** in DMSO-d_6 (500 MHz)	31
Figure S24	HSQC spectrum of **2** in DMSO-d_6 (500 MHz)	32
Figure S25	HMBC spectrum of **2** in DMSO-d_6 (500 MHz)	33
Figure S26	^{1}H NMR spectrum of compound **2** in Methanol-d_4 (600 MHz)	34
Figure S27	Close up view of ^{1}H NMR spectrum (600 MHz) of **2** in Methanol-d_4	35
Figure S28	DEPTQ spectrum (150 MHz) of **2** in Methanol-d_4	36
Figure S29	Close up view of DEPTQ spectrum (600 MHz) of **2** in Methanol-d_4	37
Figure S30	DEPTQ, DEPT135, and DEPT90 spectra (150 MHz) of **2** in Methanol-d_4	38
Figure S31	^{1}H-^{1}H COSY spectrum of **2** in Methanol-d_4 (600 MHz)	39
Figure S32	HSQC spectrum of compound **2** in Methanol-d_4 (600 MHz)	40
Figure S33	HMBC spectrum of compound **2** in Methanol-d_4 (600 MHz)	41
Figure S34	HRESIMS data of compound **2**	42
Figure S35	Close up view of HR-ESI-MS spectrum of **2**	43
Figure S36	^{1}H NMR spectrum of **3** in DMSO-d_6 (500 MHz)	44
Figure S37	^{13}C NMR spectrum of **3** in DMSO-d_6 (125 MHz)	45
Figure S38	^{13}C NMR and DEPT spectrum of **3** in DMSO-d_6 (125 MHz)	46
Figure S39	^{1}H-^{1}H COSY spectrum of **3** in DMSO-d_6 (500 MHz)	47
Figure S40	HSQC spectrum of **3** in DMSO-d_6 (500 MHz)	48
Figure S41	HMBC spectrum of **3** in DMSO-d_6 (500 MHz)	49
Figure S42	HRESIMS data of **3**	50
Figure S43	^{1}H NMR spectrum of compound **4** in DMSO-d_6 (500 MHz)	51
Figure S44	^{13}C NMR spectrum of compound **4** in DMSO-d_6 (125 MHz)	52
Figure S45	HRESIMS data of **4**	53
Figure S46	^{1}H NMR spectrum of **5** in DMSO-d_6 (500 MHz)	54
Figure S47	^{13}C NMR spectrum of **5** in DMSO-d_6 (125 MHz)	55
Figure S48	^{1}H-^{1}H COSY spectrum of **5** in DMSO-d_6 (500 MHz)	56
Figure S49	HSQC spectrum of **5** in DMSO-d_6 (500 MHz)	57
Figure S50	HMBC spectrum of **5** in DMSO-d_6 (500 MHz)	58
Figure S51	HRESIMS data of **5**	59
Figure S52	^{1}H NMR spectrum of **6** in DMSO-d_6 (500 MHz)	60
Figure S53	^{13}C NMR spectrum of **6** in DMSO-d_6 (125 MHz)	61
Figure S54	HRESIMS data of **6**	62
**5. ECD calculations**		
Table S6	Cartesian coordinates for the optimized conformers of **1a** and **1b**	63
Table S7	Cartesian coordinates for the optimized conformers of **2a** and **2b**	66

1. Chemistry Experimental Procedures and Data

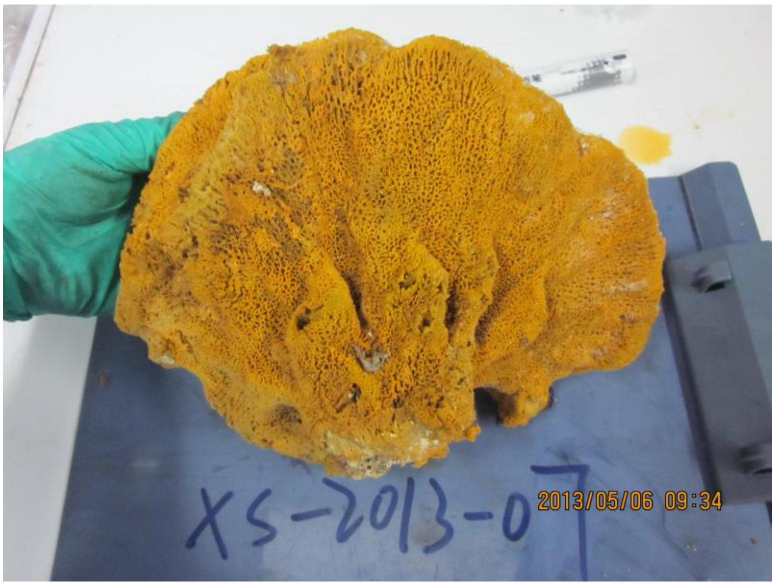

Figure S1. The voucher specimen of *Stylissa massa* in the lab.

2.Structural Elucidation

Table S1. ¹H NMR Data for stylimassalins A-B (**1-2**), compound **3,** and **5** (*a*. acquired in DMSO-d_6. *b*. acquired in Methanol-d_4).

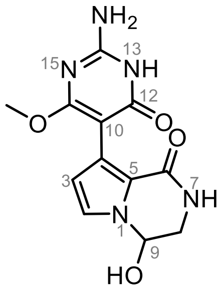

1

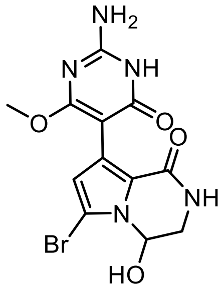

2

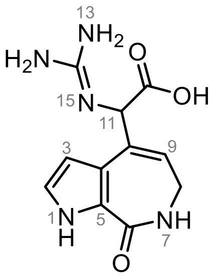

3

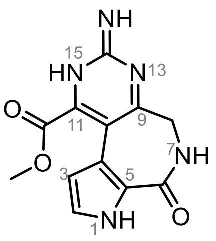

5

no.	¹H NMR (*J*, Hz)				
	**1**[a]	**2**[a]	**2**[b]	**3**[a]	**5**[a]
1				11.89, brs	11.90, brs
2	6.93, d (2.6)			7.02, t (2.8)	7.01, t (2.8)
3	6.02, d (2.6)	6.11, s	6.24, s	6.30, t (2.8)	6.04, t (2.8)
4					
5					
6					
7	7.38, brs	7.38, brs		7.69, t (5.3)	8.01, t (5.3)
8	3.54, dd (13.0, 2.1);<br>3.29, ddd (13.0, 7.5, 2.5)	3.61, dd (13.0,1.5);<br>3.30, dd (13.0, 7.5)	3.79, ddd (13.0, 7.5, 2.5)<br>3.56, dd (13.0, 1.5);	3.42, m	3.94, d (5.3)
9	5.54, dd (2.5, 1.5)	5.58, brs	5.75, dd (2.5, 1.5)	5.98, t (6.7)	
10					
11				5.24, d (8.6)	
12					
13					
14					
15					
16					
17					
18					
19					
20					
21					
11-OMe	3.65, s	3.64, s	3.79, s		
12-OMe					3.80, s

Table S2. ^{13}CNMR Data for stylimassalins A-B (**1-2**), compound **3,** and **5** (*a*. acquired in DMSO-d_6. *b*. acquired in Methanol-d_4).

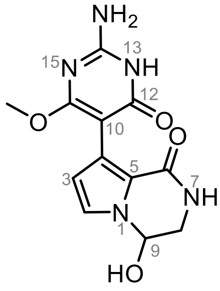

1

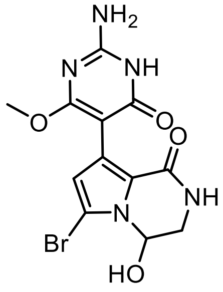

2

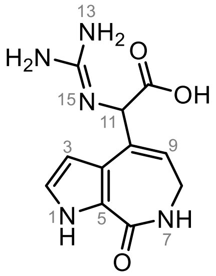

3

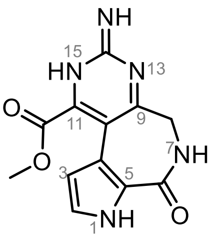

5

no.	^{13}C NMR				
	**1**^{a}	**2**^{a}	**2**^{b}	**3**^{a}	**5**^{a}
1					
2	120.2, CH	101.9, qC	105.3, qC	122.1, CH	122.6, CH
3	112.3, CH	114.8, CH	116.9, CH	106.5, CH	106.3, CH
4	unobserved	unobserved	124.1, qC	121.9, qC	118.7, qC
5	119.8, qC	120.7, qC	123.0, qC	126.2, qC	123.7, qC
6	119.3, qC	158.2, qC	161.8, qC	163.2, qC	166.0, qC
7					
8	46.3, CH_2	46.4, CH_2	47.9, CH_2	37.6, CH_2	47.0, CH_2
9	74.4, CH	72.5, CH	74.4, CH	123.7, CH	111.3, qC
10	89.5, CH	unobserved	90.4, qC	134.5, qC	163.3, qC
11	167.0, qC	166.9, qC	170.3, qC	57.4, CH	161.1, qC
12	159.4, qC	158.2, qC	163.3, qC	170.7, qC	166.8, qC
13					
14	153.7, qC	153.8, qC	155.7, qC	156.4, qC	156.3, qC
15					
16					
17					
18					
19					
20					
21					
11-OMe	53.3, CH_3	53.2, CH_3	54.5, CH_3		
12-OMe					52.5, CH_3

Table S3. X-ray diffraction parameter of stylimassalin B (**2**).

Identification code	compound 2
Empirical formula	$C_{13}H_{17}BrN_5O_5$
Formula weight	402.22
Temperature/K	150.00
Crystal system	triclinic
Space group	P-1
a/Å	7.89330(10)
b/Å	10.9192(2)
c/Å	10.9199(2)
α/°	93.0740(10)
β/°	104.4540(10)
γ/°	106.5560(10)
Volume/Å^3	865.67(3)
Z	2
ρ_{calc}g/cm^3	1.543
μ/mm^{-1}	3.550
F(000)	408.0
Crystal size/mm^3	0.2 × 0.15 × 0.1
Radiation	CuKα (λ = 1.54178)
2Θ range for data collection/°	8.438 to 149.474
Index ranges	-9 ≤ h ≤ 9, -13 ≤ k ≤ 13, -13 ≤ l ≤ 13
Reflections collected	20018
Independent reflections	3537 [R_{int} = 0.0409, R_{sigma} = 0.0320]
Data/restraints/parameters	3537/0/220
Goodness-of-fit on F^2	1.089
Final R indexes [I>=2σ (I)]	R_1 = 0.0475, wR_2 = 0.1371
Final R indexes [all data]	R_1 = 0.0488, wR_2 = 0.1381
Largest diff. peak/hole / e Å^{-3}	1.36/-0.71

Table S4. X-ray diffraction parameter of compound **4**.

Empirical formula	$C_{13}H_{18}BrN_5O_4$
Formula weight	388.23
Temperature/K	298
Crystal system	monoclinic
Space group	P21/n
a/Å	9.0283(3)
b/Å	15.5478(3)
c/Å	11.7424(3)
α/°	90
β/°	90.939(2)
γ/°	90
Volume/Å^3	1648.06(8)
Z	4
ρ_{calc}g/cm^3	1.565
μ/mm^{-1}	3.656
F(000)	792.0
Crystal size/mm^3	0.2 × 0.1 × 0.1
Radiation	CuKα (λ = 1.54184)
2Θ range for data collection/°	9.44 to 152.61
Index ranges	-11 ≤ h ≤ 9, -19 ≤ k ≤ 18, -14 ≤ l ≤ 14
Reflections collected	11402
Independent reflections	3322 [Rint = 0.0858, Rsigma = 0.0906]
Data/restraints/parameters	3322/0/210
Goodness-of-fit on F^2	1.077
Final R indexes [I>=2σ (I)]	R1 = 0.0556, wR2 = 0.1518
Final R indexes [all data]	R1 = 0.0886, wR2 = 0.1736
Largest diff. peak/hole / e Å^{-3}	0.55/-0.79

Table S5. X-ray diffraction parameter of compound **6**.

Identification code	4672-WQ-SM-5662-100K_autored
Empirical formula	$C_{12}H_{10}BrN_5O_3$
Formula weight	350.99
Temperature/K	99.98(10)
Crystal system	triclinic
Space group	P-1
a/Å	8.8006(4)
b/Å	11.8337(6)
c/Å	16.1447(6)
α/°	69.871(4)
β/°	82.147(3)
γ/°	81.776(4)
Volume/Å^3	1555.51(13)
Z	2
ρ_{calc}g/cm^3	1.688
μ/mm^{-1}	3.939
F(000)	800.0
Crystal size/mm^3	0.2 × 0.15 × 0.1
Radiation	CuKα (λ = 1.54184)
2Θ range for data collection/°	5.856 to 132.014
Index ranges	$-10 \leq h \leq 10$, $-14 \leq k \leq 14$, $-19 \leq l \leq 14$
Reflections collected	15099
Independent reflections	5402 [Rint = 0.0395, Rsigma = 0.0374]
Data/restraints/parameters	5402/80/488
Goodness-of-fit on F^2	1.060
Final R indexes [I>=2σ (I)]	R1 = 0.0584, wR2 = 0.1612
Final R indexes [all data]	R1 = 0.0615, wR2 = 0.1643
Largest diff. peak/hole / e Å^{-3}	1.90/-0.60

Figure S2. Perspective ORTEP drawing of X-ray structure of **2** (ellipsoids shown at the 30 % probability level).

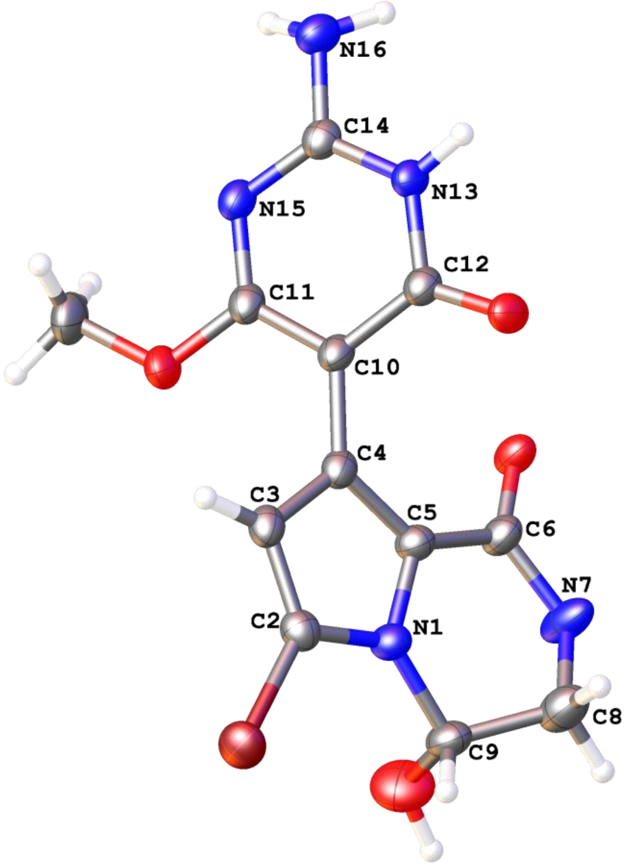

Figure S3. Perspective ORTEP drawing of X-ray structure of **4** (ellipsoids shown at the 30 % probability level).

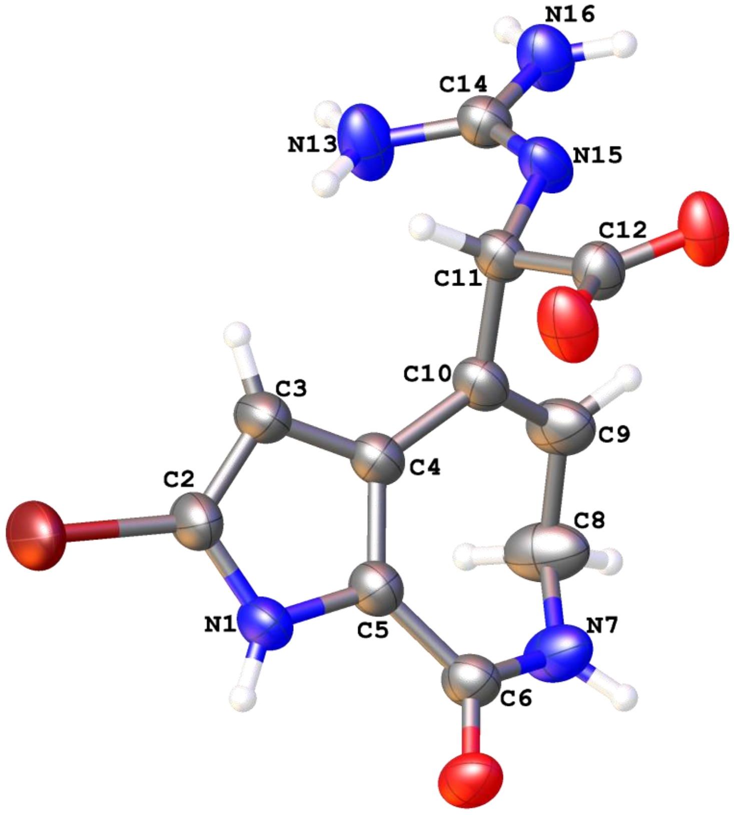

Figure S4. Perspective ORTEP drawing of X-ray structure of **6** (ellipsoids shown at the 30 % probability level).

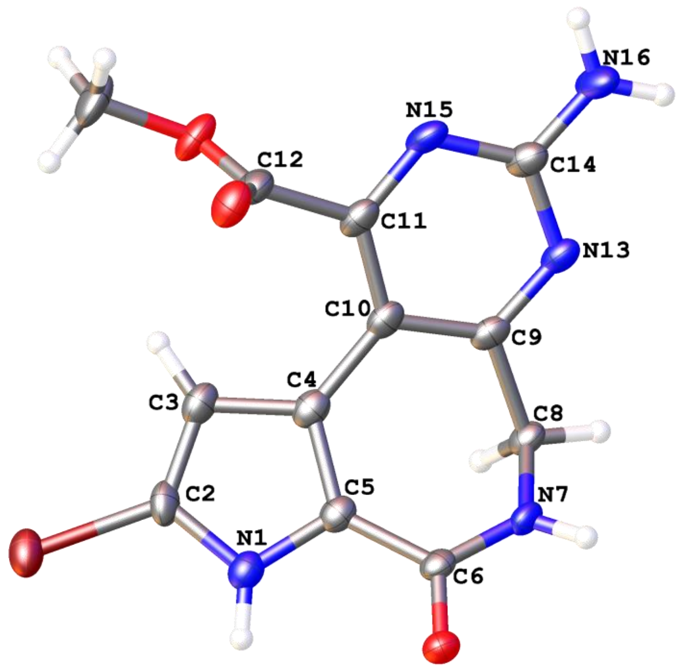

Figure S5. The UV spectra of compounds **1**−**6**.

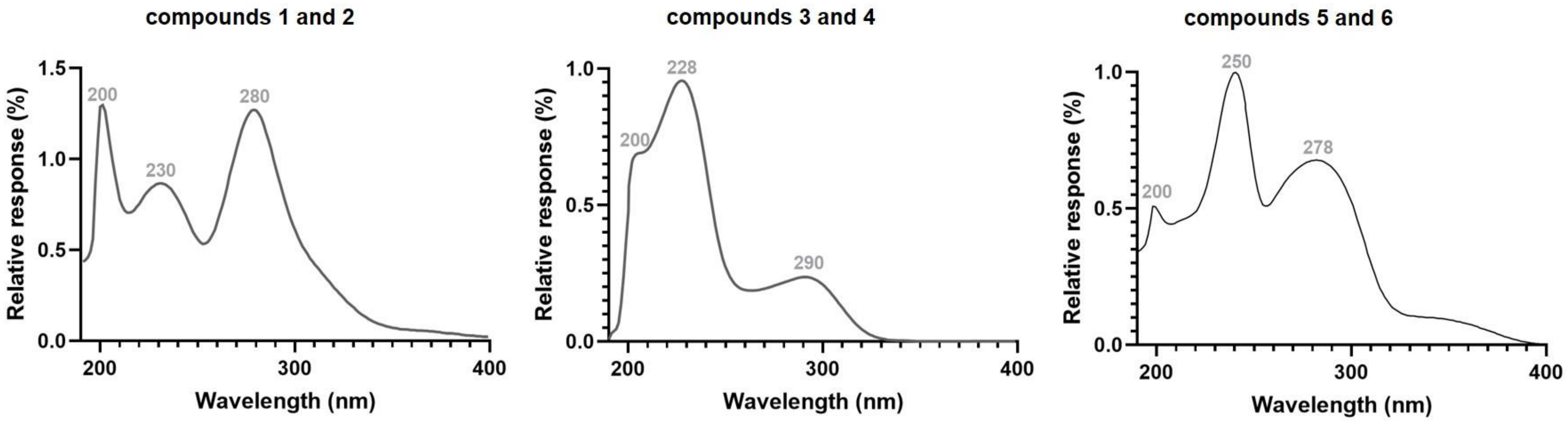

Figure S6. Chiral HPLC separation chromatogram of **1** on chiral Daicel Chiralpack AD-H column (250 × 4.6 mm, 5 μm). The compounds were eluted by hexane: ethanol (75:25, v:v, 0.8 mL/min).

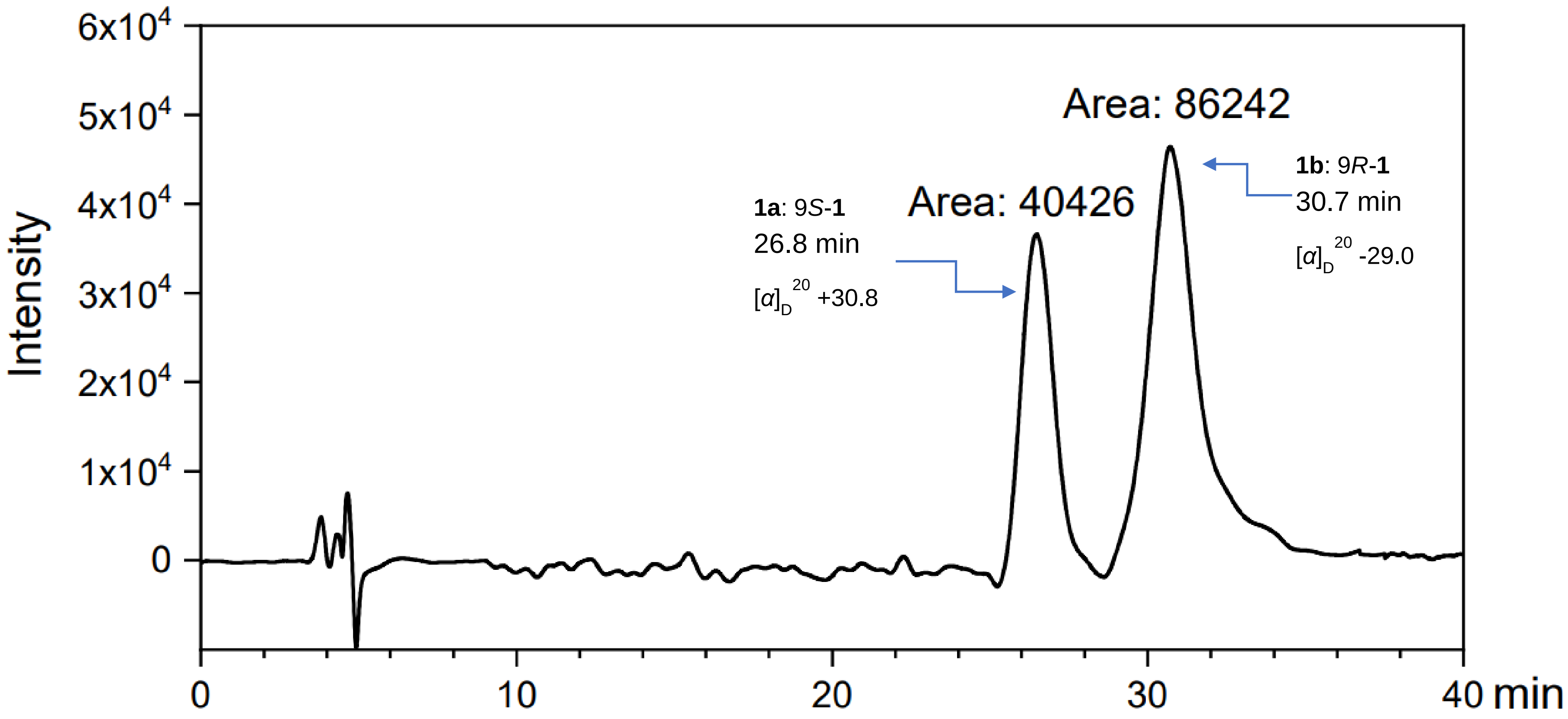

Figure S7. Chiral HPLC separation chromatogram of **2** on chiral Daicel Chiralpack AD-H column (250 × 4.6 mm, 5 μm). The compounds were eluted by hexane: ethanol (80:20, v:v, 0.8 mL/min).

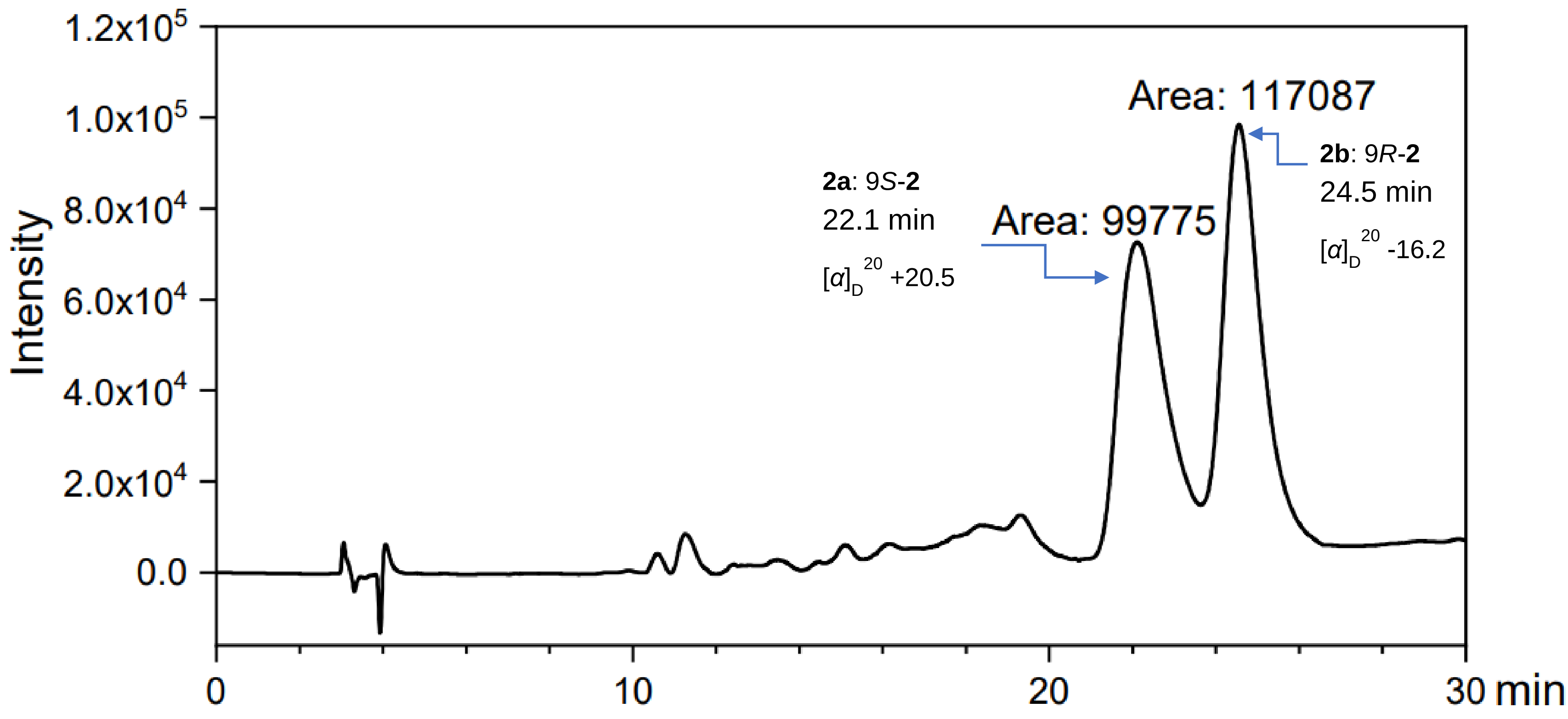

Figure S8. The absolute configurations of **1** and **2** were determined by time-dependent density functional theory ECD (TDDFT-ECD) calculations (9*S* for **1a** and **2a**; 9*R*, for **1b** and **2b**).

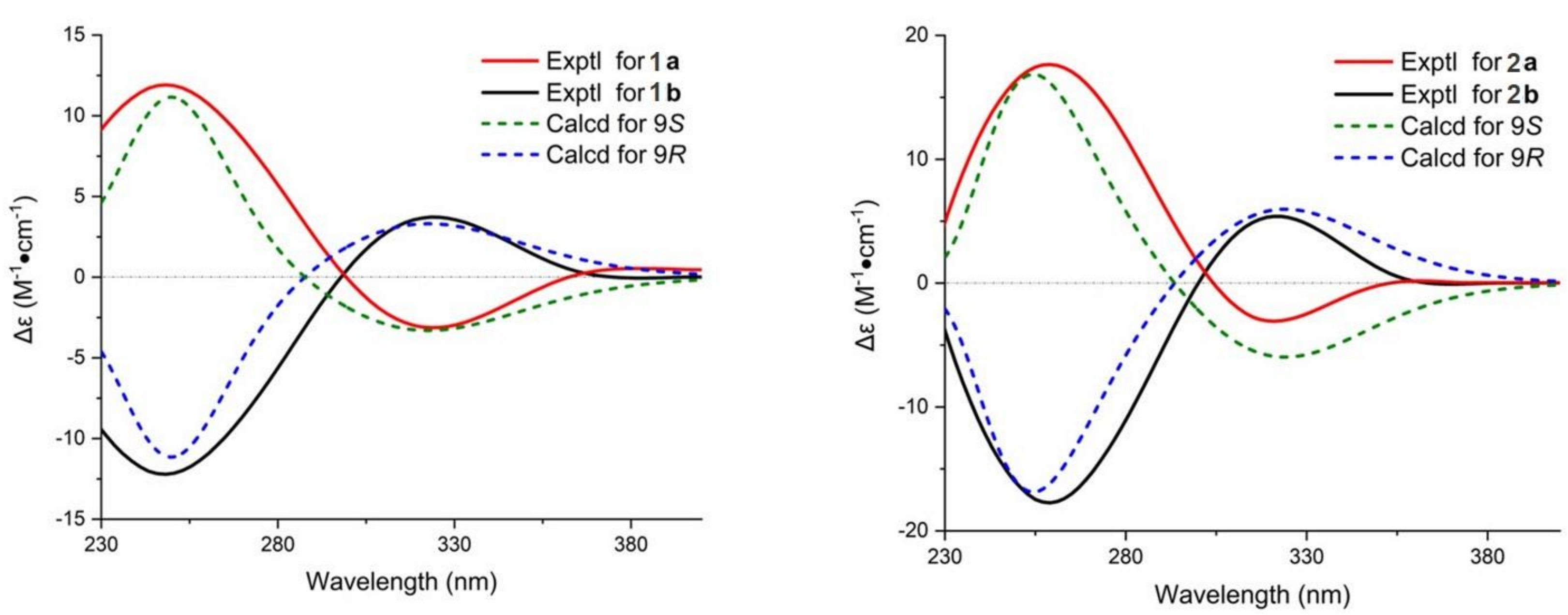

3. Bioactivity Experimental Procedures

Figure S9. Analysis purity of AKR1B1 based on the SDS-PAGE result and AKTA pure system.

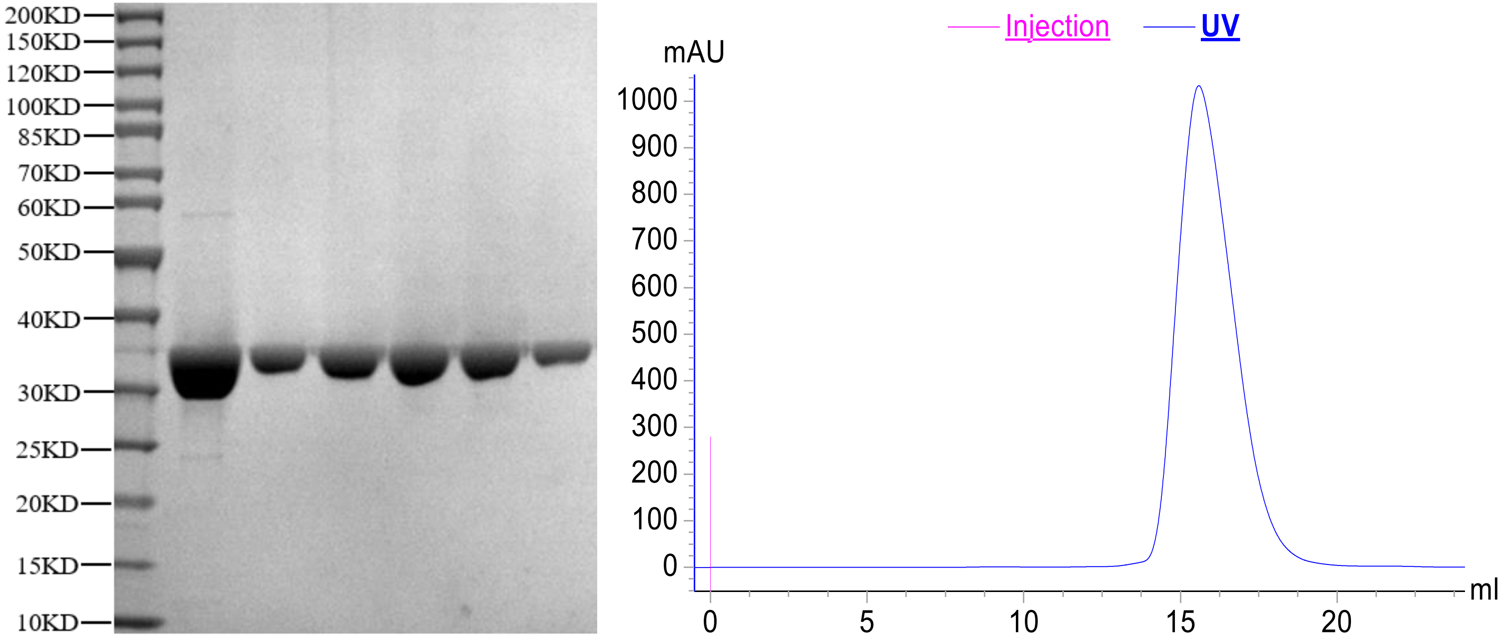

Figure S10. The original data of AKR1B1 inhibitory in vitro activities measured by FlexStation 3 Reader at OD_{340} nm for compounds **1-6**.

Plate#1

	OD_1	OD_2	positive control				compound 1	2	3	4	5	6
	1	2	3	4	5	6	7	8	9	10	11	12
A	0.250	0.213	0.226	0.222	0.213	0.225	0.215	0.211	0.211	0.215	0.211	0.207
B	0.246	0.213	0.228	0.217	0.218	0.225	0.213	0.215	0.210	0.215	0.208	0.204
C	0.248	0.211	0.227	0.215	0.212	0.222	0.209	0.211	0.208	0.217	0.208	0.206
D	0.049	0.049	0.051	0.041	0.399	0.044	0.853	0.133	0.046	0.395	0.347	0.043
E	0.050	0.047	0.043	0.049	0.559	0.041	0.055	0.072	0.043	0.048	0.048	0.047
F	0.049	0.047	0.427	0.462	0.050	0.608	0.049	0.047	0.218	0.044	0.048	0.044
G	0.244	0.244	0.242	0.238	0.041	0.041	0.041	0.042	0.043	0.042	0.043	0.041
H	0.043	0.043	0.043	0.047	0.043	0.045	0.042	0.041	0.041	0.042	0.044	0.042

Wavelength Combination: !Lm1
Mean Temperature: 24.9
Data Type: Absorbance
Reader: Flexstation III ROM v3.0.22 16Feb11

4. NMR and MS spectra of compounds 1-6.

Figure S11. ^{1}H NMR spectrum (500 MHz) of **1** in DMSO-d_6.

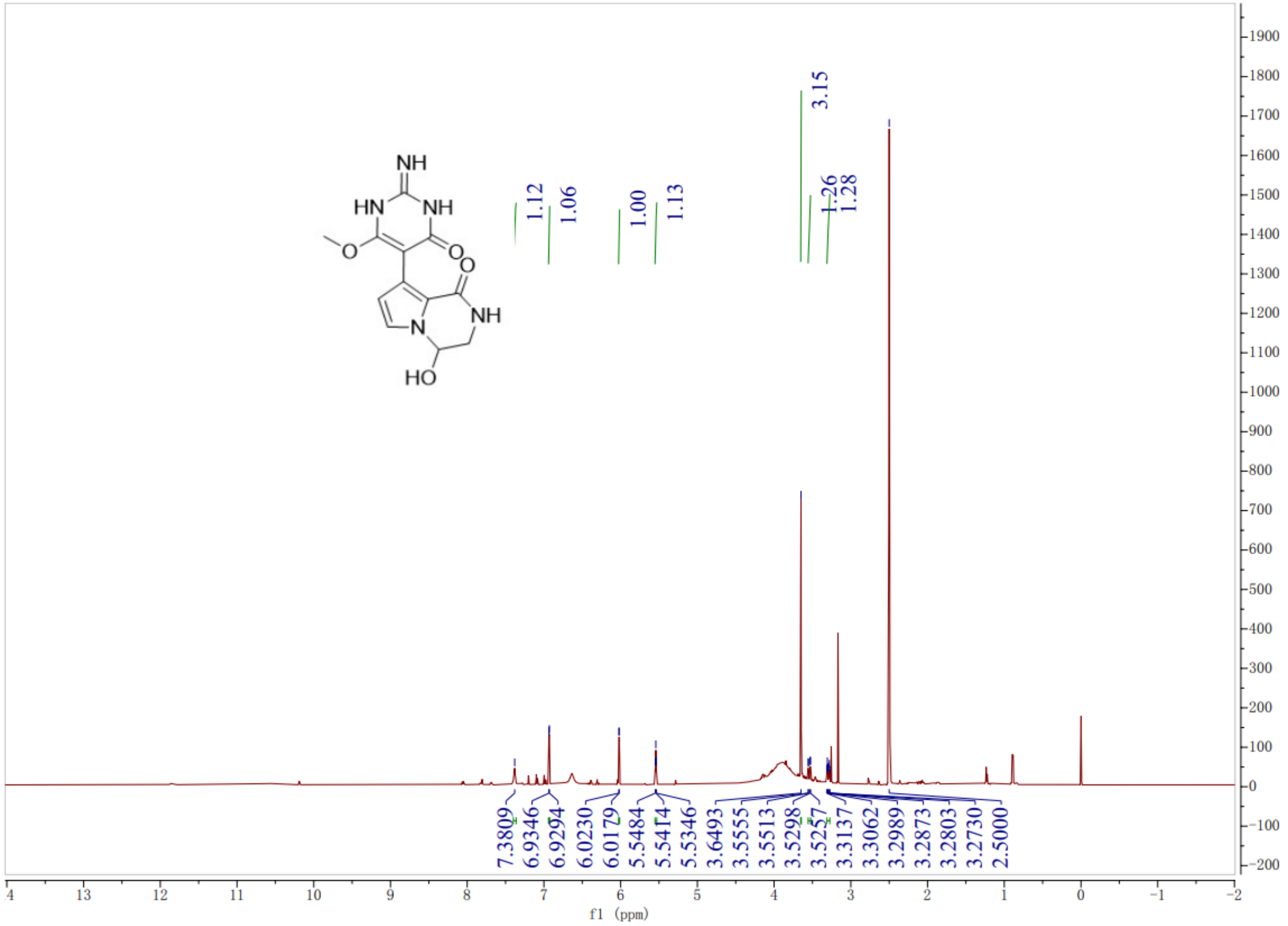

Figure S12. Close up view of ^{1}H NMR spectrum (500 MHz) of **1** in DMSO-d_6.

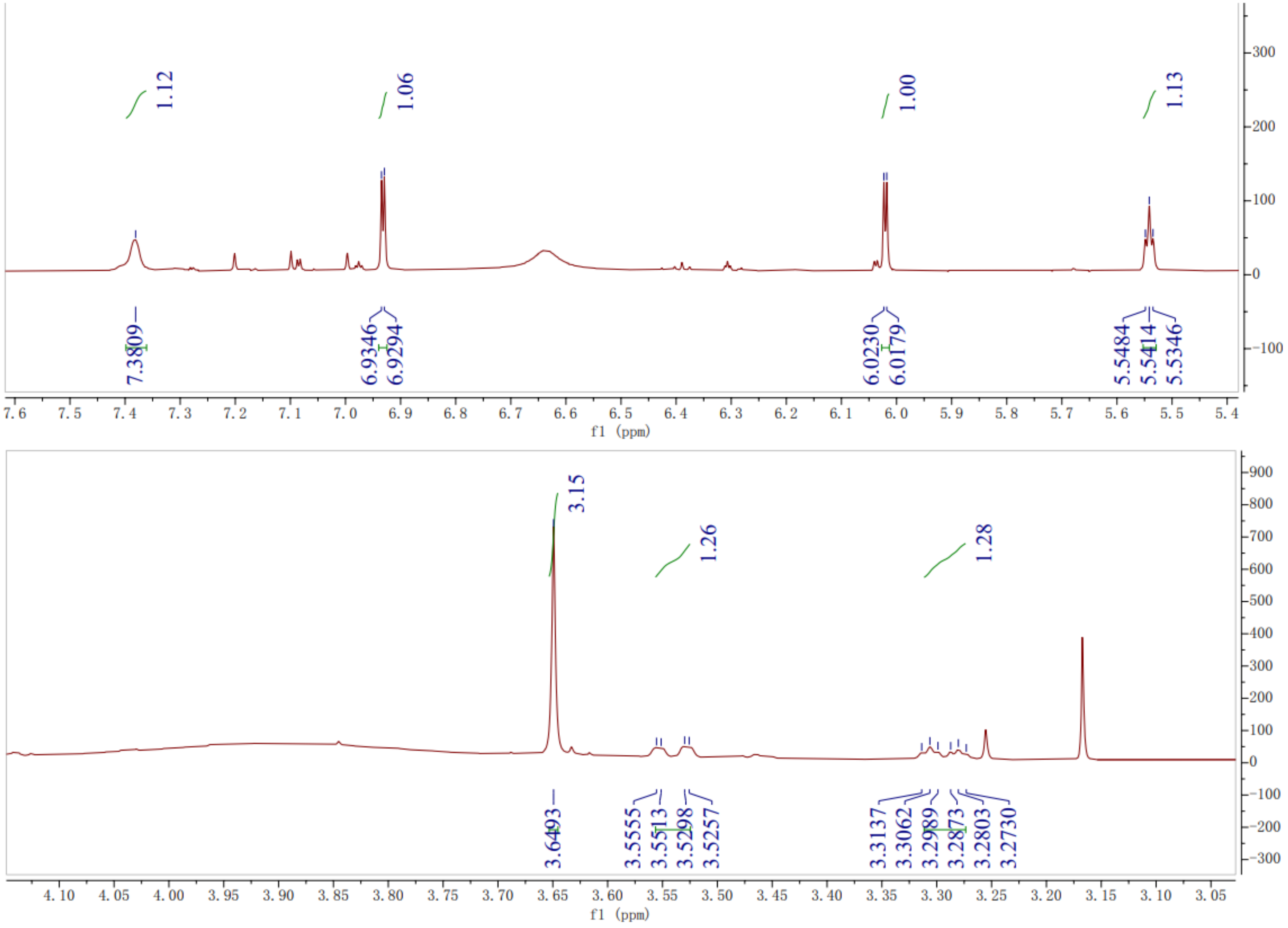

Figure S13. ^{13}C NMR spectrum (125 MHz) of **1** in DMSO-d_6.

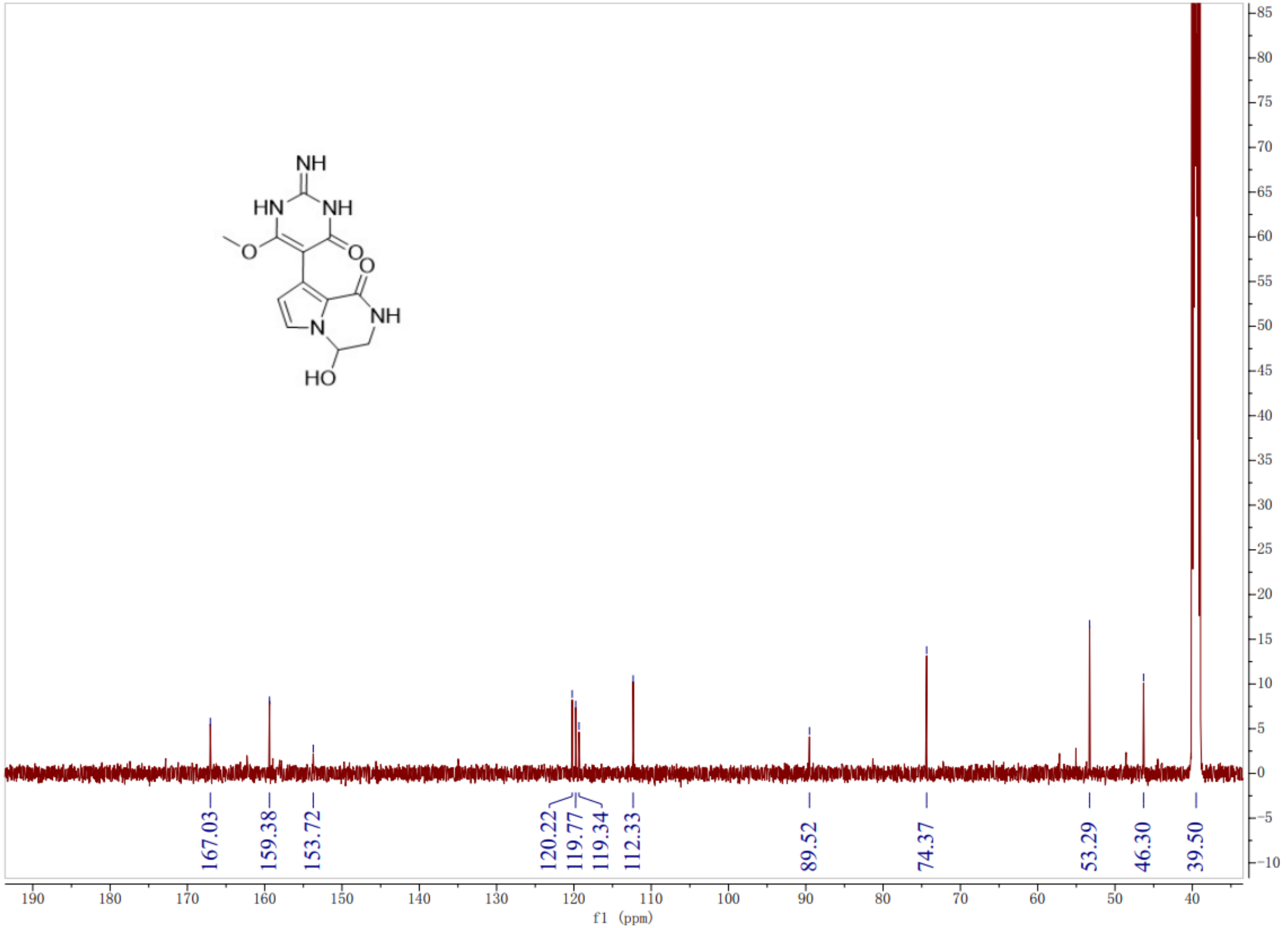

Figure S14. ^{13}C NMR and DEPT spectra (125 MHz) of **1** in DMSO-d_6.

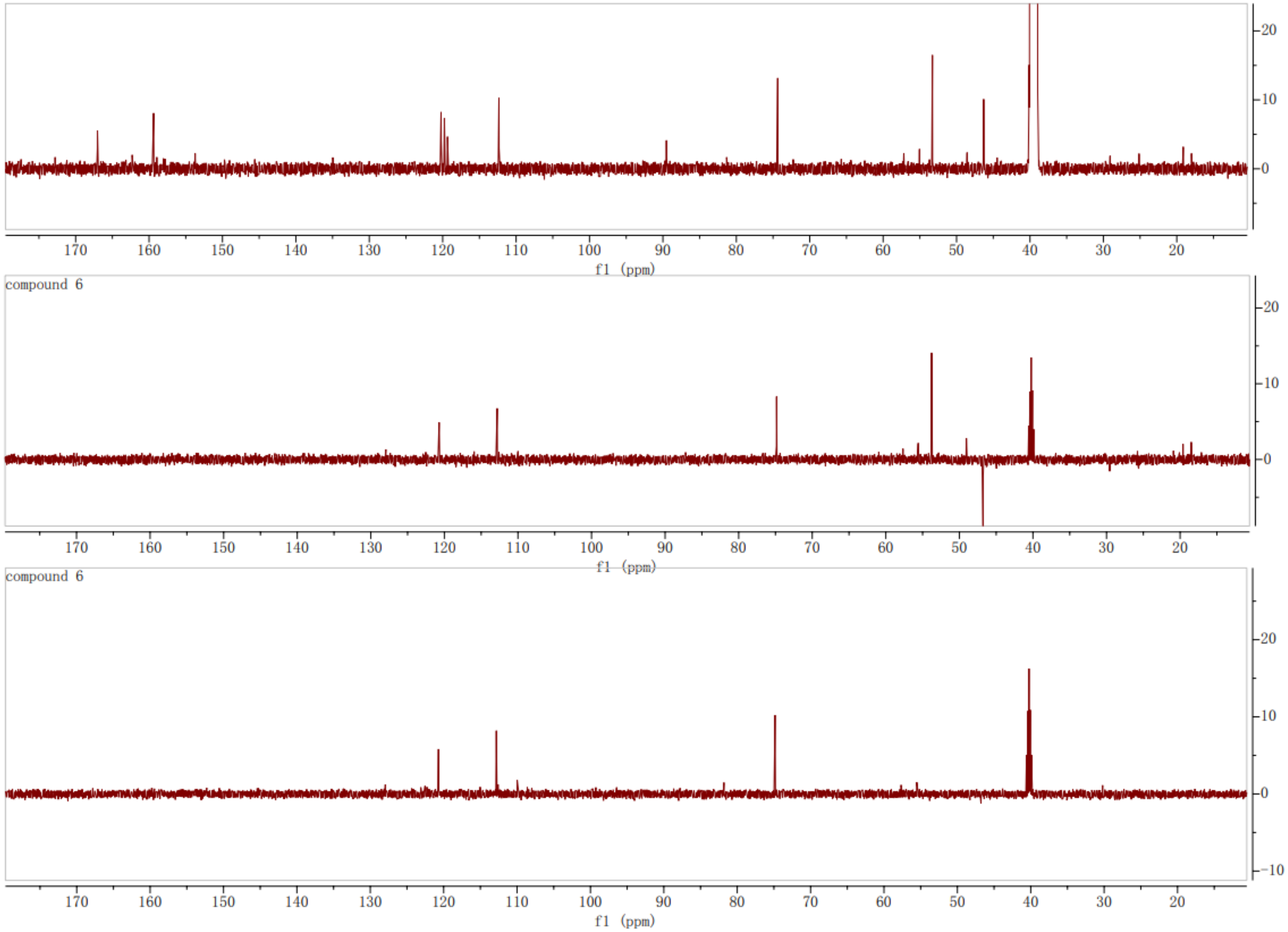

Figure S15. ^{1}H-^{1}H COSY spectrum (500 MHz) of **1** in DMSO-d_6.

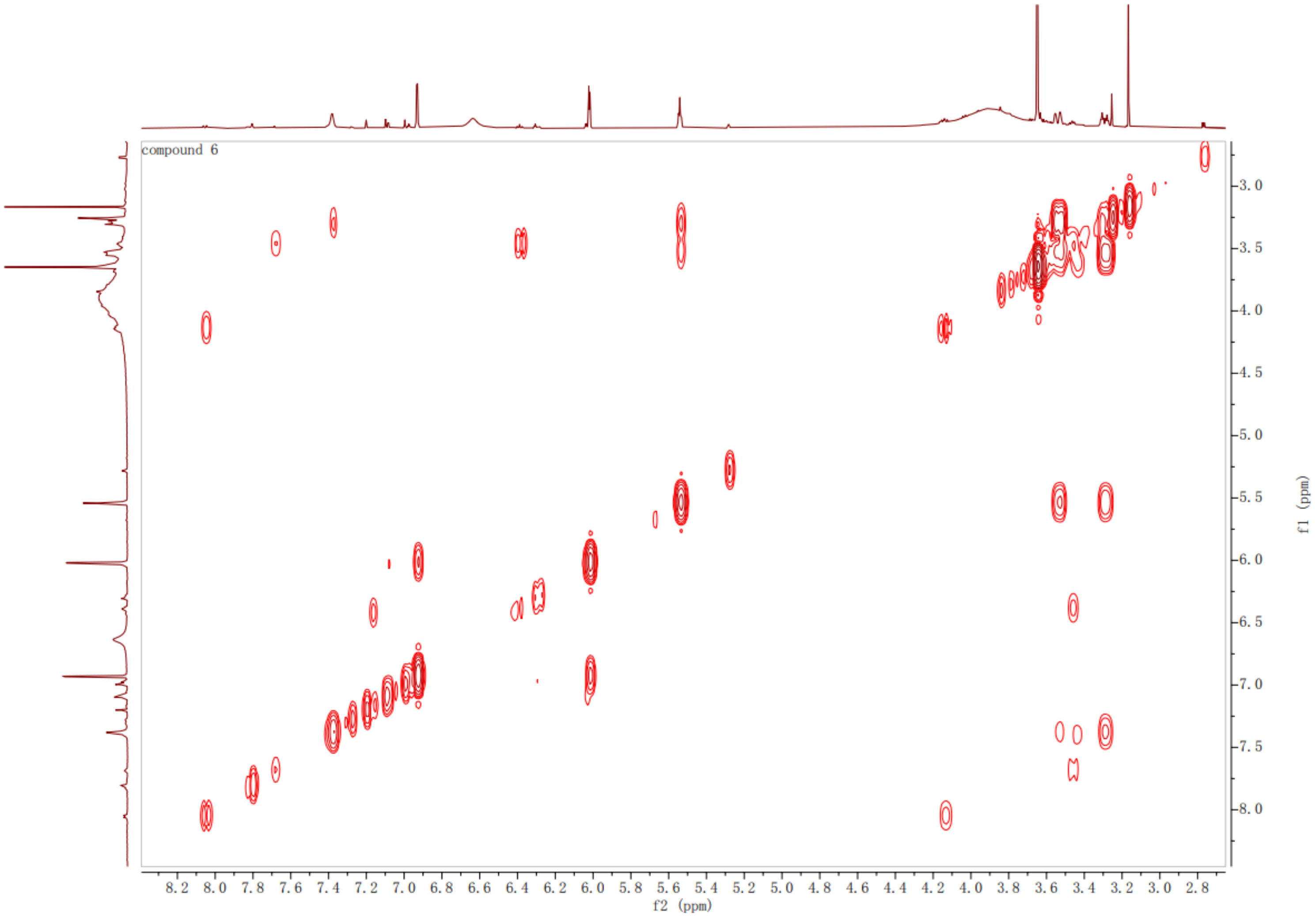

Figure S16. HSQC spectrum (500 MHz) of **1** in DMSO-d_6.

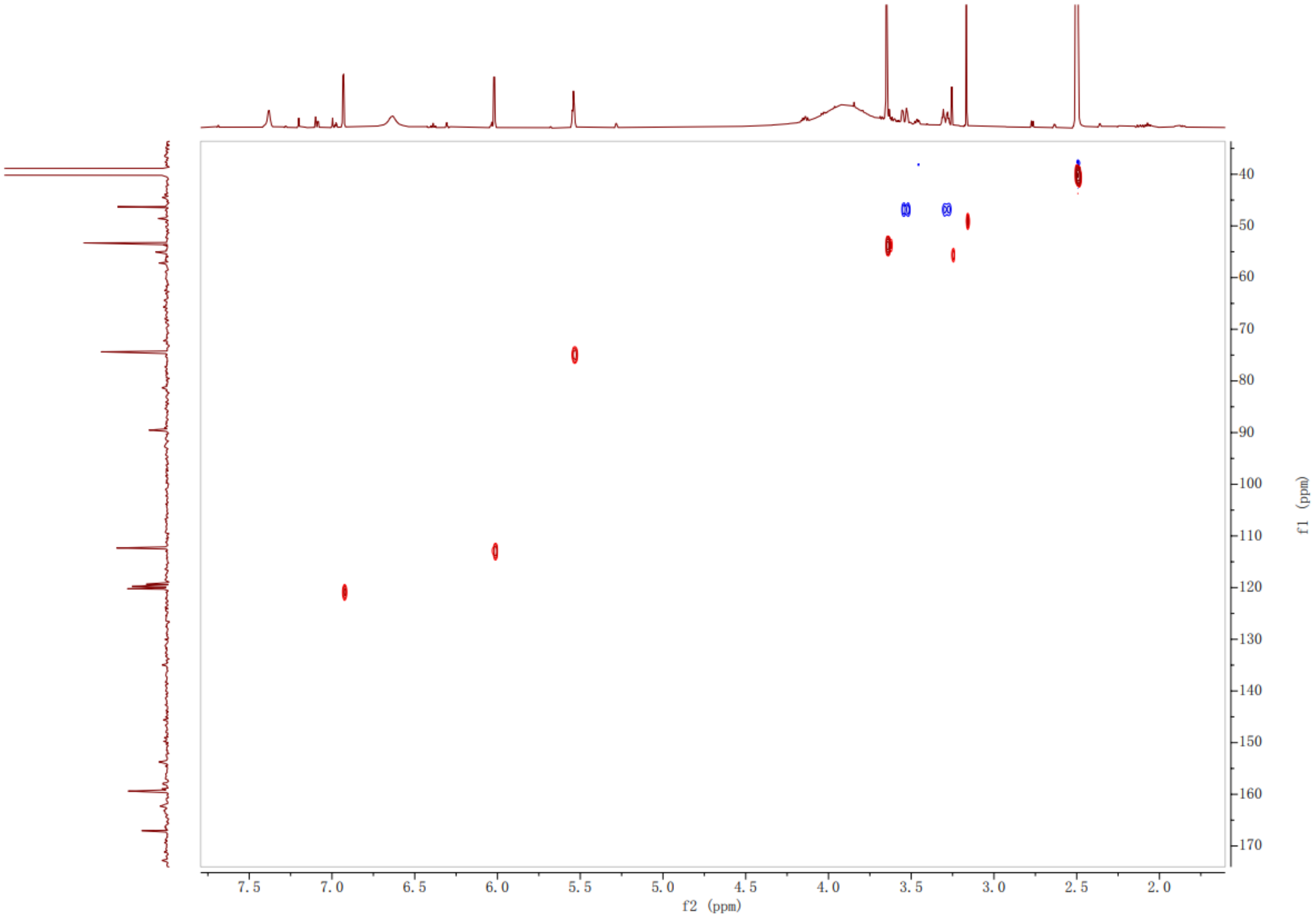

Figure S17. HMBC spectrum (500 MHz) of **1** in DMSO-d_6.

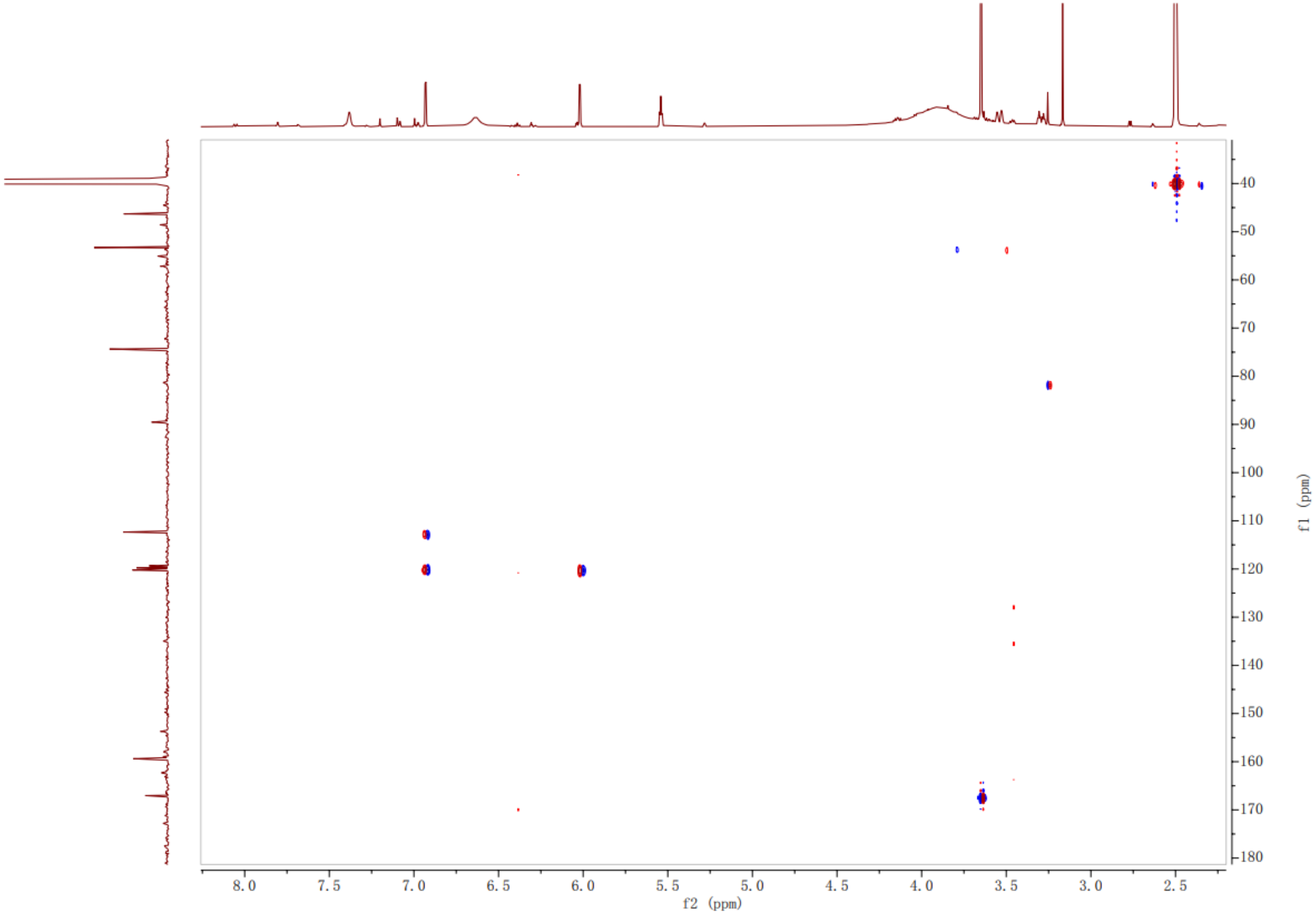

Figure S18. HR-ESI-MS spectrum of **1**.

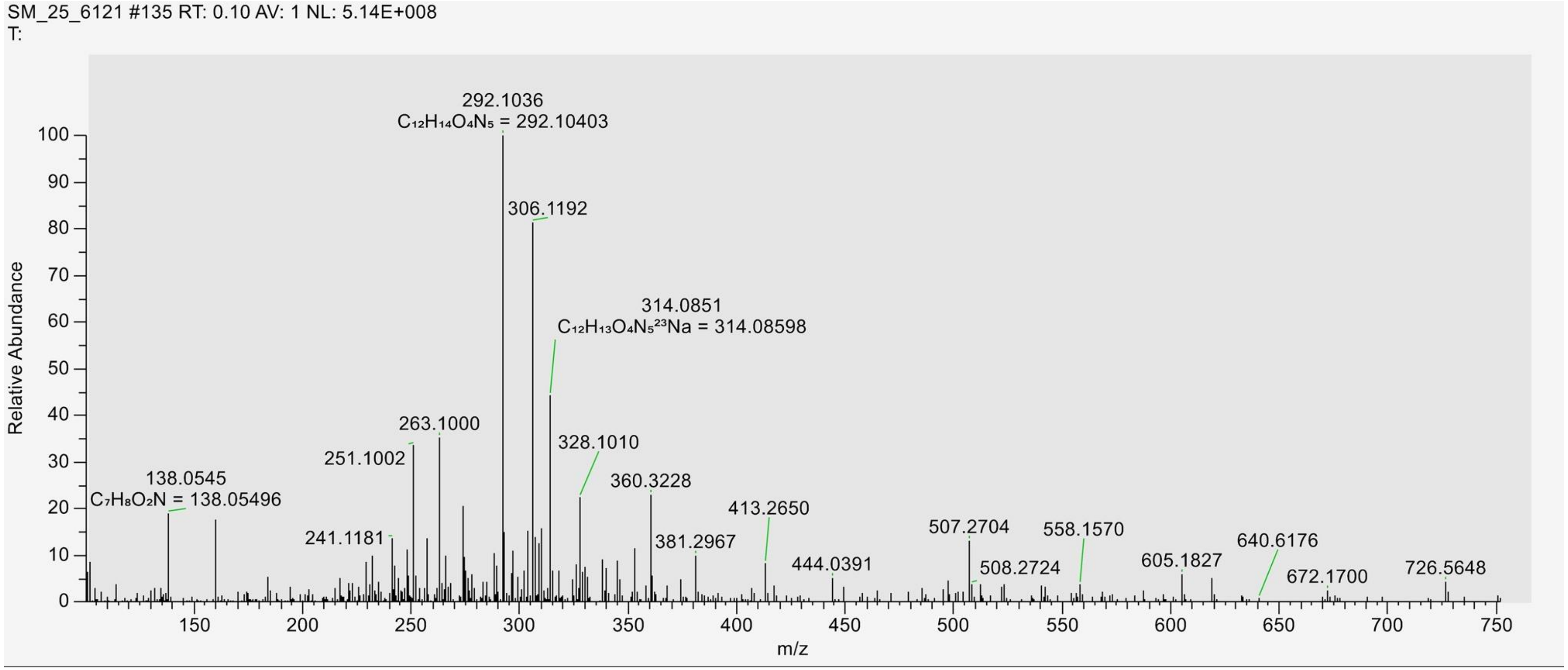

Figure S19. ^{1}H NMR spectrum (500 MHz) of **2** in DMSO-d_6.

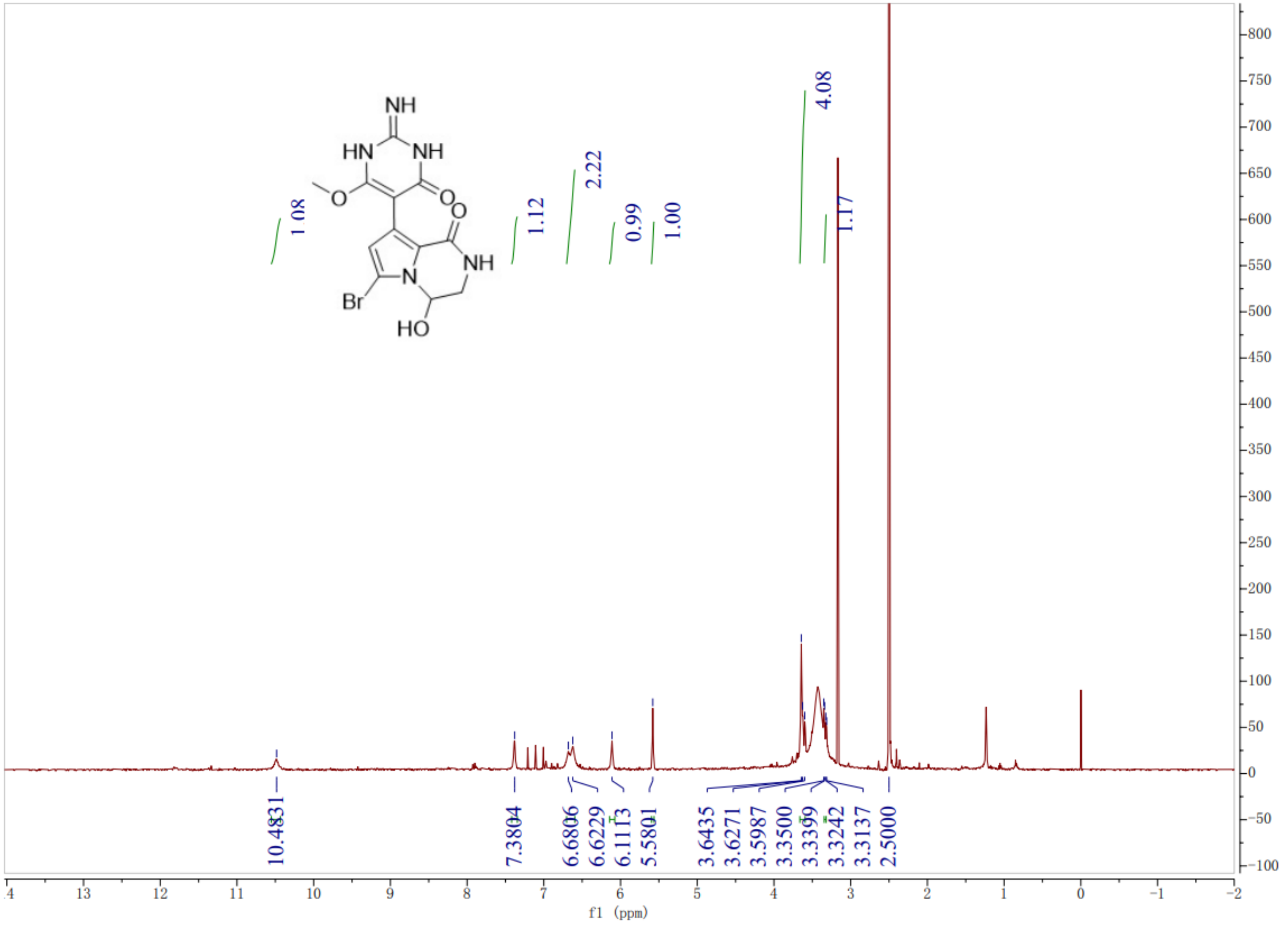

Figure S20. Close up view of ^{1}H NMR spectrum (500 MHz) of **2** in DMSO-d_6.

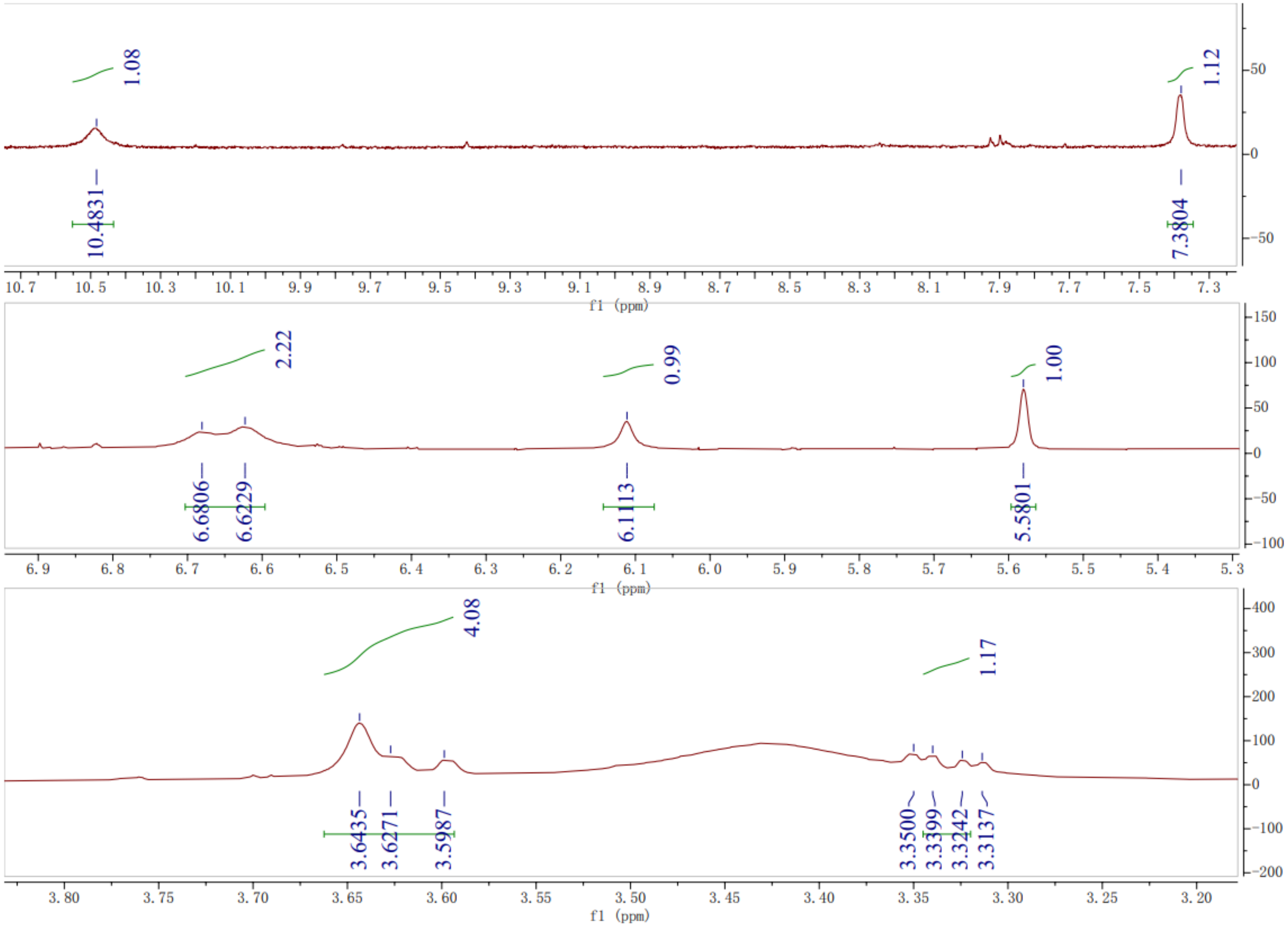

Figure S21. ^{13}C NMR spectrum (125 MHz) of **2** in DMSO-d_6.

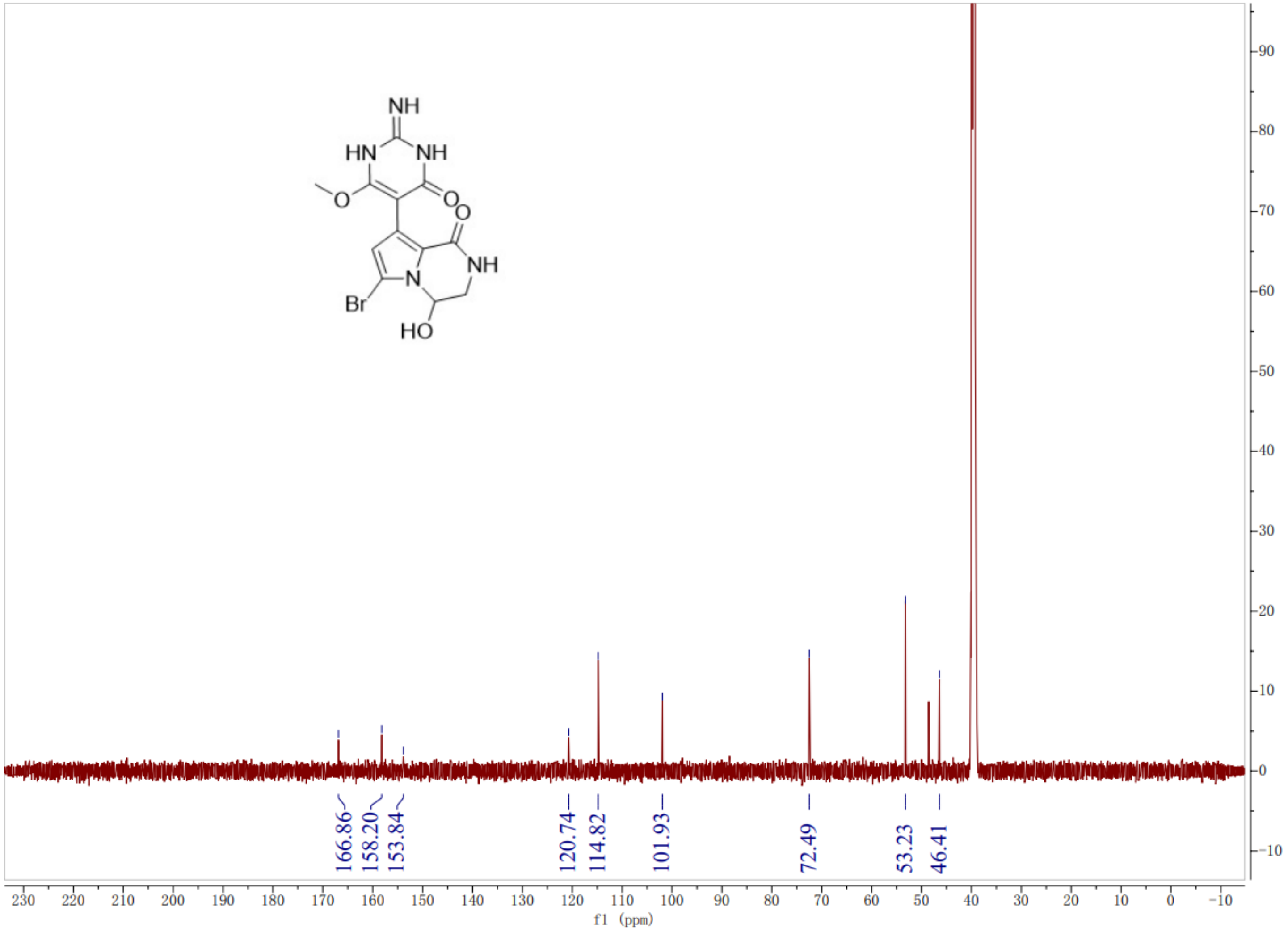

Figure S22. ^{13}C NMR and DEPT spectra (125 MHz) of **2** in DMSO-d_6.

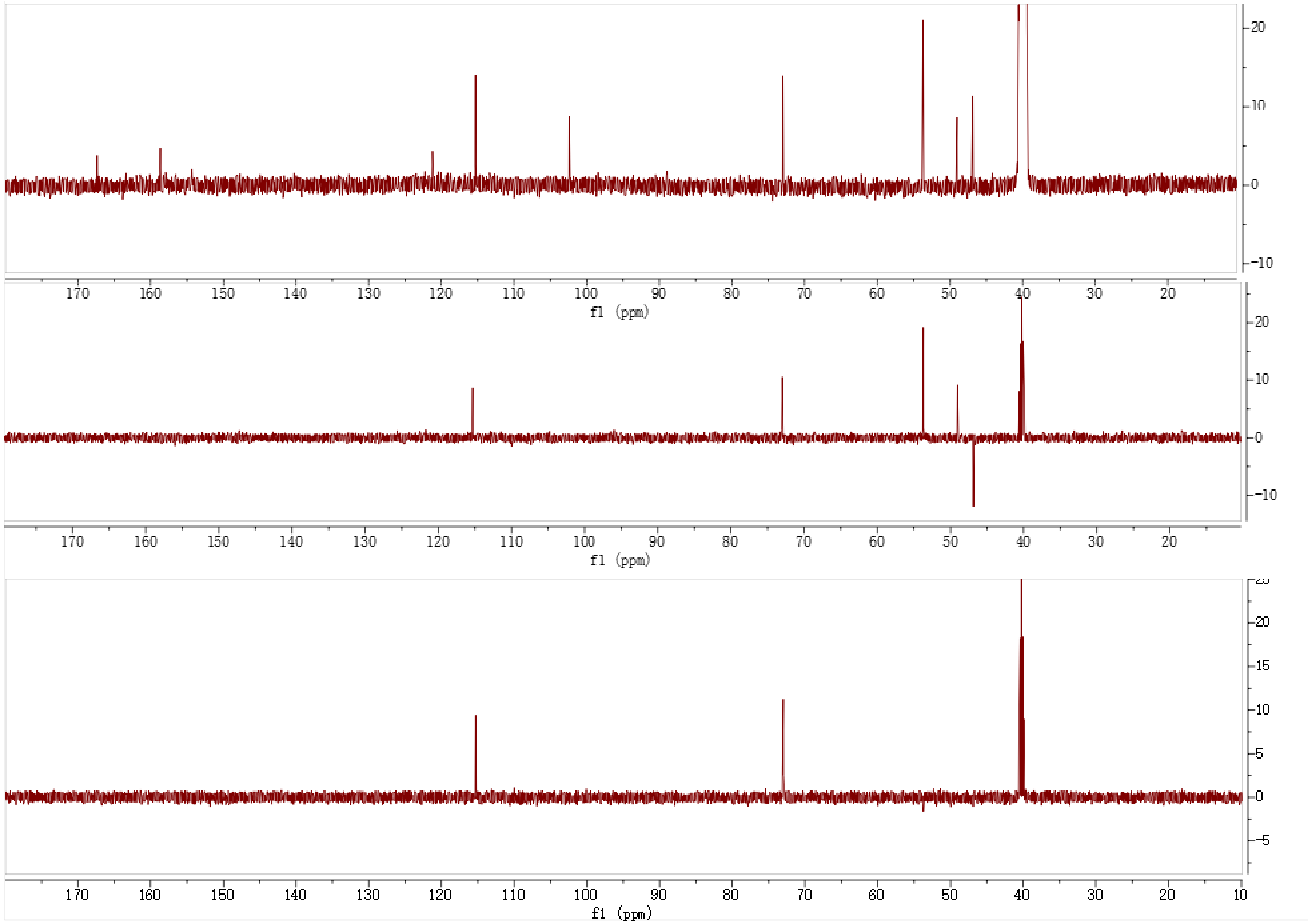

Figure S23. ^1H-^1H COSY spectrum (500 MHz) of **2** in DMSO-d_6.

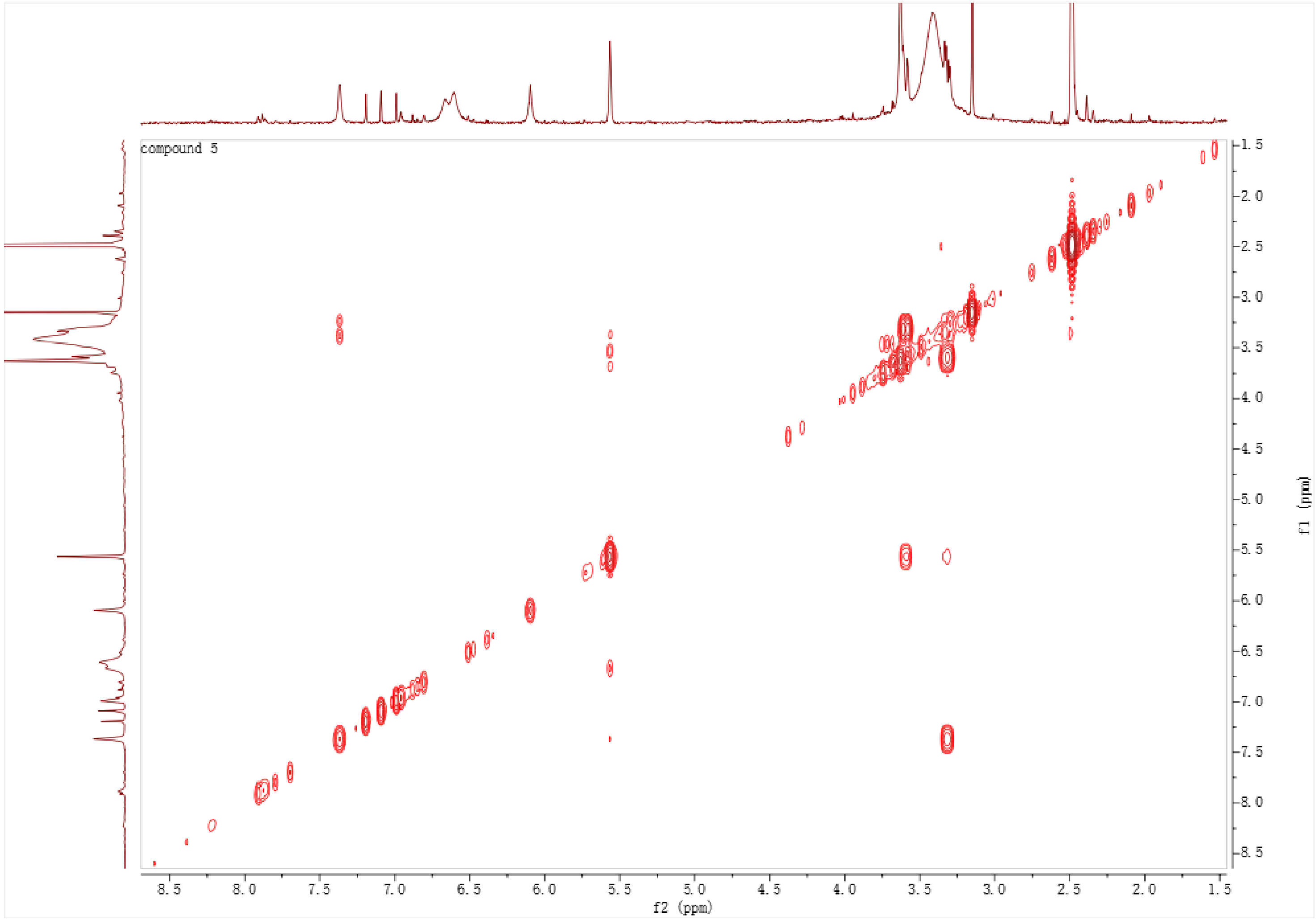

Figure S24. HSQC spectrum (500 MHz) of **2** in DMSO-d_6.

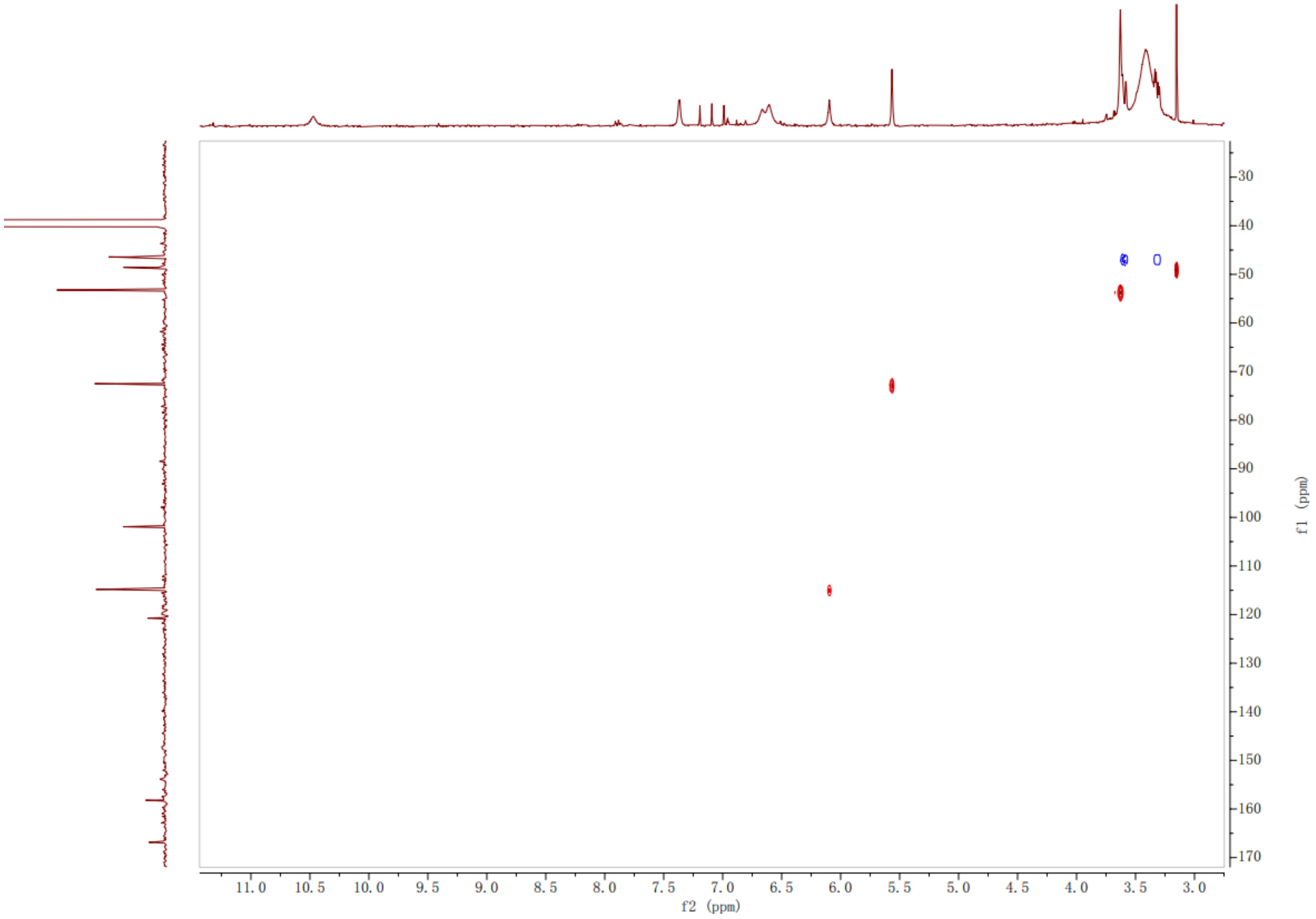

Figure S25. HMBC spectrum (500 MHz) of **2** in DMSO-d_6.

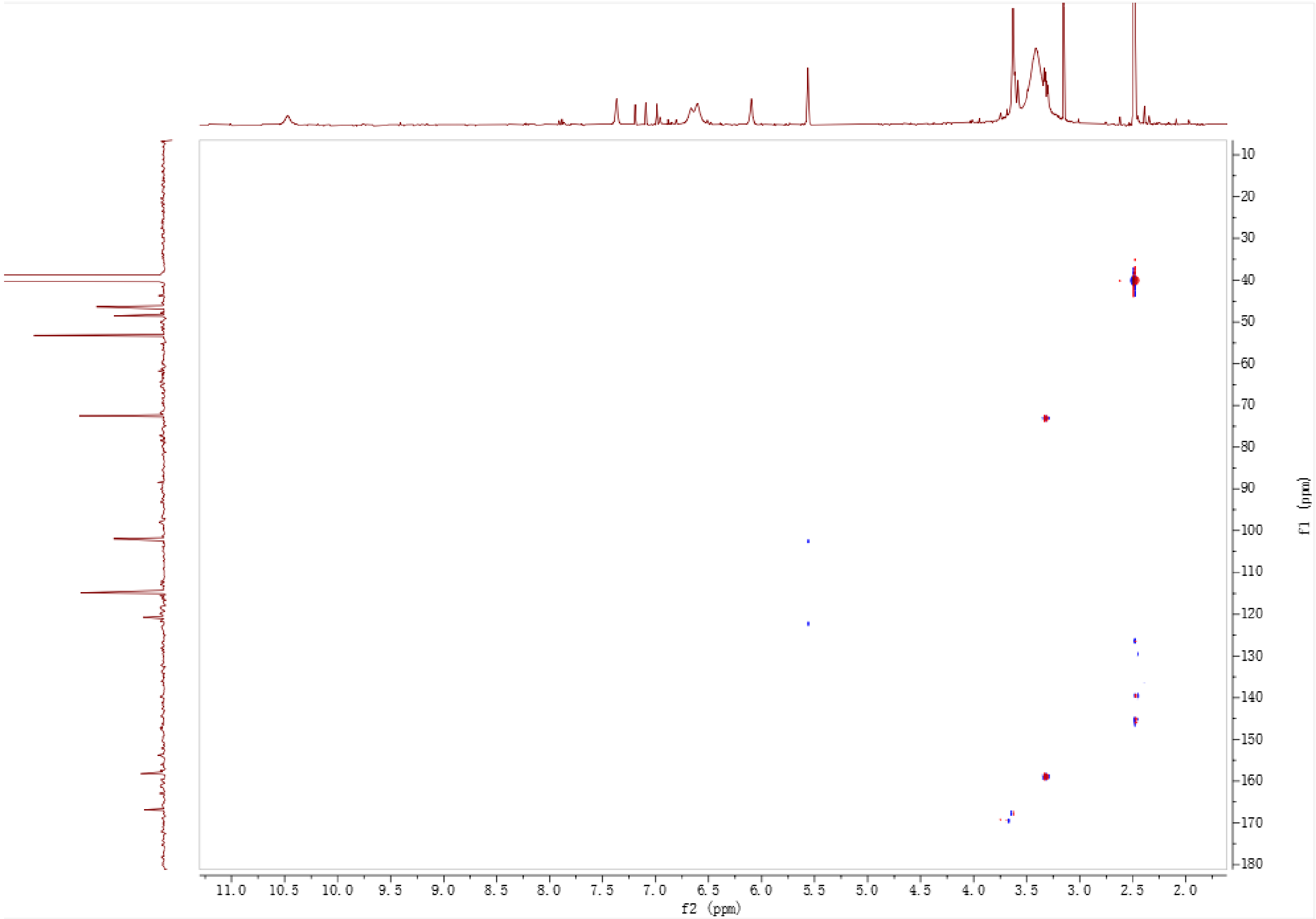

Figure S26. ^{1}H NMR spectrum (600 MHz) of **2** in Methanol-d_4.

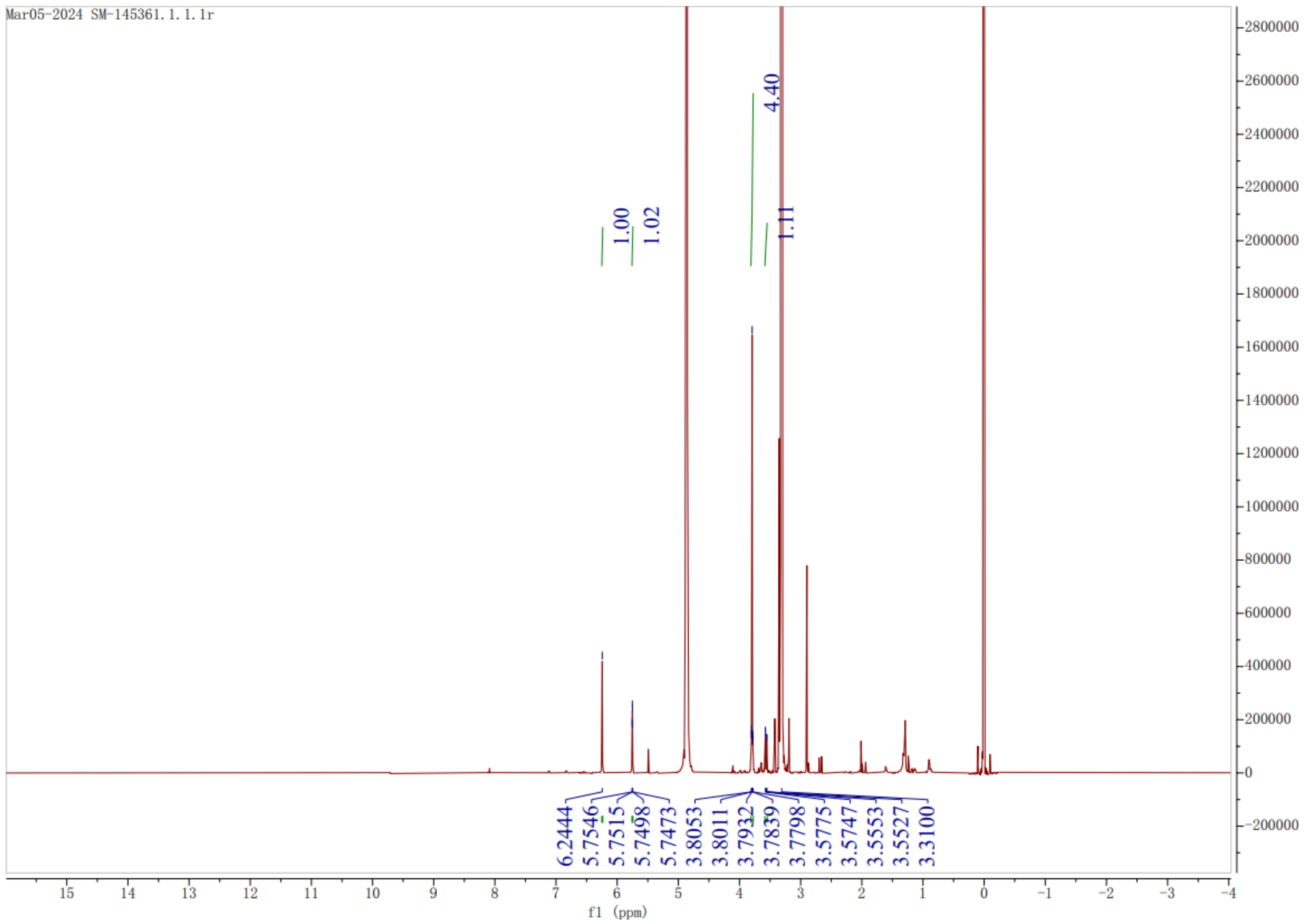

Figure S27. Close up view of ^{1}H NMR spectrum (600 MHz) of **2** in Methanol-d_4.

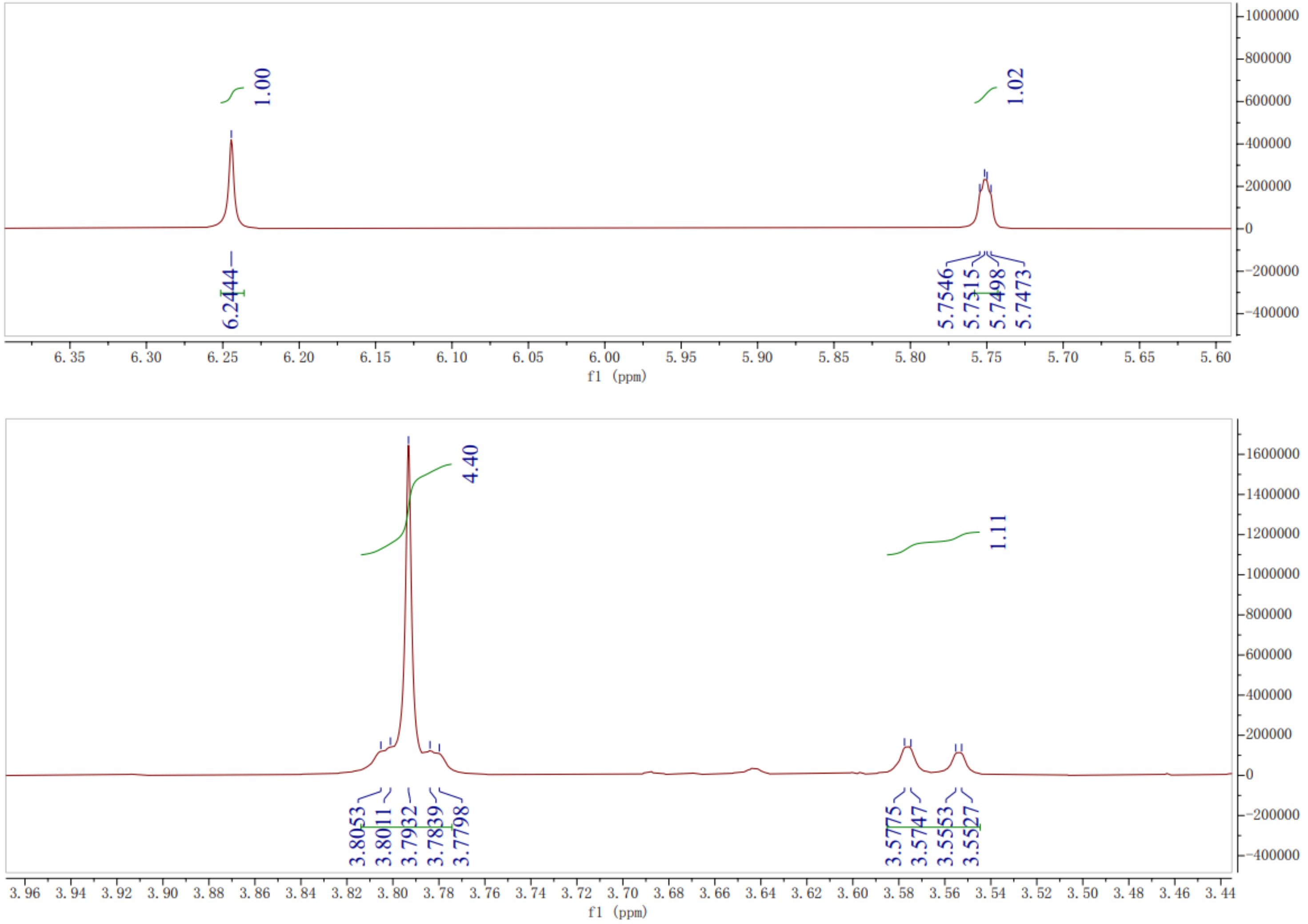

Figure S28. DEPTQ spectrum (150 MHz) of **2** in Methanol-d_4.

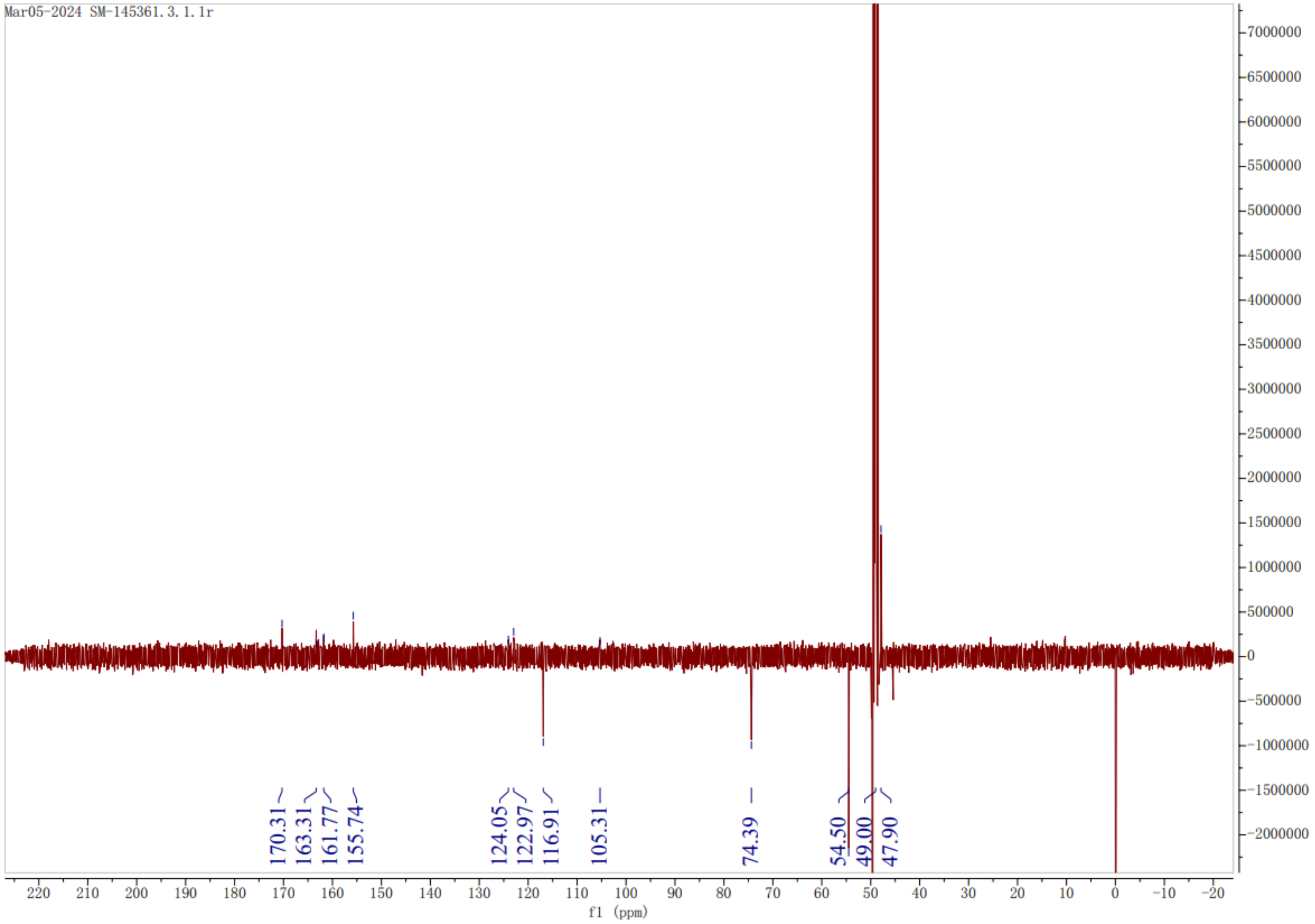

Figure S29. Close up view of DEPTQ spectrum (600 MHz) of **2** in Methanol-d_4.

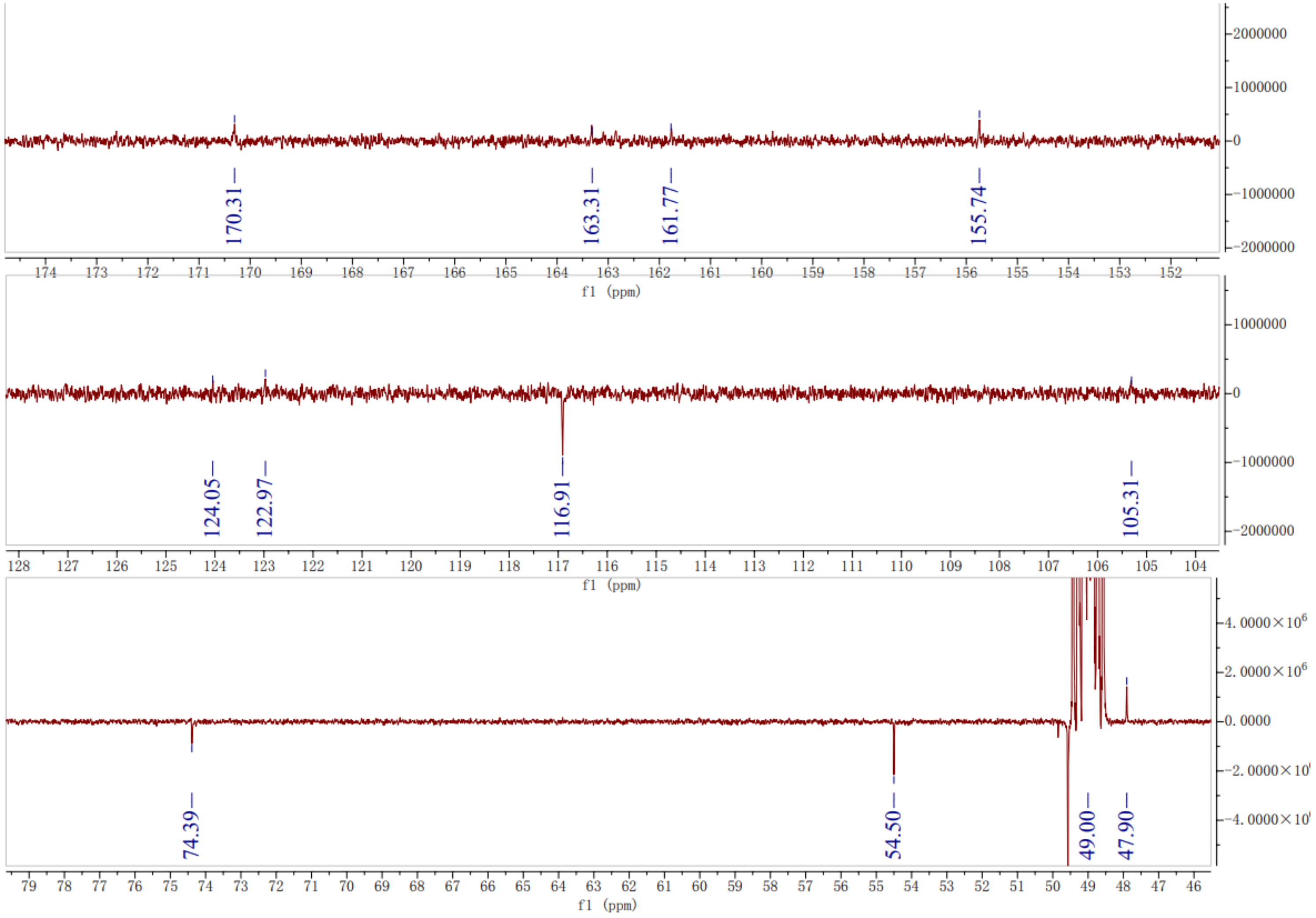

Figure S30. DEPTQ, DEPT135, and DEPT90 spectra (150 MHz) of **2** in Methanol-d_4.

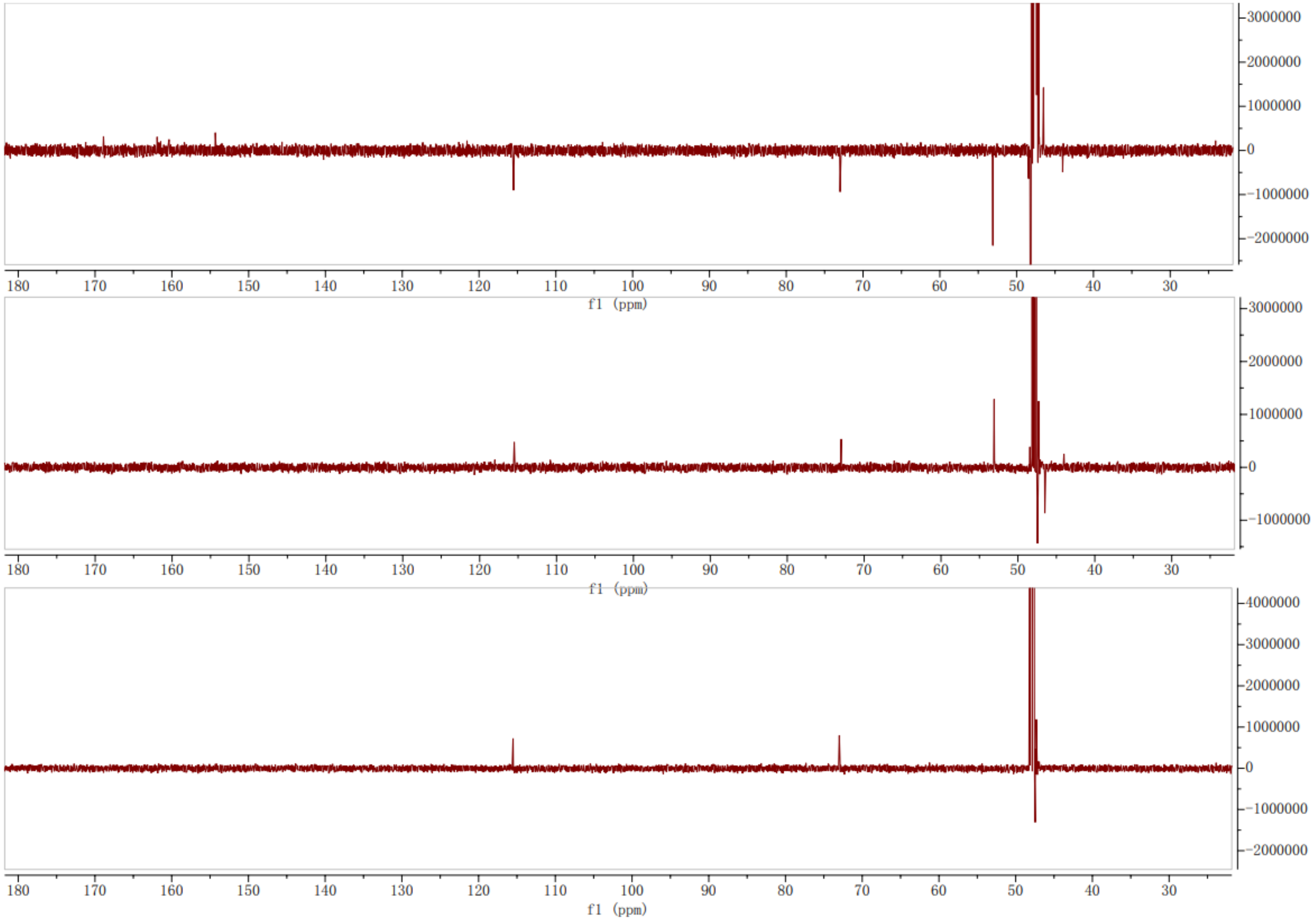

Figure S31. ^{1}H-^{1}H COSY spectrum (600 MHz) of **2** in Methanol-d_4.

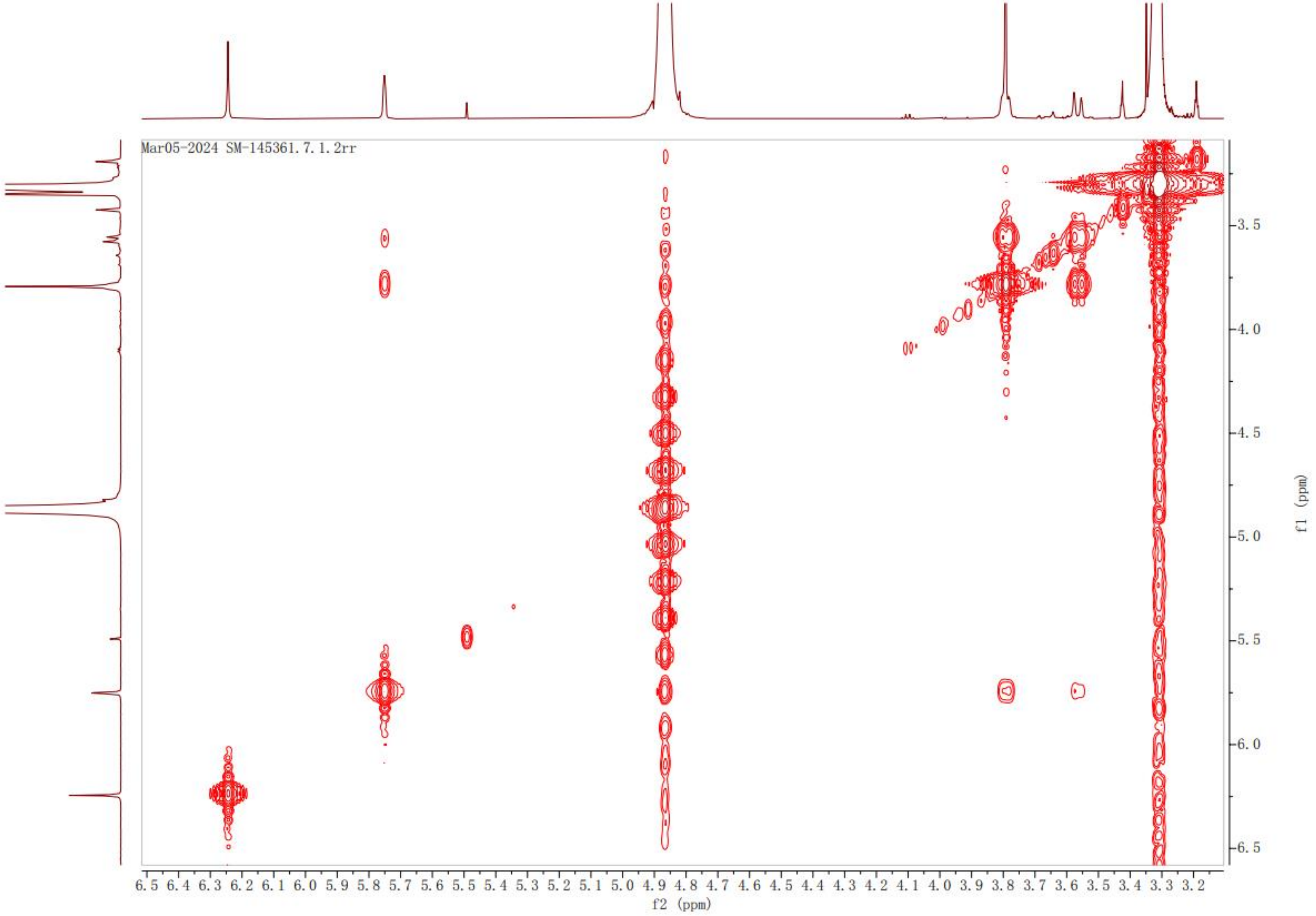

Figure S32. HSQC spectrum (600 MHz) of **2** in Methanol-d_4.

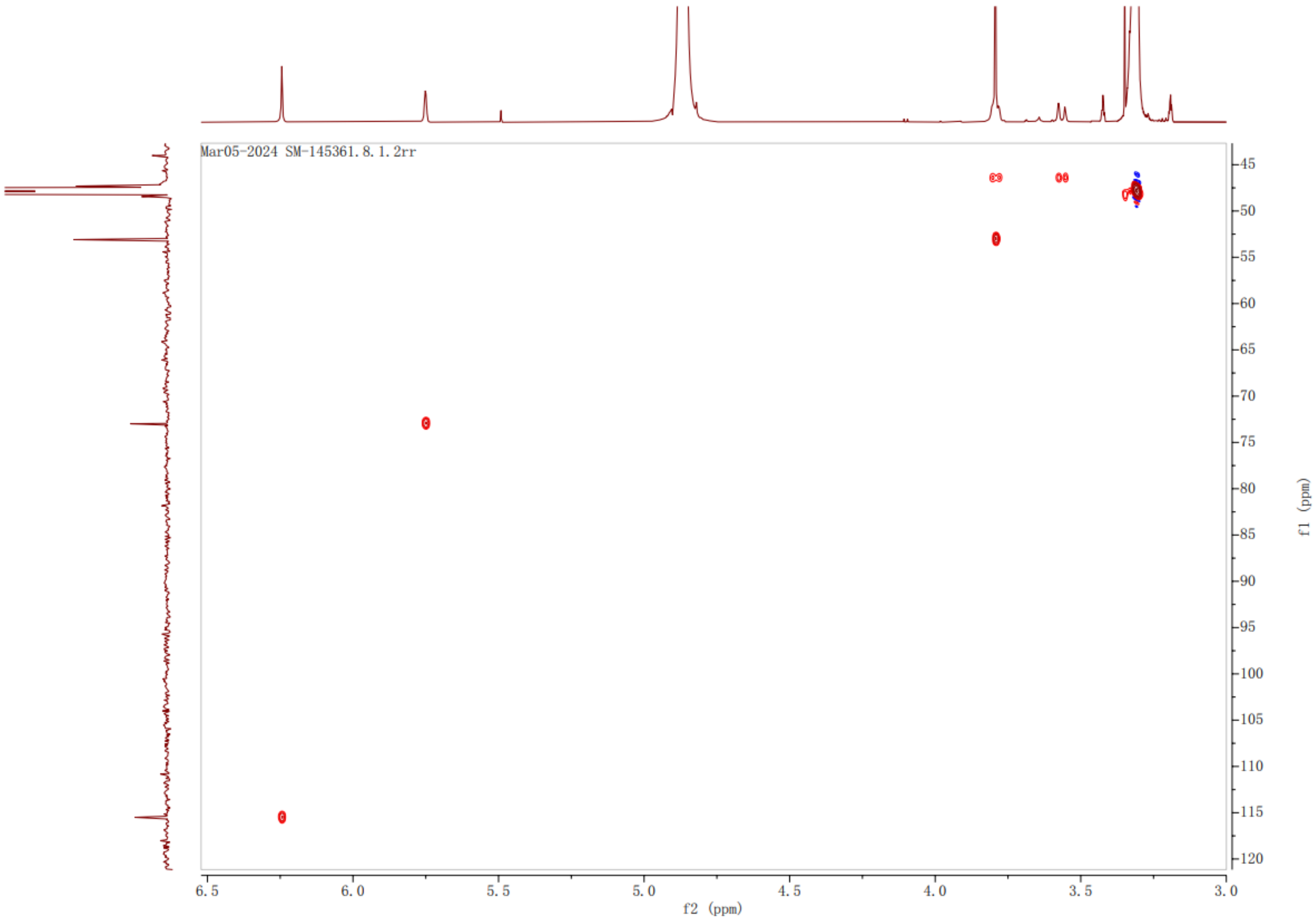

Figure S33. HMBC spectrum (600 MHz) of **2** in Methanol-d_4.

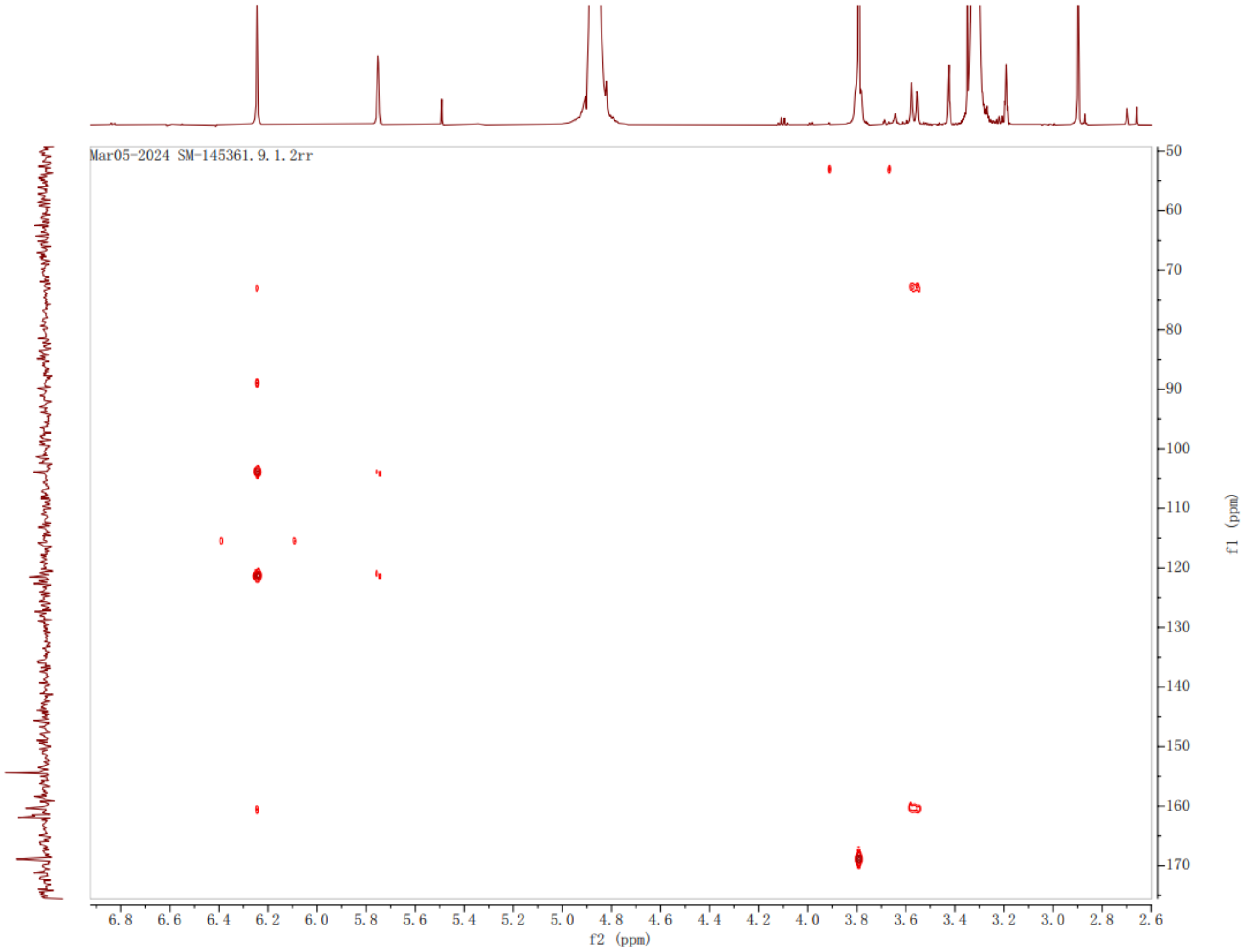

Figure S34. HR-ESI-MS spectrum of **2**.

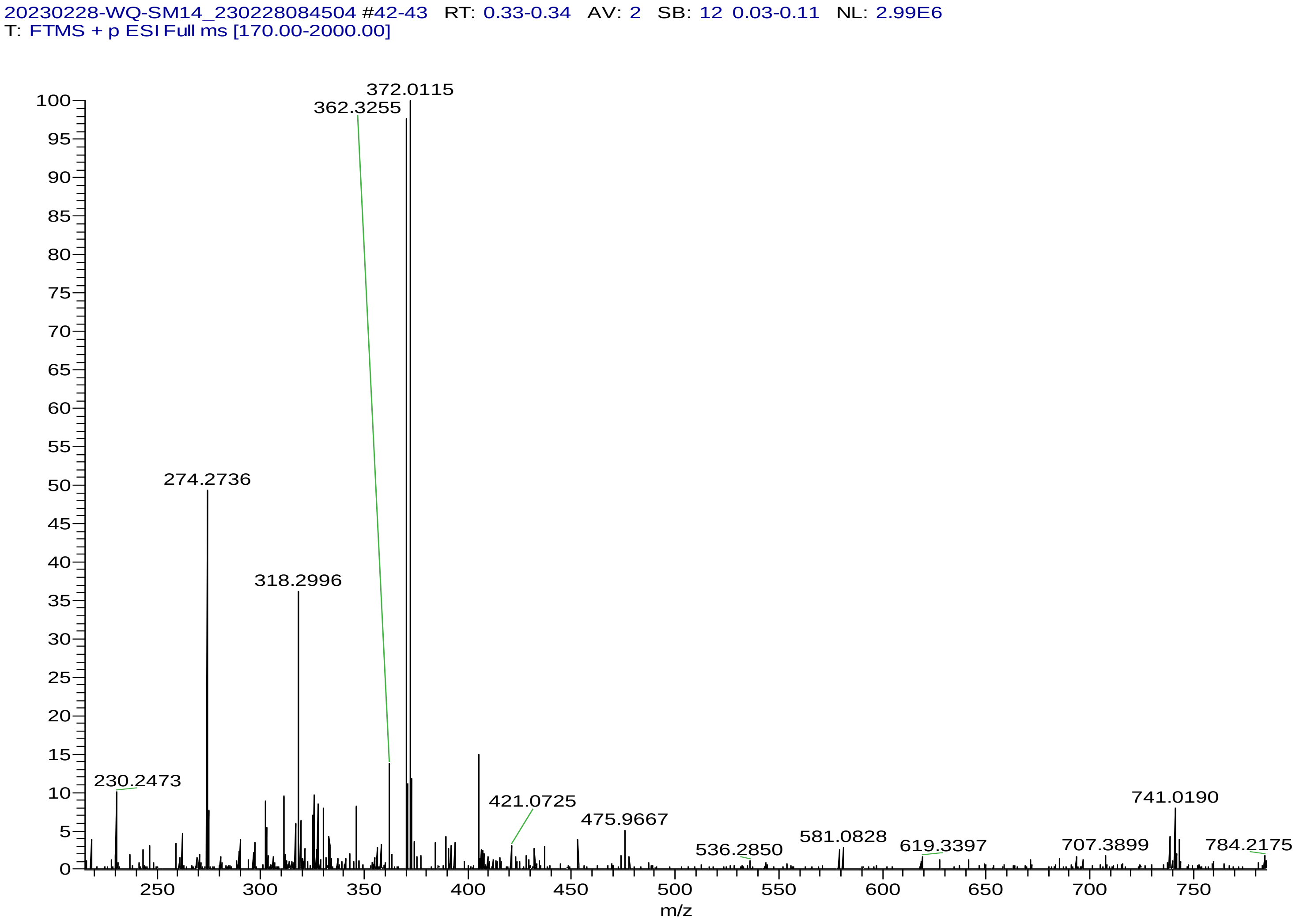

Figure S35. Close up view of HR-ESI-MS spectrum of **2**.

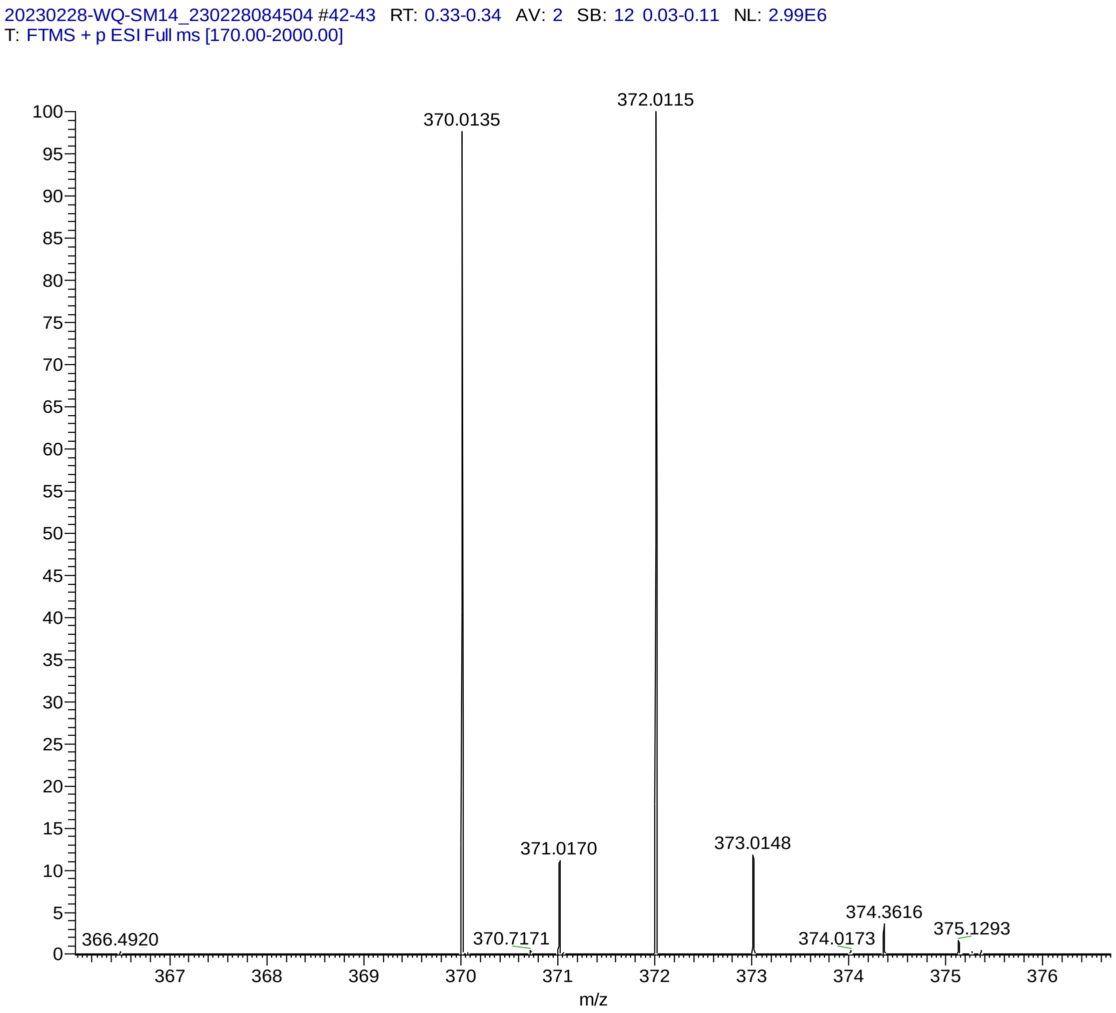

Figure S36. ^{1}H NMR spectrum (500 MHz) of **3** in DMSO-d_6.

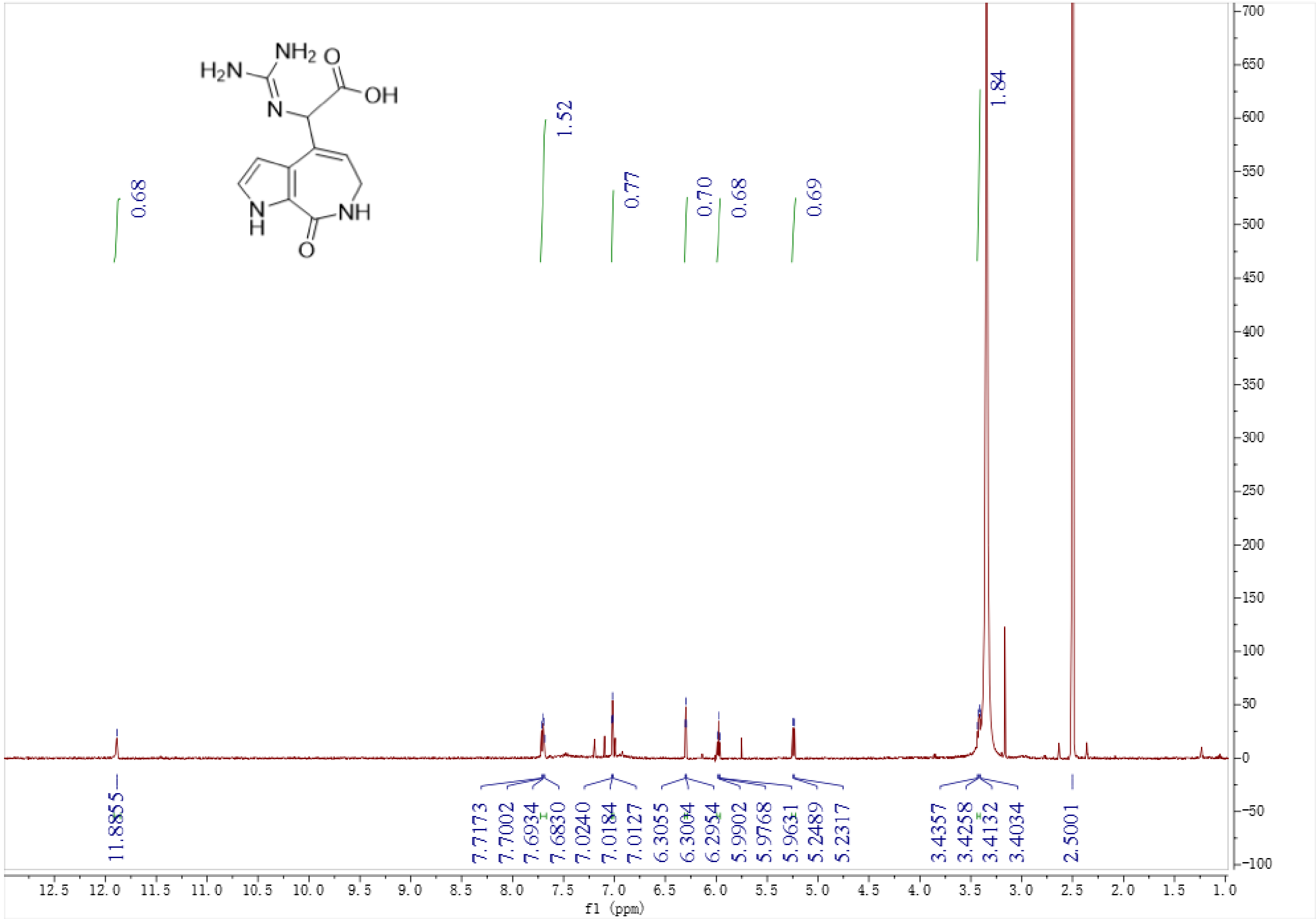

Figure S37. ^{13}C NMR spectrum (125 MHz) of **3** in DMSO-d_6.

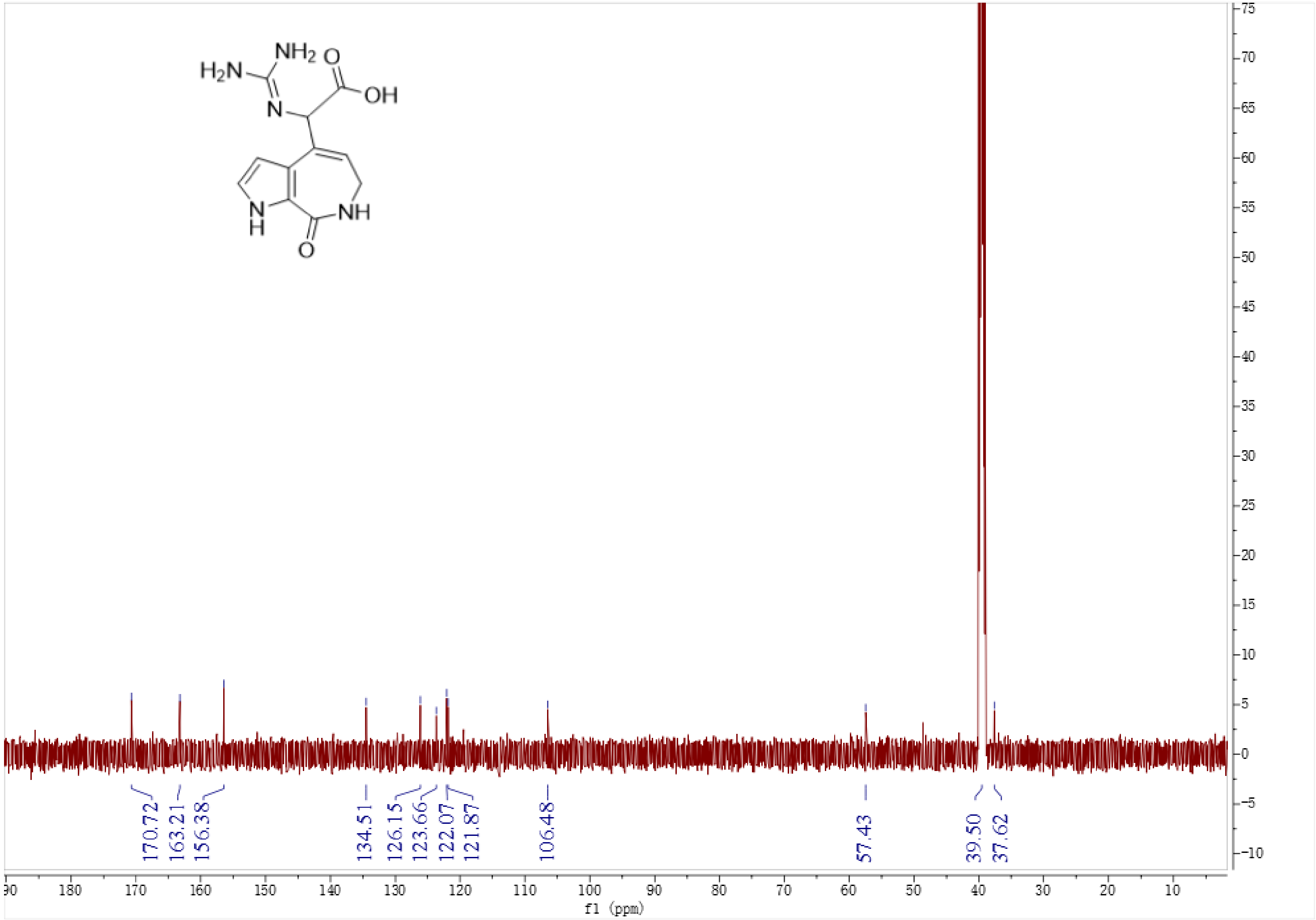

Figure S38. ^{13}C NMR and DEPT spectra (125 MHz) of **3** in DMSO-d_6.

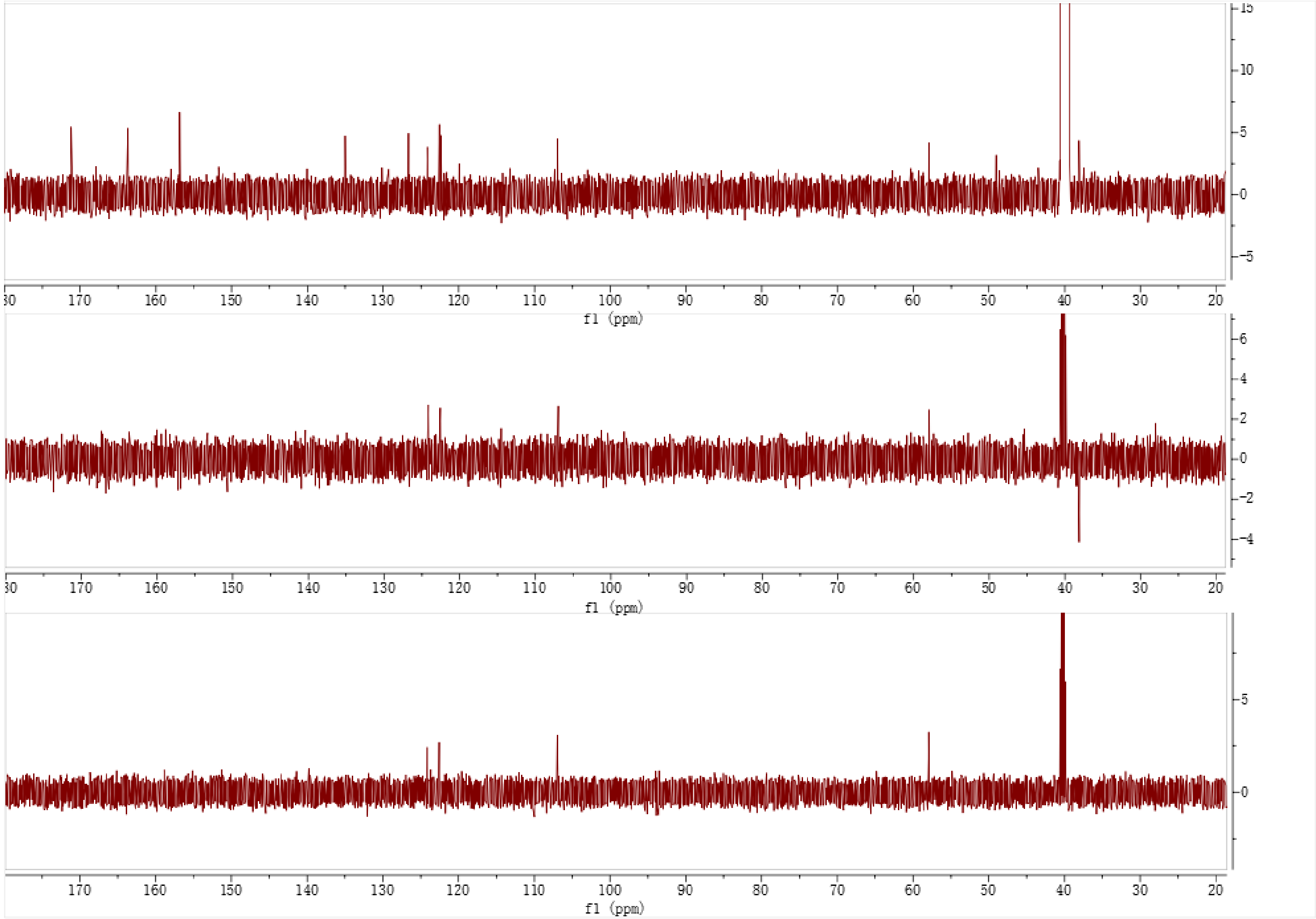

Figure S39. COSY spectrum (500 MHz) of **3** in DMSO-d_6.

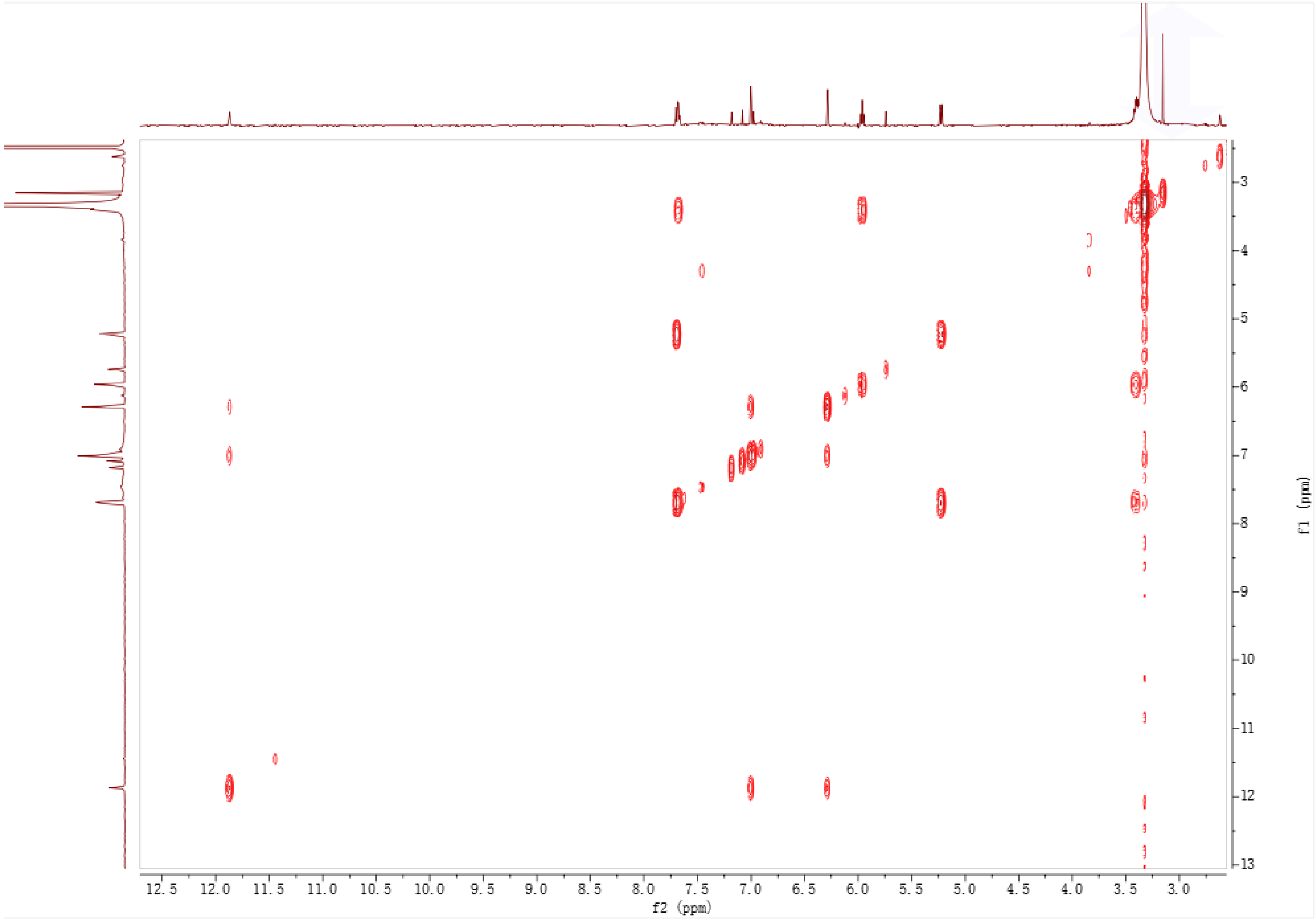

Figure S40. HSQC spectrum (500 MHz) of **3** in DMSO-d_6.

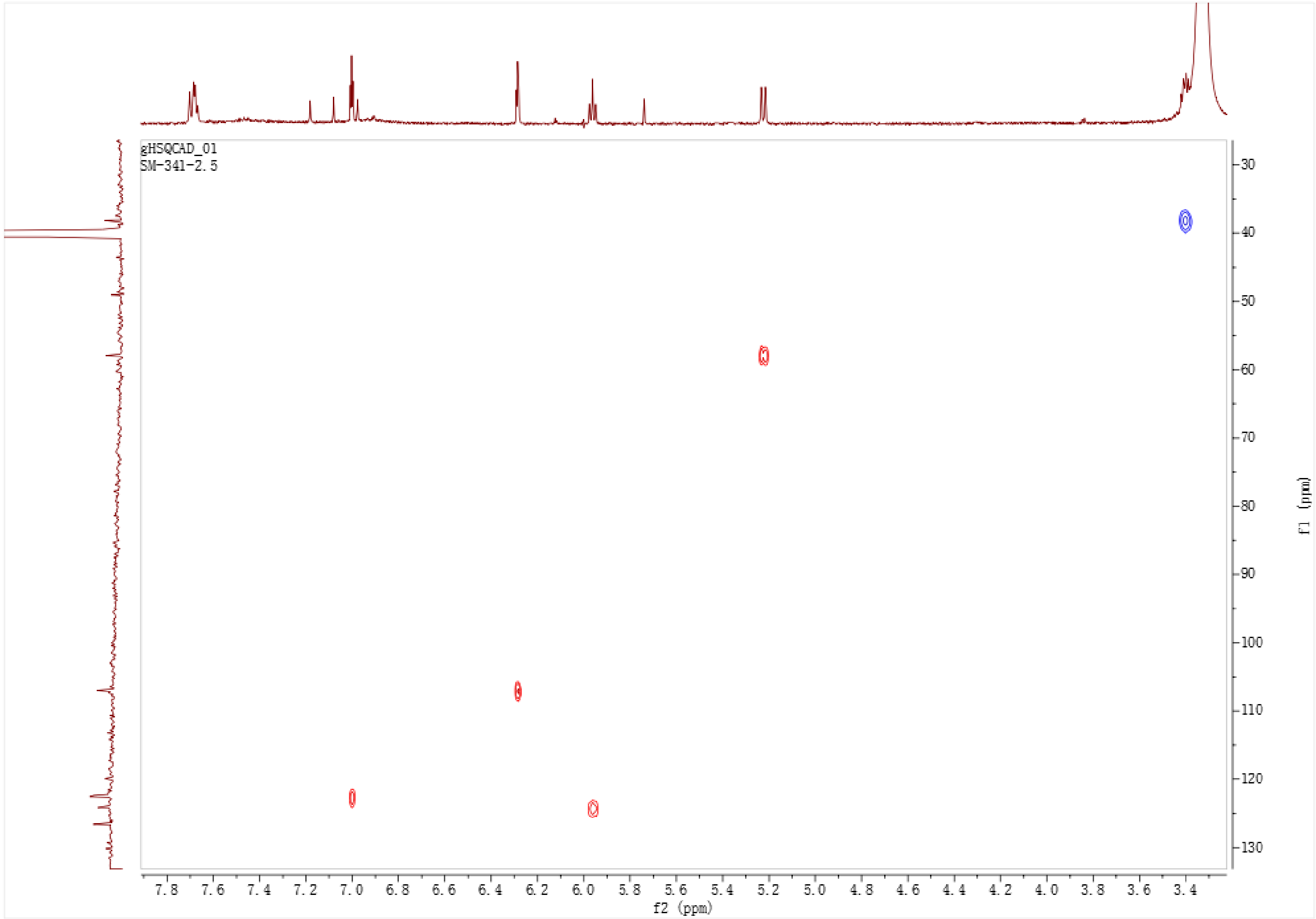

Figure S41. HMBC spectrum (500 MHz) of **3** in DMSO-d_6.

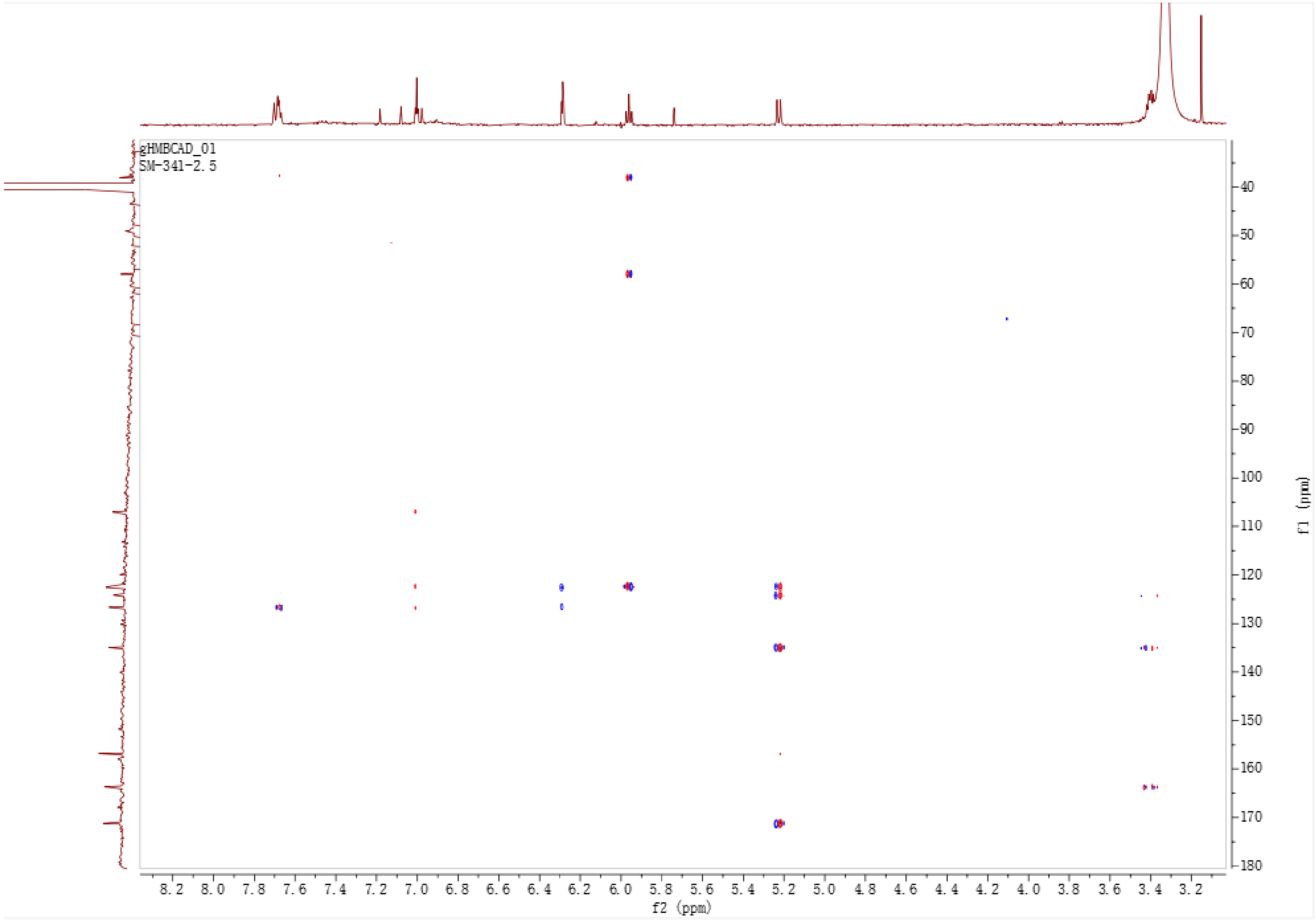

Figure S42. HR-ESI-MS spectrum of **3**.

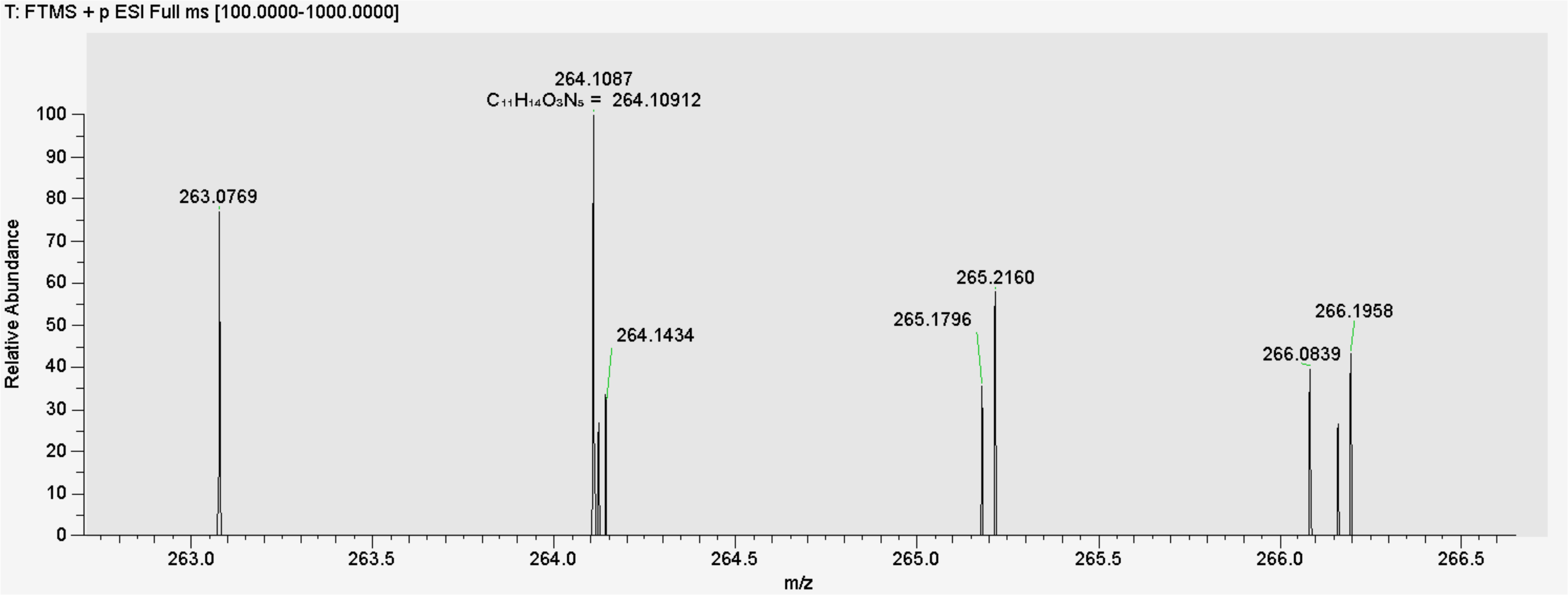

Figure S43. ^{1}H NMR spectrum (500 MHz) of **4** in DMSO-d_6.

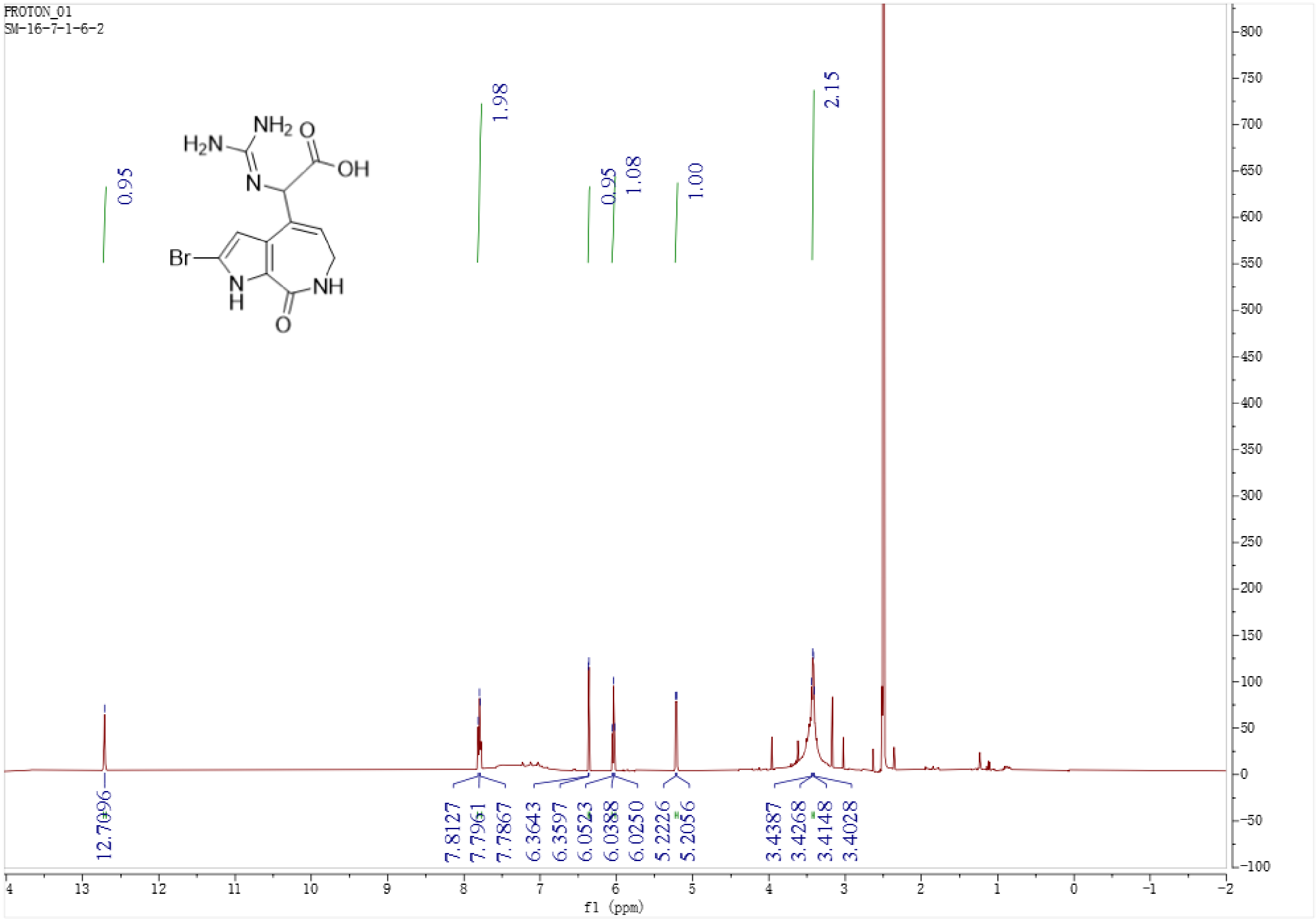

Figure S44. ^{13}C NMR spectrum (500 MHz) of **4** in DMSO-d_6.

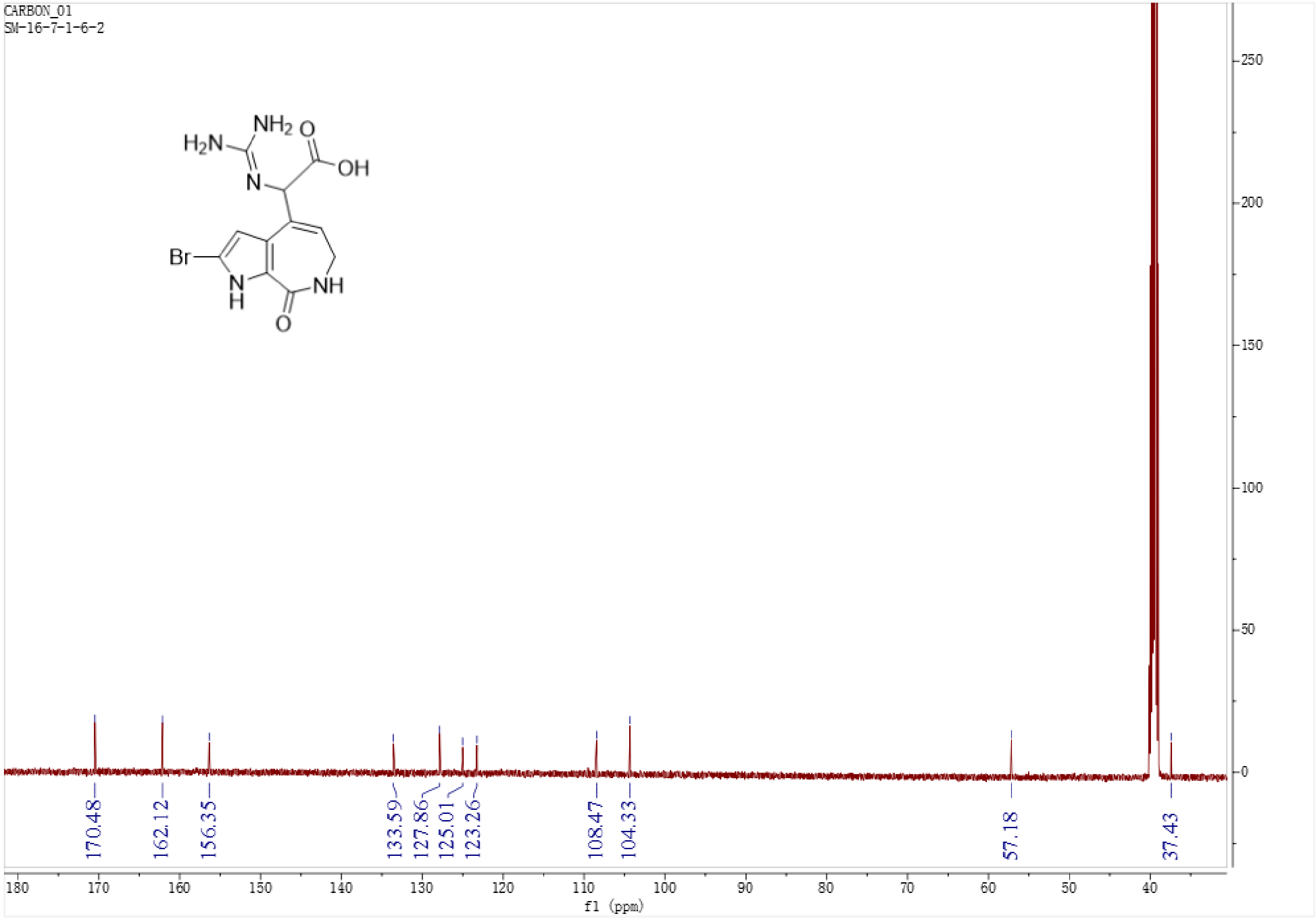

Figure S45. HR-ESI-MS spectrum of **4**.

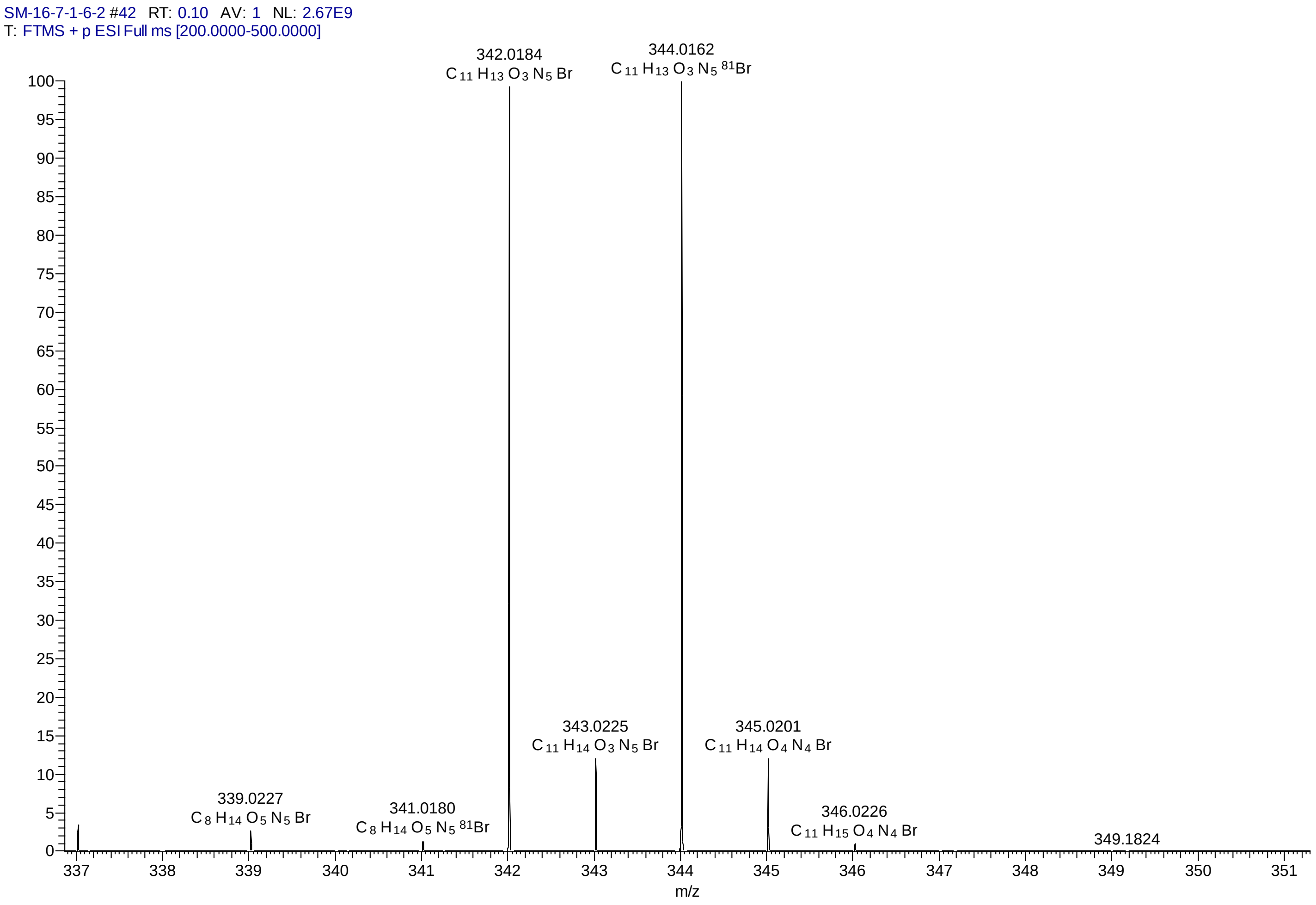

Figure S46. ^{1}H NMR spectrum (500 MHz) of **5** in DMSO-d_6.

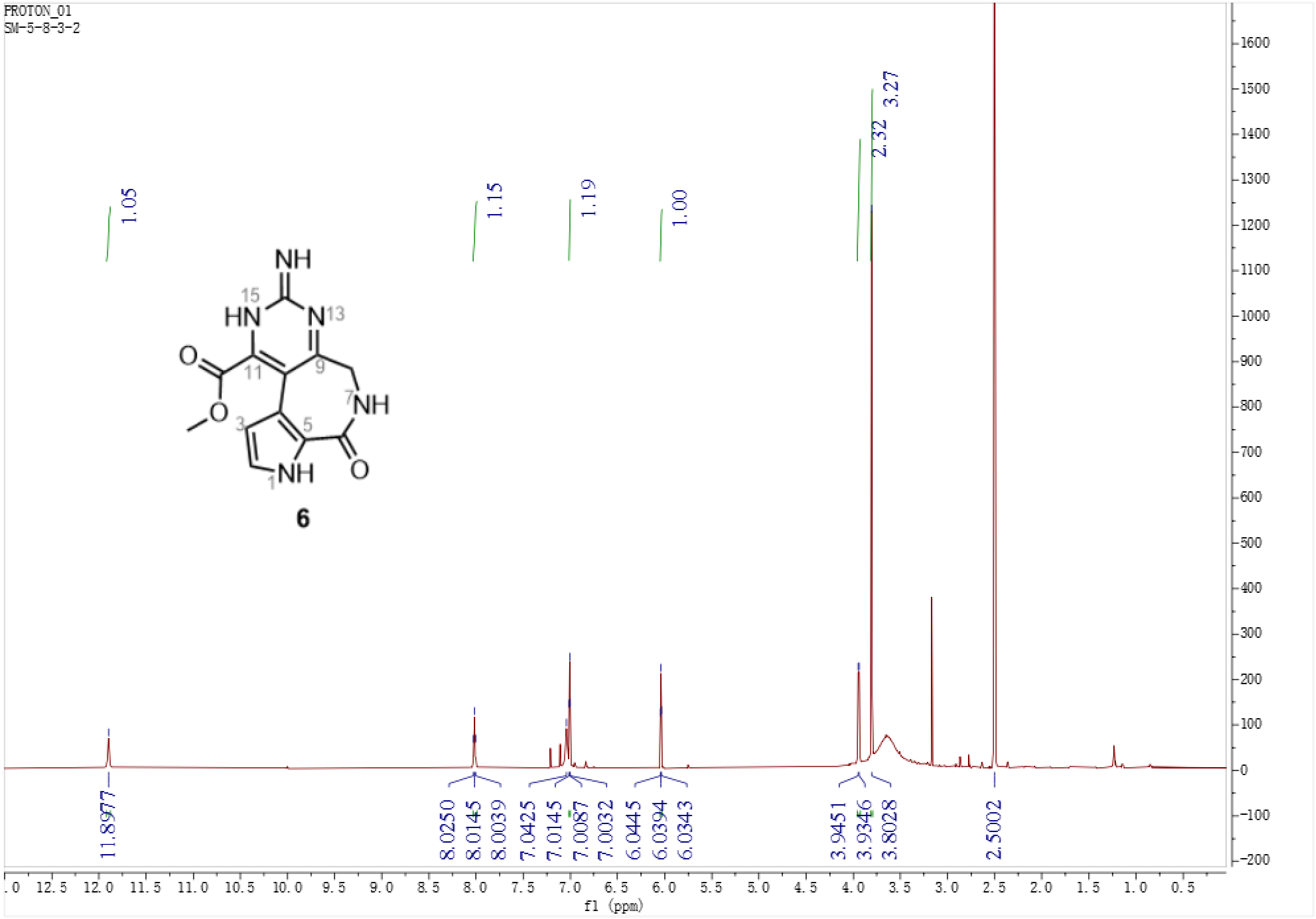

Figure S47. ^{13}C NMR spectrum (500 MHz) of **5** in DMSO-d_6.

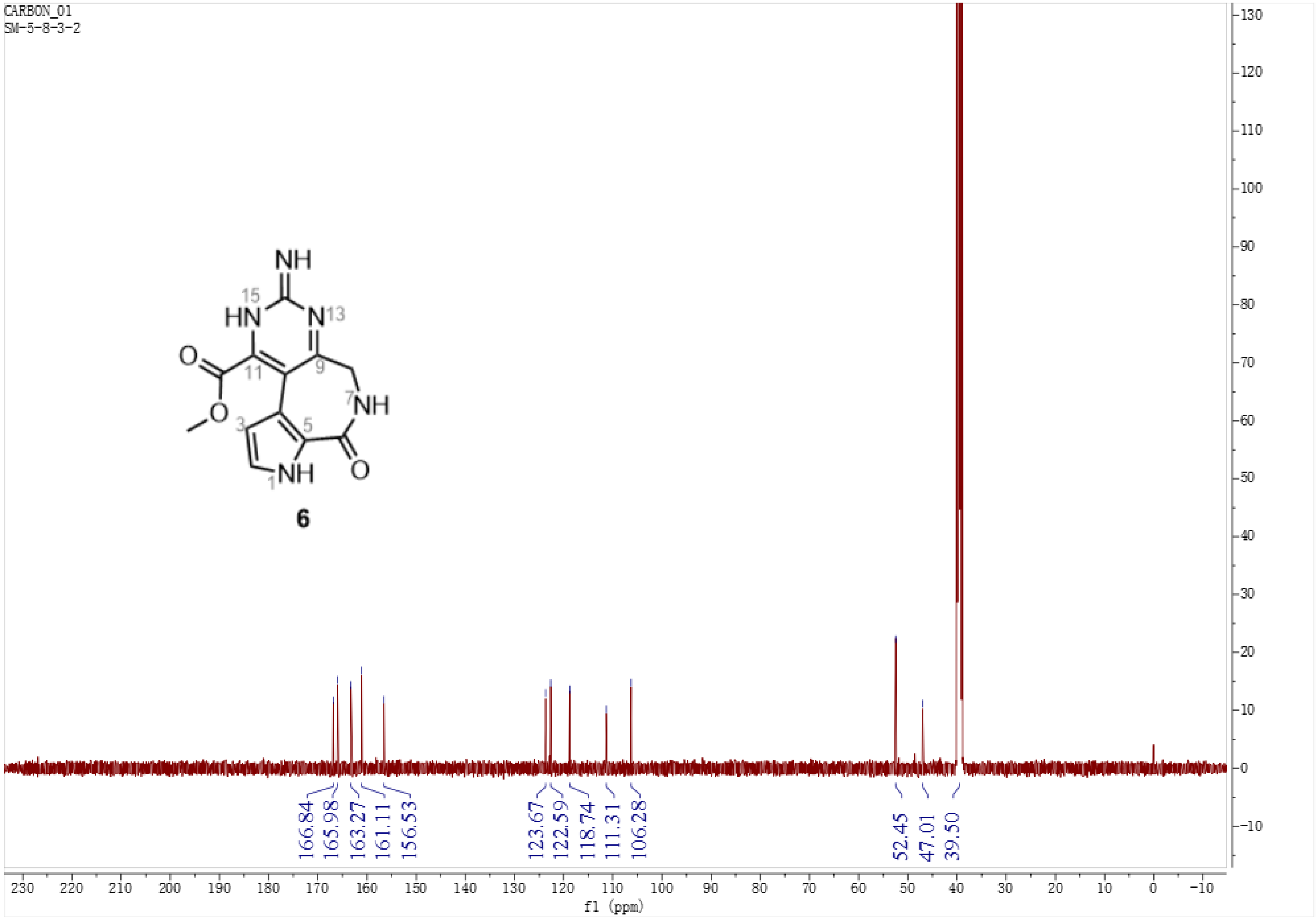

Figure S48. COSY spectrum (500 MHz) of **5** in DMSO-d_6.

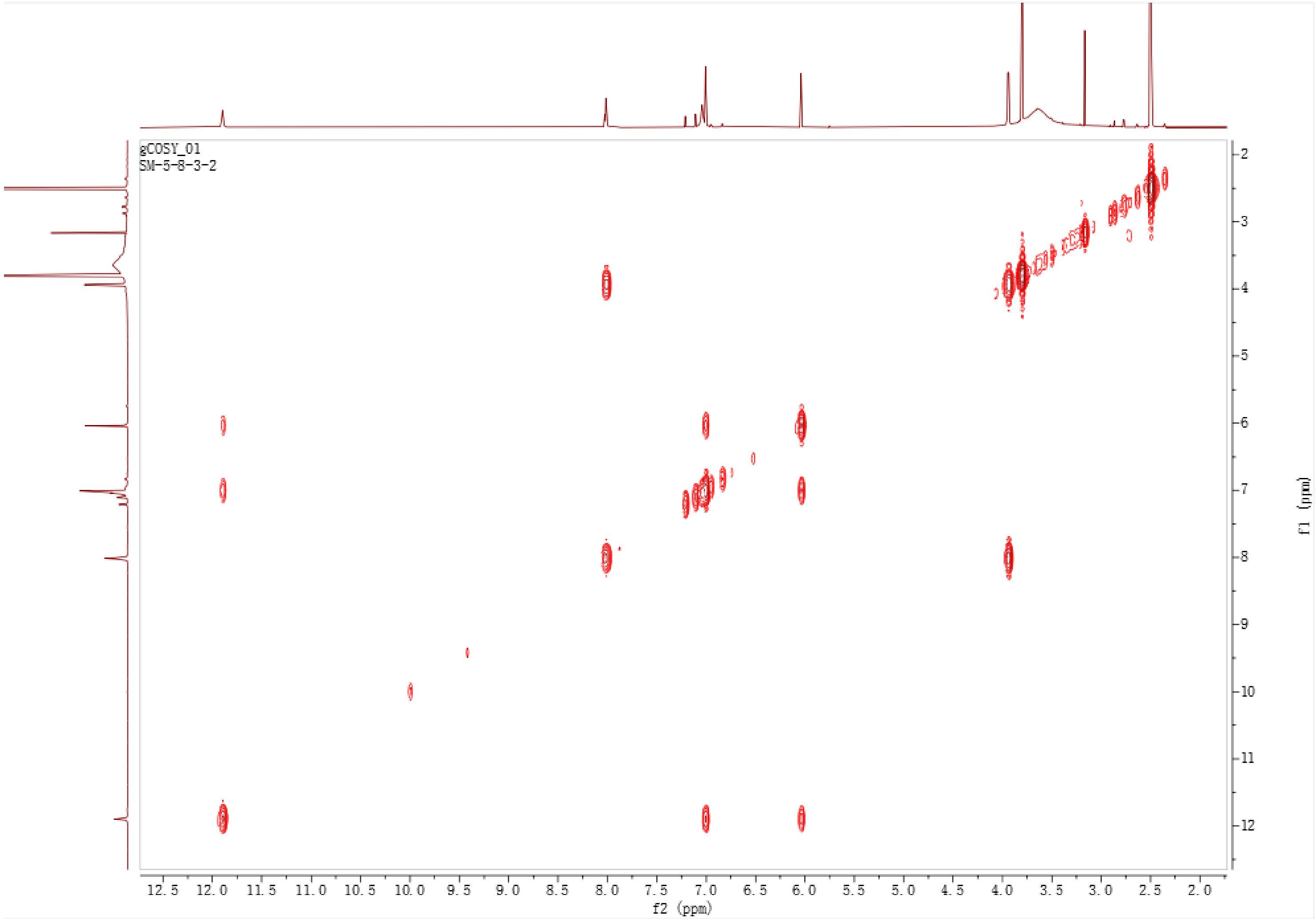

Figure S49. HSQC spectrum (500 MHz) of **5** in DMSO-d_6.

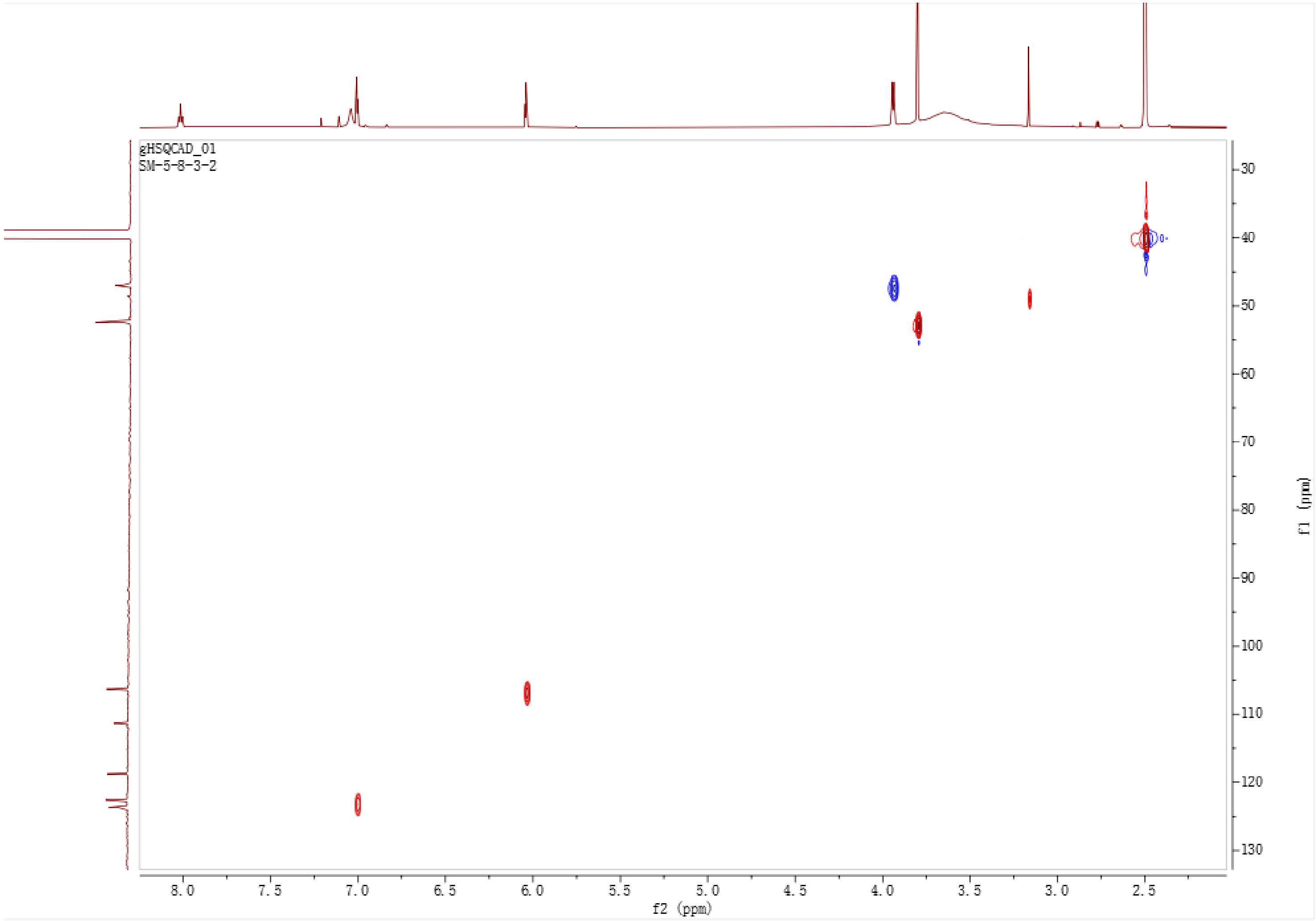

Figure S50. HMBC spectrum (500 MHz) of **5** in DMSO-d_6.

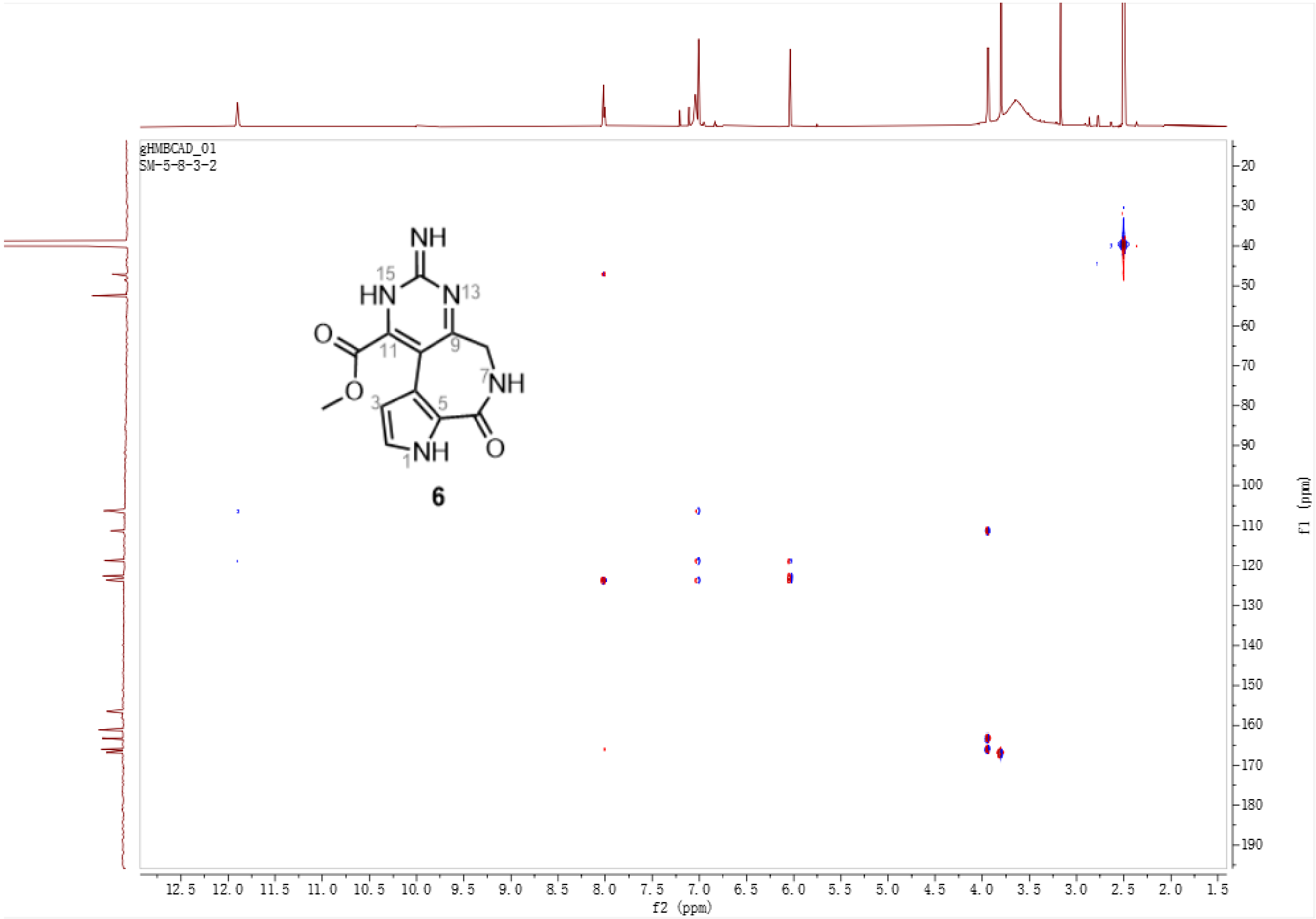

Figure S51. HR-ESI-MS spectrum of **5**.

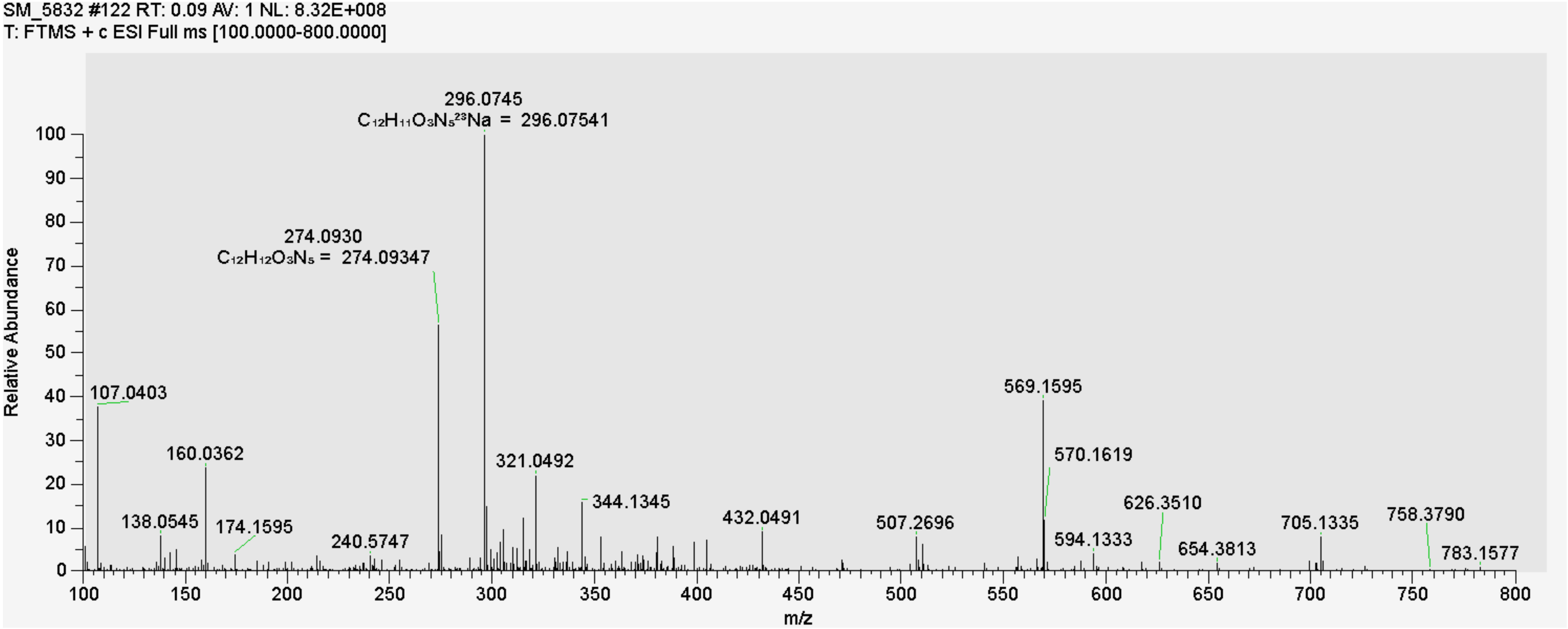

Figure S52. ^{1}H NMR spectrum (500 MHz) of **6** in DMSO-d_6.

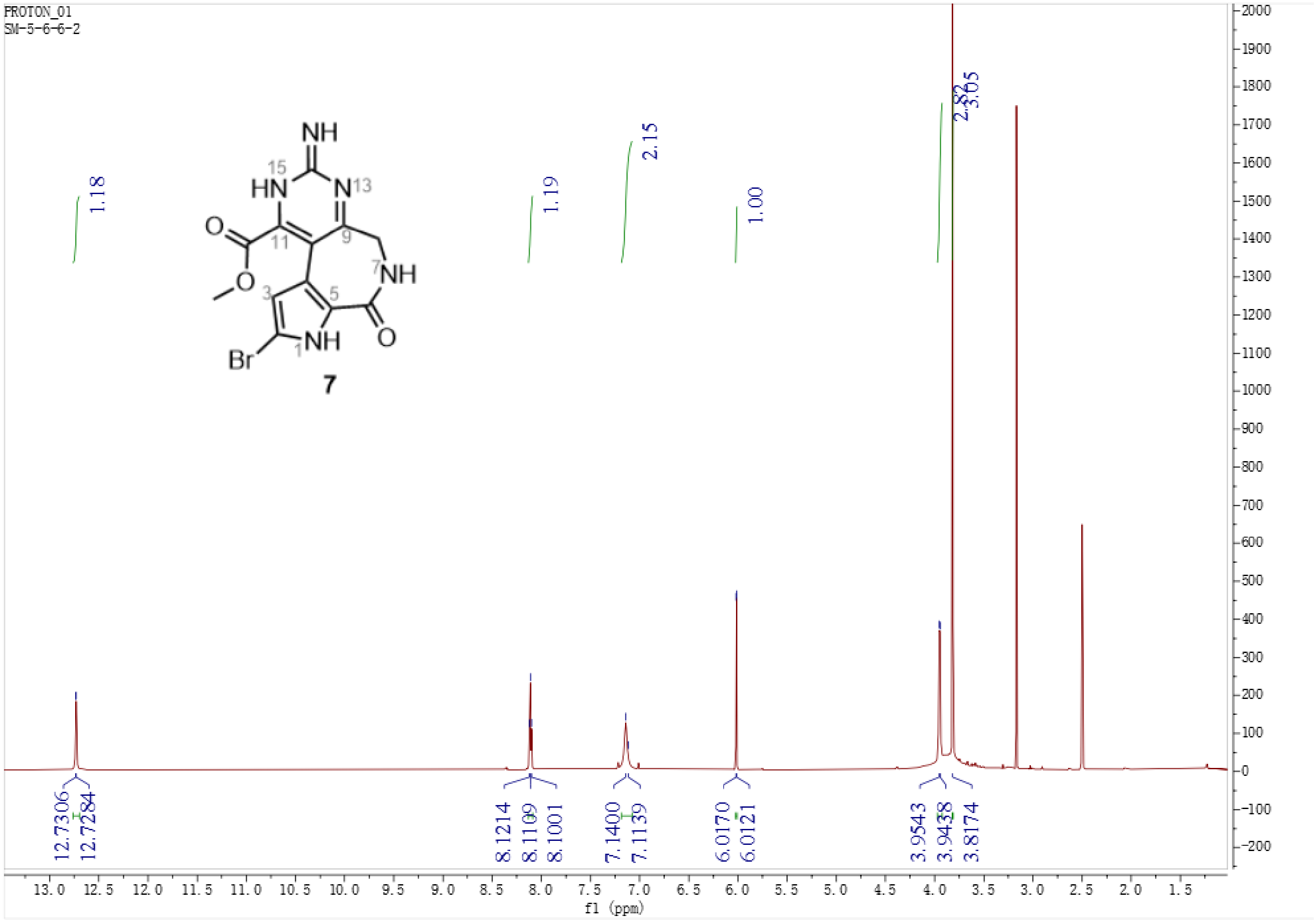

Figure S53. ^{13}C NMR spectrum (500 MHz) of **6** in DMSO-d_6.

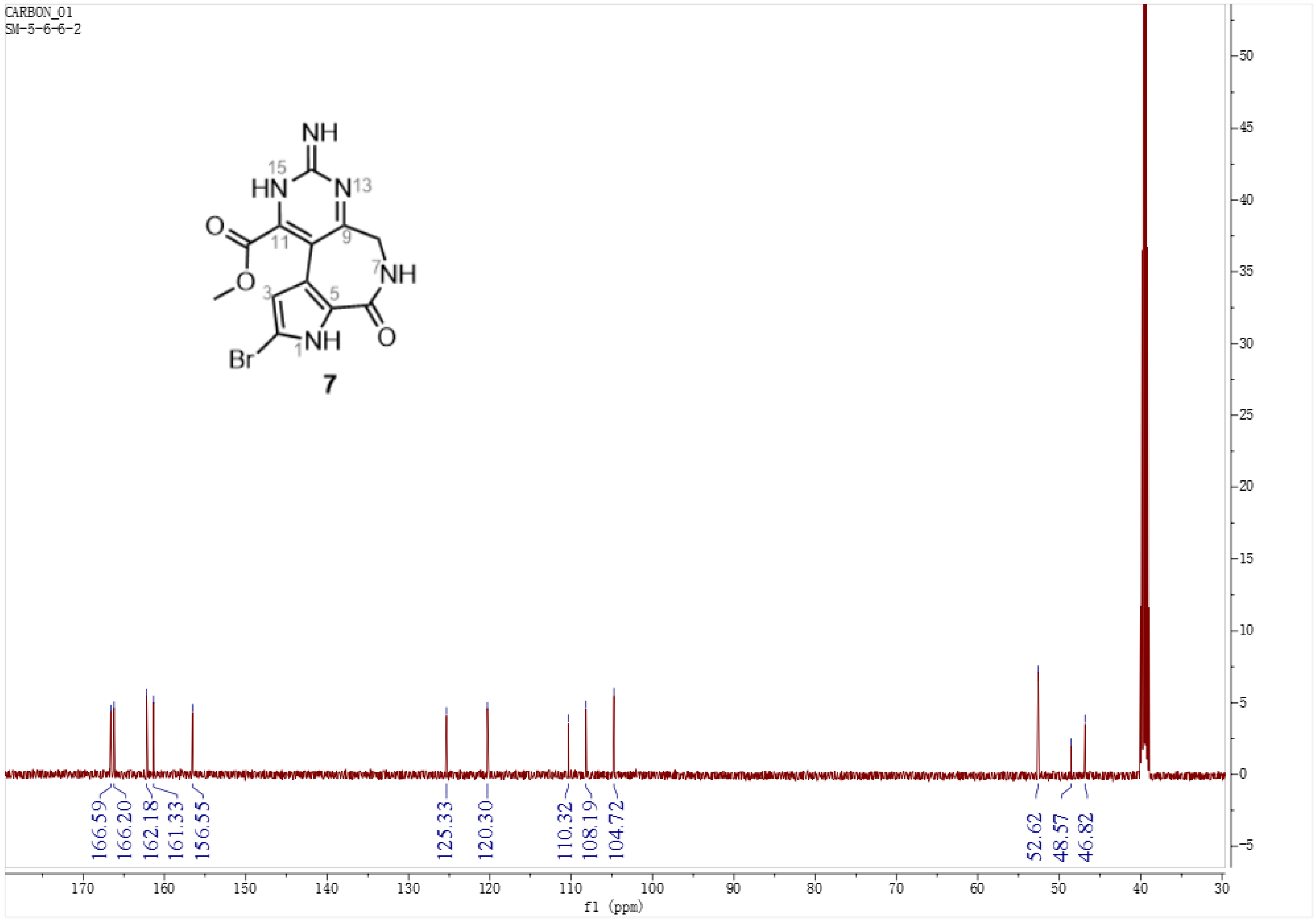

Figure S54. HR-ESI-MS spectrum of **6**.

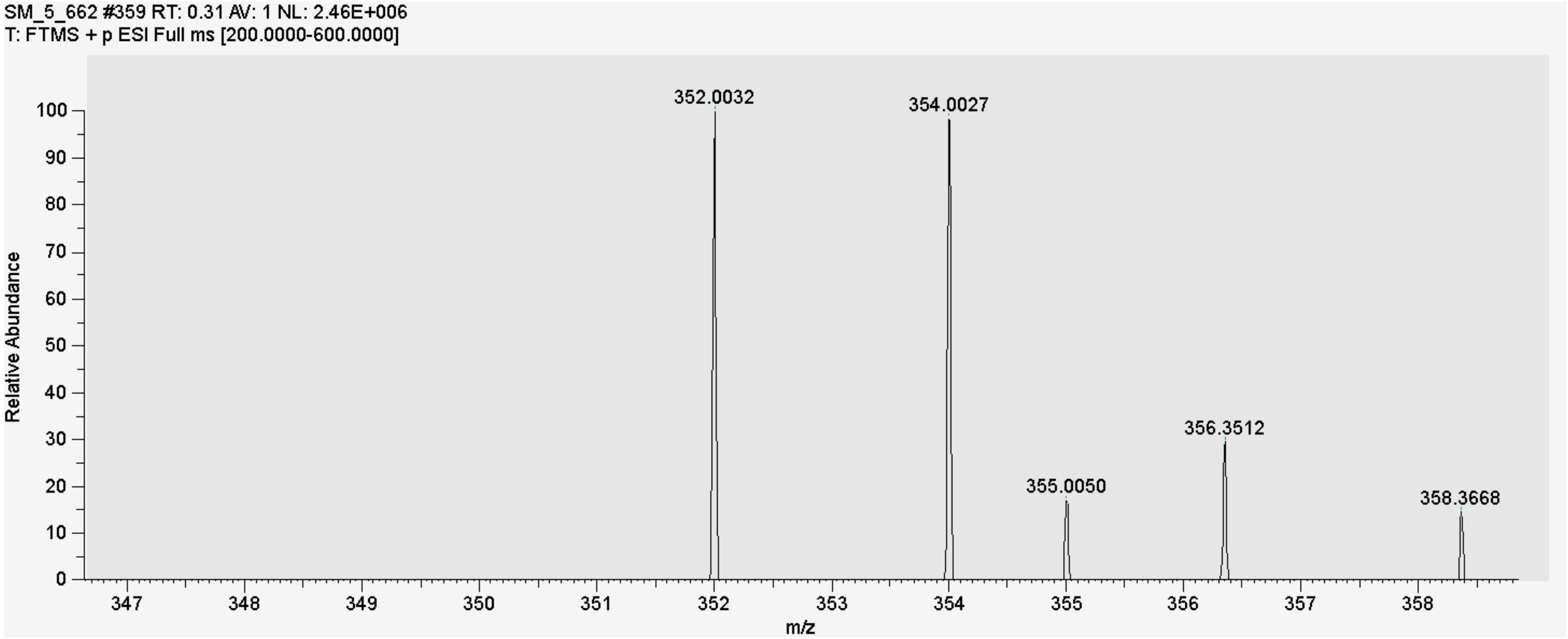

5. ECD Calculations Conformational analyses were carried out in the MMFF minimization force field by the Spartan 10 software package. Geometry re-optimization and frequency calculation were performed with the B3LYP functional with the 6-31+G (d, p) basis set using the Gaussian 09 software. ECD calculations using the TD-DFT method with the basis set RB3LYP/DGDZVP. The Boltzmann-averaged spectrum was obtained by GaussView 5.0.

Table S6. Cartesian coordinates for the optimized conformers of **1a** and **1b**.

B3LYP/6-3A1:J104(d,p) Energy / Hartree =-1039.584418 a.u. Population=83.2%

B3LYP/6-311G(d,p) Energy / Hartree =-1039.582396 a.u. Population=9.7%

Compound **1a1**		Standard Orientation			Compound **1a2**		Standard Orientation		
I	atom	X	Y	Z	I	atom	X	Y	Z
1	N	-2.52822	-0.77841	-0.46922	1	N	2.534671	0.704904	-0.55703
2	C	-1.38175	-0.09843	-0.07316	2	C	1.458489	-0.04972	-0.11508
3	C	-0.27378	-0.89329	-0.41083	3	C	0.283596	0.580852	-0.53872
4	C	-0.79625	-2.08097	-0.99153	4	C	0.688039	1.75502	-1.22991
5	C	-2.17346	-1.97793	-1.01266	5	C	2.069644	1.802768	-1.22173
6	C	-3.87973	-0.28499	-0.16807	6	C	3.924307	0.352846	-0.22683
7	C	-3.75861	1.235892	-0.16775	7	C	3.931787	-1.17011	-0.10932
8	O	-2.77551	1.668841	0.782336	8	O	2.993894	-1.60976	0.883283
9	C	-1.49724	1.137251	0.689777	9	C	1.674583	-1.20272	0.753856
10	N	-4.45855	-0.74392	1.077767	10	N	4.46057	0.953605	0.976779
11	O	-0.60517	1.696668	1.289036	11	O	0.82905	-1.76529	1.409045
12	C	1.148286	-0.60395	-0.20529	12	C	-1.08452	0.08049	-0.3603
13	C	2.014518	-1.67404	0.229602	13	C	-1.38193	-1.29074	-0.7124
14	N	3.355048	-1.25251	0.478917	14	N	-2.73935	-1.65523	-0.45207
15	C	3.792696	0.01802	0.299414	15	C	-3.67358	-0.8015	0.029349
16	N	3.020476	0.968267	-0.16637	16	N	-3.40983	0.45652	0.294477
17	C	1.732343	0.637573	-0.44679	17	C	-2.12796	0.867772	0.105893
18	N	5.111414	0.290178	0.564402	18	N	-4.92897	-1.28928	0.283189
19	O	1.74638	-2.85872	0.405093	19	O	-0.64464	-2.13598	-1.19899
20	O	0.980418	1.584091	-1.02241	20	O	-1.86008	2.156527	0.400713
21	C	1.467038	2.93059	-1.03573	21	C	-2.91037	2.960925	0.944023
22	H	-0.21037	-2.91409	-1.34586	22	H	0.0302	2.47252	-1.69692
23	H	-2.92294	-2.66175	-1.38666	23	H	2.751248	2.521591	-1.65495
24	H	-4.5299	-0.60147	-0.99084	24	H	4.544703	0.658938	-1.07604
25	H	-4.69968	1.689869	0.144556	25	H	4.909601	-1.51734	0.226208
26	H	-3.49278	1.590553	-1.17037	26	H	3.691081	-1.62193	-1.07812

27	H	-4.42988	-1.75821	1.13403	27	H	4.336684	1.962098	0.960781
28	H	-3.91978	-0.38081	1.860968	28	H	3.961776	0.59751	1.789096
29	H	3.978522	-1.99878	0.7607	29	H	-2.94209	-2.63308	-0.61869
30	H	5.568724	-0.24373	1.289364	30	H	-5.26066	-2.07551	-0.25542
31	H	5.321298	1.278423	0.567857	31	H	-5.6144	-0.57309	0.474096
32	H	0.681865	3.509836	-1.52247	32	H	-2.45754	3.937817	1.118476
33	H	1.619495	3.293306	-0.01682	33	H	-3.74564	3.052047	0.244403
34	H	2.400791	3.010089	-1.5973	34	H	-3.28292	2.545477	1.884106

B3LYP/6-311G(d,p) Energy / Hartree =-1039.582093 a.u.

Population=7.1%

B3LYP/6-311G(d,p) Energy / Hartree =-1039.584413 a.u.

Population=83.1%

Compound **1a3**		Standard Orientation			Compound **1b1**		Standard Orientation		
I	atom	X	Y	Z	I	atom	X	Y	Z
1	N	2.523314	0.788565	0.107223	1	N	2.528173	-0.779043	-0.468729
2	C	1.348834	0.087845	-0.155494	2	C	1.381727	-0.098681	-0.073309
3	C	0.272911	0.909541	0.219815	3	C	0.273651	-0.893766	-0.410306
4	C	0.841731	2.12814	0.680157	4	C	0.7961	-2.08189	-0.990044
5	C	2.21591	2.025656	0.592952	5	C	2.173297	-1.979096	-1.011005
6	C	3.83716	0.273988	-0.258719	6	C	3.879689	-0.285006	-0.168629
7	C	3.711242	-1.251627	-0.229802	7	C	3.758224	1.235848	-0.169052
8	O	2.650437	-1.729292	-1.053992	8	O	2.775568	1.669044	0.781372
9	C	1.390159	-1.177651	-0.874358	9	C	1.497329	1.137163	0.689591
10	N	4.843679	0.819047	0.630832	10	N	4.45946	-0.742983	1.07702
11	O	0.445465	-1.755043	-1.366604	11	O	0.605535	1.696346	1.28941
12	C	-1.161765	0.618526	0.144782	12	C	-1.148476	-0.603939	-0.204984
13	C	-2.05597	1.666395	-0.288175	13	C	-2.014916	-1.673864	0.229704
14	N	-3.413879	1.240036	-0.408697	14	N	-3.355445	-1.252146	0.478413
15	C	-3.840899	-0.01271	-0.116906	15	C	-3.792931	0.018458	0.298863
16	N	-3.039186	-0.936022	0.354508	16	N	-3.020323	0.968668	-0.166356
17	C	-1.728118	-0.605593	0.493378	17	C	-1.732134	0.637861	-0.446368
18	N	-5.154464	-0.323173	-0.358343	18	N	-5.111924	0.290561	0.563089
19	O	-1.799508	2.83362	-0.565976	19	O	-1.747245	-2.858754	0.404816
20	O	-0.93498	-1.527743	1.054821	20	O	-0.980155	1.584138	-1.02196
21	C	-1.413503	-2.873801	1.146604	21	C	-1.466007	2.930788	-1.034653
22	H	0.286767	2.985472	1.026504	22	H	0.210146	-2.915253	-1.343701
23	H	3.001515	2.717556	0.853022	23	H	2.922655	-2.663392	-1.384363
24	H	4.087356	0.594898	-1.279483	24	H	4.529528	-0.601654	-0.991641
25	H	3.559964	-1.574206	0.811206	25	H	3.49178	1.589892	-1.171704
26	H	4.625582	-1.713593	-0.610817	26	H	4.699413	1.690041	0.142589
27	H	4.801748	0.374927	1.545576	27	H	3.921149	-0.379527	1.860424
28	H	5.773214	0.678731	0.248119	28	H	4.430944	-1.757262	1.134067
29	H	-4.032686	1.9425	-0.794153	29	H	-3.979255	-1.998319	0.759768
30	H	-5.840509	0.4159	-0.318076	30	H	-5.568594	-0.242391	1.289196
31	H	-5.442669	-1.20311	0.044526	31	H	-5.321584	1.278887	0.566102

32	H	-0.591487	-3.436643	1.590099	32	H	-0.679852	3.51016	-1.519601
33	H	-2.301754	-2.937911	1.779594	33	H	-2.398927	3.011341	-1.597513
34	H	-1.640748	-3.268294	0.15383	34	H	-1.619934	3.292625	-0.015648

B3LYP/6-311G(d,p) Energy / Hartree =-1039.582396 a.u.

Population=9.8%

B3LYP/6-311G(d,p) Energy / Hartree =-1039.582093 a.u.

Population=7.1%

Compound **1b2**		Standard Orientation			Compound **1b3**		Standard Orientation		
I	atom	X	Y	Z	I	atom	X	Y	Z
1	N	-2.534671	0.704904	-0.557028	1	N	-2.523314	0.788565	0.107223
2	C	-1.458489	-0.049724	-0.115076	2	C	-1.348834	0.087845	-0.155494
3	C	-0.283596	0.580852	-0.53872	3	C	-0.272911	0.909541	0.219815
4	C	-0.688039	1.75502	-1.229906	4	C	-0.841731	2.12814	0.680157
5	C	-2.069644	1.802768	-1.221726	5	C	-2.21591	2.025656	0.592952
6	C	-3.924307	0.352846	-0.226825	6	C	-3.83716	0.273988	-0.258719
7	C	-3.931787	-1.170109	-0.109322	7	C	-3.711242	-1.251627	-0.229802
8	O	-2.993894	-1.60976	0.883283	8	O	-2.650437	-1.729292	-1.053992
9	C	-1.674583	-1.202721	0.753856	9	C	-1.390159	-1.177651	-0.874358
10	N	-4.46057	0.953605	0.976779	10	N	-4.843679	0.819047	0.630832
11	O	-0.82905	-1.765294	1.409045	11	O	-0.445465	-1.755043	-1.366604
12	C	1.084517	0.08049	-0.360301	12	C	1.161765	0.618526	0.144782
13	C	1.381929	-1.290738	-0.712396	13	C	2.05597	1.666395	-0.288175
14	N	2.739351	-1.655225	-0.452068	14	N	3.413879	1.240036	-0.408697
15	C	3.67358	-0.801504	0.029349	15	C	3.840899	-0.01271	-0.116906
16	N	3.40983	0.45652	0.294477	16	N	3.039186	-0.936022	0.354508
17	C	2.127958	0.867772	0.105893	17	C	1.728118	-0.605593	0.493378
18	N	4.928966	-1.289275	0.283189	18	N	5.154464	-0.323173	-0.358343
19	O	0.644643	-2.135975	-1.198987	19	O	1.799508	2.83362	-0.565976
20	O	1.86008	2.156527	0.400713	20	O	0.93498	-1.527743	1.054821
21	C	2.910369	2.960925	0.944023	21	C	1.413503	-2.873801	1.146604
22	H	-0.0302	2.47252	-1.696922	22	H	-0.286767	2.985472	1.026504
23	H	-2.751248	2.521591	-1.654952	23	H	-3.001515	2.717556	0.853022
24	H	-4.544703	0.658938	-1.07604	24	H	-4.087356	0.594898	-1.279483
25	H	-3.691081	-1.621927	-1.078115	25	H	-4.625582	-1.713593	-0.610817
26	H	-4.909601	-1.517339	0.226208	26	H	-3.559964	-1.574206	0.811206
27	H	-3.961776	0.59751	1.789096	27	H	-5.773214	0.678731	0.248119
28	H	-4.336684	1.962098	0.960781	28	H	-4.801748	0.374927	1.545576
29	H	2.942091	-2.633082	-0.618692	29	H	4.032686	1.9425	-0.794153
30	H	5.26066	-2.075508	-0.255419	30	H	5.840509	0.4159	-0.318076
31	H	5.614401	-0.573085	0.474096	31	H	5.442669	-1.20311	0.044526
32	H	2.457543	3.937817	1.118476	32	H	0.591487	-3.436643	1.590099
33	H	3.28292	2.545477	1.884106	33	H	1.640747	-3.268294	0.15383
34	H	3.745643	3.052047	0.244403	34	H	2.301754	-2.937911	1.779594

Table S7. Cartesian coordinates for the optimized conformers of **2a** and **2b**.

B3LYP/6-311G(d,p) Energy / Hartree =-3610.697819 a.u. B3LYP/6-311G(d,p) Energy / Hartree =-3610.699537 a.u.

Population=14% Population=85%

Compound **2a** Conf. 1		Standard Orientation			Compound **2a** Conf. 2		Standard Orientation		
I	atom	X	Y	Z	I	atom	X	Y	Z
1	C	-4.488305	-0.006836	-0.284725	1	C	4.430597	-0.822446	-0.183143
2	N	-3.915155	-1.00351	0.34874	2	N	3.948666	0.218771	0.452462
3	C	-2.557106	-1.034578	0.343458	3	C	2.598135	0.309217	0.558769
4	C	-1.728355	-0.089057	-0.24553	4	C	1.684278	-0.594609	0.018496
5	C	-2.352811	0.982271	-0.991317	5	C	2.211404	-1.788064	-0.5983
6	N	-3.779695	0.946502	-0.934727	6	N	3.636722	-1.805906	-0.673281
7	N	-5.856615	0.052574	-0.339276	7	N	5.780067	-0.912845	-0.391922
8	O	-1.974844	-2.066472	0.986129	8	O	2.11906	1.330047	1.278688
9	O	-1.826435	1.862517	-1.656267	9	O	1.60323	-2.749328	-1.058642
10	C	-0.264399	-0.153526	-0.189092	10	C	0.230872	-0.418635	0.071178
11	C	0.554387	-1.283358	-0.462485	11	C	-0.705122	-1.460269	0.320247
12	C	1.865076	-0.867552	-0.357385	12	C	-1.960474	-0.894092	0.28147
13	N	1.907645	0.457017	-0.041994	13	N	-1.864263	0.439641	0.026535
14	C	0.599562	0.910601	0.088346	14	C	-0.514804	0.75344	-0.132738
15	C	3.080005	1.31672	0.218738	15	C	-2.948537	1.413046	-0.207925
16	C	2.597875	2.737954	-0.06218	16	C	-2.329512	2.775085	0.087624
17	O	1.445937	3.067854	0.726229	17	O	-1.155112	3.001777	-0.702545
18	C	0.338467	2.247263	0.617805	18	C	-0.133737	2.071223	-0.627693
19	O	-0.72545	2.643696	1.032573	19	O	0.962841	2.389181	-1.032307
20	Br	3.450141	-1.860454	-0.579435	20	Br	-3.640093	-1.714244	0.515657
21	N	3.654231	1.203062	1.538128	21	N	-3.544836	1.375773	-1.522074
22	C	-2.814874	-3.020879	1.643072	22	C	3.00426	2.404925	1.615088
23	H	-4.2315	1.675839	-1.47245	23	H	4.012596	-2.599828	-1.176768
24	H	-6.292157	0.957685	-0.44039	24	H	6.197109	-1.822562	-0.519035
25	H	-6.314768	-0.574872	0.305988	25	H	6.324306	-0.250548	0.141216
26	H	0.221003	-2.276998	-0.71579	26	H	-0.4741	-2.498619	0.490726
27	H	3.844459	1.042085	-0.51377	27	H	-3.729409	1.203055	0.528509
28	H	2.363618	2.84933	-1.126837	28	H	-2.081244	2.848576	1.152962
29	H	3.371362	3.452302	0.220598	29	H	-3.030954	3.565605	-0.180018
30	H	2.973103	1.472159	2.243928	30	H	-2.846562	1.579123	-2.233166
31	H	3.934852	0.24314	1.718793	31	H	-3.924689	0.451672	-1.708684
32	H	-2.12883	-3.745286	2.083557	32	H	2.384021	3.124014	2.150699
33	H	-3.415646	-2.547817	2.424246	33	H	3.822132	2.060075	2.252055
34	H	-3.484089	-3.516097	0.934743	34	H	3.411244	2.863582	0.711481

B3LYP/6-311G(d,p) Energy / Hartree =-3610.695569 a.u.

Population = 1%

B3LYP/6-311G(d,p) Energy / Hartree =-3610.697612 a.u.

Population = 9%

Compound **2a** Conf. 3		Standard Orientation			Compound **2b** Conf. 1		Standard Orientation		
I	atom	X	Y	Z	I	atom	X	Y	Z
1	C	-4.441511	-0.819674	-0.042074	1	C	4.490103	-0.00944	-0.290513
2	N	-3.93745	0.265395	0.493507	2	N	3.915914	-1.014433	0.329758
3	C	-2.58419	0.351631	0.562582	3	C	2.557895	-1.029335	0.347728
4	C	-1.690203	-0.583809	0.042641	4	C	1.729408	-0.085448	-0.244654
5	C	-2.239629	-1.819038	-0.464267	5	C	2.354406	0.989816	-0.983778
6	N	-3.665984	-1.838735	-0.486332	6	N	3.781898	0.953673	-0.926356
7	N	-5.802698	-0.966742	-0.107958	7	N	5.85464	0.097292	-0.266045
8	O	-2.081641	1.41263	1.204091	8	O	1.974717	-2.054869	0.9997
9	O	-1.648589	-2.81714	-0.864832	9	O	1.828837	1.87991	-1.636091
10	C	-0.237463	-0.403681	0.026876	10	C	0.265367	-0.150632	-0.188048
11	C	0.713944	-1.43056	0.263378	11	C	-0.550485	-1.282318	-0.463316
12	C	1.963271	-0.865544	0.135437	12	C	-1.862238	-0.869841	-0.360101
13	N	1.855008	0.464751	-0.150953	13	N	-1.908421	0.454458	-0.044063
14	C	0.491292	0.761083	-0.260316	14	C	-0.601579	0.911238	0.088554
15	C	2.917801	1.472013	-0.400603	15	C	-3.083463	1.311323	0.214067
16	C	2.235248	2.840034	-0.365978	16	C	-2.604066	2.733634	-0.066013
17	O	1.051538	2.91985	-1.152354	17	O	-1.455308	3.066173	0.725607
18	C	0.056067	2.002644	-0.890319	18	C	-0.345306	2.248181	0.620468
19	O	-1.070423	2.250627	-1.261625	19	O	0.715882	2.646423	1.03964
20	Br	3.615769	-1.769357	0.27202	20	Br	-3.444579	-1.86653	-0.585349
21	N	3.99866	1.509378	0.549424	21	N	-3.660094	1.196678	1.532292
22	C	-2.958908	2.499453	1.520909	22	C	2.813953	-3.001417	1.668928
23	H	-4.063014	-2.707538	-0.822037	23	H	4.231615	1.743435	-1.372655
24	H	-6.186343	-1.535941	-0.848318	24	H	6.318835	0.593462	-1.0118
25	H	-6.308987	-0.113768	0.083084	25	H	6.326667	-0.728588	0.071603
26	H	0.499858	-2.465008	0.474595	26	H	-0.214351	-2.275161	-0.716216
27	H	3.338985	1.280352	-1.393716	27	H	-3.845683	1.034553	-0.519981
28	H	2.92407	3.588877	-0.75671	28	H	-3.380068	3.446219	0.214364
29	H	2.006485	3.090855	0.679613	29	H	-2.367203	2.845221	-1.130096
30	H	3.64333	1.576609	1.499907	30	H	-3.937936	0.235994	1.713143
31	H	4.574951	0.677518	0.493221	31	H	-2.981376	1.468647	2.239316
32	H	-2.32337	3.244552	2.000359	32	H	2.126924	-3.712892	2.128544
33	H	-3.399486	2.914037	0.611655	33	H	3.474511	-3.514677	0.965213
34	H	-3.752547	2.183551	2.201998	34	H	3.423776	-2.517001	2.435894

B3LYP/6-311G(d,p) Energy / Hartree =-3610.696789 a.u.

Population = 4%

B3LYP/6-311G(d,p) Energy / Hartree =-3610.699723 a.u.

Population = 87%

Compound **2b** Conf. 2		Standard Orientation			Compound **2b** Conf. 3		Standard Orientation		
I	atom	X	Y	Z	I	atom	X	Y	Z
1	C	-4.458819	0.008027	-0.456265	1	C	-4.428886	-0.819465	-0.186967
2	N	-3.94652	-0.904303	0.336707	2	N	-3.94588	0.229168	0.433718
3	C	-2.59035	-0.960441	0.409293	3	C	-2.595955	0.307198	0.560231
4	C	-1.712529	-0.124218	-0.262371	4	C	-1.683416	-0.595943	0.016999
5	C	-2.264553	0.848178	-1.177922	5	C	-2.212261	-1.794378	-0.589587
6	N	-3.694107	0.851444	-1.19016	6	N	-3.636732	-1.809827	-0.666287
7	N	-5.820828	0.085205	-0.592875	7	N	-5.786584	-0.960584	-0.311567
8	O	-2.064215	-1.898117	1.221947	8	O	-2.116855	1.324216	1.285115
9	O	-1.677998	1.614429	-1.927422	9	O	-1.604817	-2.764704	-1.031657
10	C	-0.251718	-0.204142	-0.117801	10	C	-0.230159	-0.420464	0.069827
11	C	0.576331	-1.312254	-0.436946	11	C	0.707379	-1.460857	0.317817
12	C	1.881421	-0.915136	-0.227759	12	C	1.961878	-0.89255	0.278557
13	N	1.907128	0.368423	0.227757	13	N	1.863542	0.441168	0.024222
14	C	0.596389	0.825921	0.297991	14	C	0.513518	0.752957	-0.133923
15	C	3.066489	1.252944	0.460999	15	C	2.945963	1.416616	-0.209888
16	C	2.600186	2.258197	1.510561	16	C	2.324299	2.77757	0.085441
17	O	1.403107	2.941454	1.114857	17	O	1.149207	3.002032	-0.704604
18	C	0.304102	2.18103	0.754218	18	C	0.129735	2.069682	-0.628903
19	O	-0.795003	2.681643	0.808255	19	O	-0.96784	2.385468	-1.033127
20	Br	3.475793	-1.875229	-0.514168	20	Br	3.642902	-1.709975	0.511521
21	N	3.583472	1.913268	-0.713303	21	N	3.542833	1.380332	-1.523856
22	C	-2.957965	-2.742059	1.954008	22	C	-3.003951	2.392368	1.63713
23	H	-4.10054	1.511021	-1.842088	23	H	-4.020899	-2.655269	-1.069788
24	H	-6.226366	0.976788	-0.837366	24	H	-6.142729	-1.481071	-1.099941
25	H	-6.327179	-0.4392	0.106114	25	H	-6.300872	-0.121473	-0.084037
26	H	0.254848	-2.279379	-0.79087	26	H	0.477666	-2.499452	0.48837
27	H	3.859701	0.63217	0.88647	27	H	3.727036	1.208292	0.526804
28	H	3.35863	3.03065	1.640763	28	H	3.024183	3.569361	-0.18247
29	H	2.433222	1.745603	2.465511	29	H	2.075914	2.850819	1.150738
30	H	3.862616	1.227174	-1.409297	30	H	3.925102	0.457105	-1.709881
31	H	2.86845	2.506586	-1.127976	31	H	2.844258	1.581561	-2.23524
32	H	-2.312344	-3.400726	2.536072	32	H	-2.385326	3.103786	2.18472
33	H	-3.598794	-2.157765	2.619578	33	H	-3.410351	2.864547	0.740162
34	H	-3.590066	-3.329699	1.28296	34	H	-3.822231	2.03664	2.267486
